# Improving the Activity of Aminophenoxazinones: Synthesis, CPC Purification, and Phytotoxicity Potential

**DOI:** 10.1021/acs.jafc.5c09345

**Published:** 2026-03-04

**Authors:** Cristina Díaz-Franco, Carlos Rial, Stefan Schwaiger, Rosa M. Varela, Francisco A. Macías, José M. G. Molinillo

**Affiliations:** † Allelopathy Group, Department of Organic Chemistry, Institute of Biomolecules (INBIO), Campus de Excelencia Internacional (ceiA3), School of Science, University of Cádiz, Puerto Real 11510, Spain; ‡ Institute of Pharmacy/Pharmacognosy and Center for Molecular Biosciences Innsbruck (CMBI), 16727 University of Innsbruck, Innrain 80-82, Innsbruck 6020, Austria

**Keywords:** 2-amino-3*H*-phenoxazin-3-one (APO), allelopathy, centrifugal partition chromatography, structure–activity relationship (SAR), natural product synthesis, *Lolium rigidum*, *Plantago lanceolata*, *Portulaca oleracea*

## Abstract

Aminophenoxazinones (APOs) are potent phytotoxic metabolites and histone deacetylase inhibitors, offering a low-resistance bioherbicide profile. This research synthesized a library of APO derivatives with targeted modifications to optimize physicochemical properties and biological activity using oxidative cyclocondensation. Purification challenges from strong compound-solid phase interactions were overcome by using centrifugal partition chromatography (CPC). Phytotoxicity was screened against *Lolium rigidum*, *Portulaca oleracea*, and *Plantago lanceolata*. While germination remained largely unaffected, compounds **10** and **11** caused significant root inhibition (77% and 89%, respectively for *P. oleracea*) at 1000 μM. Specifically, **11** reached inhibition levels comparable to the positive control, pendimethalin (86% for *P. oleracea*). Following a detailed structure–activity relationship analysis, **10** emerged as the most potent candidate, exhibiting an IC_50_ of 4.5 μM against *P. oleracea* roots, considerably lower than that of natural **APO** (332.5 μM). These findings strongly validate this rational design and purification strategy, positioning **10** as a valuable agrochemical lead for sustainable weed management.

## Graphical abstract

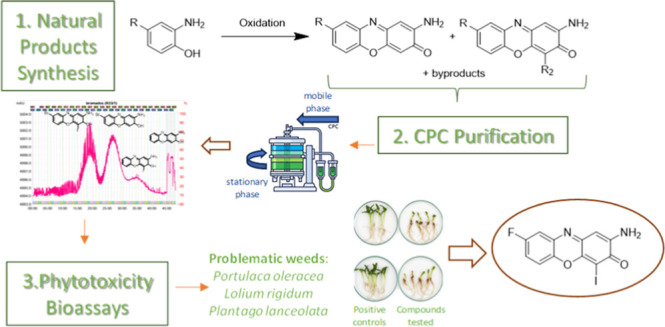

## Introduction

Aminophenoxazinones are degradation products of the benzoxazinone family, which are exuded by cereal crops of the Poaceae family,^1^ such as maize (*Zea mays* L.), wheat (*Triticum aestivum* L.), and rye (*Secale cereale* L.). Among all the natural products of this class, the compound 2.4-dihydroxy-2*H*-benzo­[*b*]­[1,4]­oxazin-3­(4*H*)-one (DIBOA) is the most common representative of this group and was one of the first hydroxamic acids to be isolated.^2^ In plants, it can be found as the corresponding β-glucoside. Plant tissue damage initiates an enzymatic conversion into DIBOA. When the compound finally reaches the soil, it has a short average lifetime (1 day approximately) due to degradation by soil microorganisms, leading to the formation of 2-amino-3*H*-phenoxazin-3-one (APO)^3^ through hydrolysis. Synthetic approaches to producing APO in higher yields proved to be significantly more effective than its isolation from natural sources. Especially, the synthesis via oxidative cyclocondensations resulted in high yields.^4^ However, challenges arise in the purification and handling of the products: APO and its derivatives have a strong interaction with common solid phases used as stationary phase in chromatographic techniques and show therefore low recovery rates during purification, and additionally, the solubility in water and other organic solvents is very low, which limits its production.

Notably, among all members of the benzoxazinone family, APO stands out for its phytotoxic activity, which is reflected by 76% inhibition of seedling growth;^5^ therefore its presence likely has a significant contribution to the allelopathic effect of species from the Poaceae family. Additionally, the mode of action of APO as allelochemical was demonstrated in *Arabidopsis thaliana* where APO can inhibit histone deacetylase (HDA) enzymes.^6^ HDAs are essential for numerous cellular functions^7^ and play crucial roles in many regulatory processes.^8^ The inhibition of these target structures disrupts the regulation of gene expression, ultimately leading to slowed growth and impaired development of the target plant. Moreover, HDAs are highly conserved throughout evolution, which renders target-site resistance (TSR)^9^ extremely difficult to achieve. This is primarily because mutations in the enzyme are highly improbable without severe phytotoxicity or lethality.^10^ When this intrinsic difficulty is combined with judicious, nonexcessive application and tank-mixed with herbicides exhibiting distinct modes of action (MOAs), the risk of weed resistance development can be substantially mitigated.^11^

Weeds have a significant impact on agricultural crops, reducing crop quality and productivity by up to 95%^12^ due to direct competition against crops for natural resources. However, despite the increase in crop productivity, the continued use of these herbicides has led to numerous disadvantages. Thus, the widespread application of synthetic herbicides presents significant agrochemical and environmental challenges, including high persistence in soil, which detrimentally alters edaphic ecology (e.g., earthworm populations) and inhibits crucial biogeochemical processes such as the nitrogen cycle, leading to the contamination of groundwater.^13–15^ Moreover, the inherent lack of specificity of these compounds results in their incorporation into the food chain, causing harm to nontarget organisms.^16^ Critically, the continuous use of agents with recurrent modes of action has precipitated a widespread resistance crisis;^17,18^ weeds have now developed resistance to 21 of the 31 known herbicide modes of action, affecting 168 different active ingredients, with resistance to acetolactate synthase (ALS) inhibitors being exceptionally prevalent among the 540 documented resistant weed species.^19^ In this context, natural products such as APO represent a viable alternative, being investigated as a potential bioherbicide to circumvent the profound disadvantages associated with their synthetic counterparts.

To systematically explore the structure–activity relationships (structure–activity relationship (SAR)) of the core scaffold, our strategy involved the synthesis of derivatives bearing three distinct functional groups: halogens, carboxylic acids, and nitrogen-containing heterocycles.

Halogenated derivatives were synthesized due to their prevalence in bioactive compounds. The inclusion of iodine is relevant, as it is found in commercial agrochemicals^20^ and can enhance hydrophobicity and membrane permeability. Brominated natural products, often from marine sources,^21^ exhibit significant bioactivities.^22^

Fluorine’s unique properties have proven successful in developing potent pharmaceuticals,^23^ with 20–25% of all drugs containing at least one fluorine atom.^24^ Chlorinated natural products are also found in diverse sources (algae, fungi, plants) and are known for their wide range of biological activities.^25–28^ Carboxylic acid groups were introduced to enhance polarity and aqueous solubility, key factors in modulating bioavailability following Lipinski’s rule of 5.^29^

Finally, nitrogen-containing heterocycles (NHNPs) were incorporated due to their association with diverse biological activities,^30–33^ the nitrogen atom within the heterocyclic ring imparts both weak basicity, due to its lone pair of electrons, and weak acidity via the N–H bond, significantly influencing the molecule’s activity and its physicochemical characteristics. Furthermore, the introduction of substituents, in our case, halogens, can modulate or enhance the biological properties of these NHNPs.

Given the challenges in purifying the synthesized derivatives, Centrifugal Partition Chromatography (CPC) was employed.^34^ CPC is a support-free, liquid–liquid chromatograph, where the mobile and stationary phases consist of two immiscible liquids forming a two-phase solvent system. The separation is based on the analytes’ partition coefficients.

Its primary advantage is the absence of a solid stationary phase (e.g., silica), which prevents irreversible sample adsorption and potential chemical degradation of the analytes. While CPC is widely established for the isolation of natural products,^35^ its application for the purification of complex synthetic chemistry products, as demonstrated in this work, remains less common.

The phytotoxic activity of the synthesized compounds will then be thoroughly evaluated using bioassays on three significant weed species: *Lolium rigidum*, *Portulaca oleracea*, and *Plantago lanceolata* *.*

## Materials and Methods

### General Experimental Procedures

2-Aminophenol, 2-amino-4-bromophenol, 2-amino-4-fluorophenol, 3-amino-4-hydroxybenzoic acid, 4-amino-3-hydroxybenzoic acid, 2-aminopyrindin-3-ol, 2-amino-4-bromophenol, 2-amino-4-chlorophenol, 2-amino-6-bromopyrindin-3-ol, sodium iodate (NaIO_3_), and *tert*-butyl hydroperoxide (TBHP) were purchased from Aldrich Chemical Co. (San Luis, Misuri, USA) *n*-Hexane, methanol, ethyl acetate, and 1,2-dimethoxyethane were obtained from VWR International (Radnor, PA, USA). The purities and structures of all of the compounds were determined via mass spectrometry and NMR. ^1^H NMR, ^1^H–^13^C gHSQC, ^1^H–^13^C gHMBC, and ^13^C NMR data were recorded at room temperature using DMSO-*d*_6_ as the solvent on Bruker (Karlsruhe, Germany) spectrometers at 500/125 and 600/150 MHz. Additionally, the resonance of residual dimethyl sulfoxide was set to δ 2.50 ppm for ^1^H and 39.5 ppm for ^13^C. The exact masses were measured on an ultraperformance liquid chromatography quadrupole time of fly electrospray ionization (UPLC-QTOF-ESI) (Water (Milford, Massachusetts, USA) Synapt G2) high-resolution mass spectrometer (HRTOFESIMIS). The reactions were monitored by thin-layer chromatography (TLC) using Merck–Kiesegel 60 F_254_ normal and reverse phase plates. The yields of all reactions and the theoretical partition coefficients of the shake flask experiments were calculated via the results of a high-performance liquid chromatography (HPLC) analysis (HP1100 system (Agilent, Waldbronn, Germany) equipped with an autosampler, a DAD, and a column thermostat); the column used was a Synergi MAX-RP (150 × 4.6 mm; 5 μm) The analysis conditions for HPLC were as follows: A: water + 0.1% formic acid and B: acetonitrile + 0.1% formic acid. The linear gradient was as follows: 0–5 min 50% B, 5–10 min 70% B, 10–15 min 90% B, and 15–20 min 98% B, and the purification process utilized three different CPC setups: a FCPC (fast CPC) system from Kromaton-Rousselet-Robatel (Annonay, France), featuring a 55 mL rotor and a 5 mL sample loop linked to a MERK HITACHI L-7100 pump (Tokyo, Japan), and a SuperFrac fraction collector from Pharmacia Biotech (Basilea, Sweden). Additionally, two Gilson CPC systems (Saint Avé, France) were employed, each paired with a Gilson PLC 2250 unit comprising a binary pump, a UV detector, and a fraction collector. One Gilson CPC had a 250 mL rotor with a 20 mL sample loop (mCPC), whereas the other had a 1000 mL rotor with a 10 mL sample loop (cCPC).

### General Methods for the Synthesis Using TBHP as an Oxidant

A powder of selenium oxide (0.5 equiv, SeO_2_) was added to the starting material dissolved in methanol. Then, TBHP (2 equiv) via a Hamilton syringe was added. The mixture was stirred at room temperature for 1 h. SeO_2_ was removed by filtering the crude material, and then, the solvent and the remaining TBHP were removed under reduced pressure.

#### Synthesis Using 2-Amino-4-bromophenol as the Starting Material (Reaction 1)

0.282 g of 1.5 mM of 2-amino-4-bromophenol (**2a**), 0.076 g of selenium oxide, and 290 μL of TBHP (Figure 1) were used. The reaction afforded 0.260 g of 2-amino-4,8-dibromo-1*H*-phenoxazin-1-one (**2**), and no further purification was performed.

**Figure 1:**
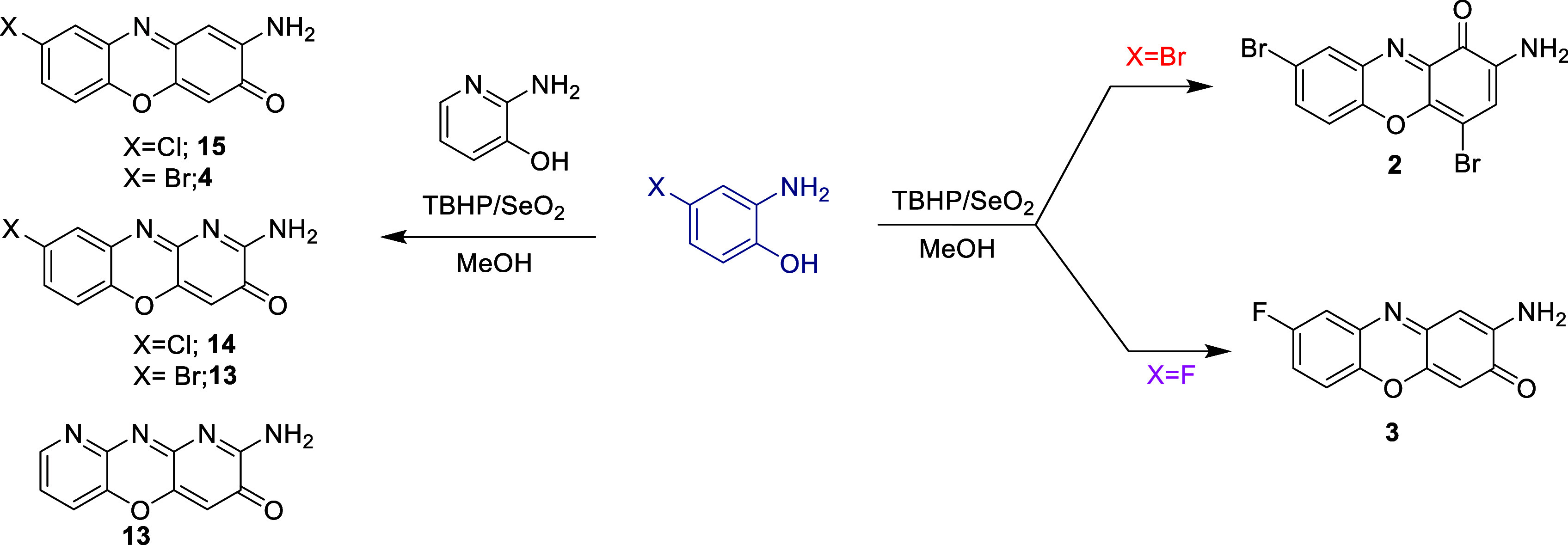
Scheme of the reaction for the oxidation with TBHP. In the right, the preparation of **2** (R1) and **3** (R2), in the left the preparation of **11**, **13**, **4**, and **15** (R3 and R4).

#### Synthesis Using 2-Amino-4-Fluorophenol as the Starting Material (Reaction 2)

A 0.193 g portion of 1.5 mM of 2-amino-4-fluorophenol (**3a**), 0.082 g of SeO_2_, and 250 μL of TBHP were used. After 30 min of stirring, 50 μL of TBHP (Figure 1) was added to afford 0.089 g of 2-amino-8-fluoro-3*H*-phenoxazin-3-one (**3**) with no further purification.

#### Synthesis Using 2-Aminopyrindin-3-ol and 2-Amino-4-Bromophenol as the Starting Material (Reaction 3)

1.8 mM of both starting materials, 0.345 g of 2-amino-4-bromophenol (**2a**), and 0.200 g of 2-aminopyrindin-3-ol (**4a**), 0.100 g of SeO_2_ and 350 μL of TBHP (87.5 μL every 15 min) were used (Figure 1). The mixture was stirred for 1 h 30 min to afford 0.600 g of crude mixture, which was purified by CPC using as solvent system *n*-hexane/ethyl acetate/methanol/water, 1/1/1/1, all v/v (HEMWat 0) in ascending mode, with a flow rate of 1 mL/min at 800 rpm for FCPC, with 106 test tubes collected every 1 min each, and 4 mL/min at 1250 rpm for mCPC, with 45 test tubes collected every minute each, and 5 mL/min at 2500 rpm for cCPC with 65 tubes collected every minute each to achieve 2-amino-8-bromo-3*H*-phenoxazin-3-one (**4**), 2-amino-8-bromo-3*H*-benzo­[*b*]­pyrido­[2,3-*e*]­[1,4]­oxazin-3-one (**14**), and 2-amino-3*H*-dipyrido­[3,2-b:2′,3′-e]­[1,4]­oxazin-3-one (**11**).

#### Synthesis Using 2-Aminopyrindin-3-ol and 2-Amino-4-Chlorophenol as the Starting Material (Reaction 4)

0.220 g of 2-aminopyrindin-3-ol (**4a**) (2.0 mM), 0.281 g of 2-amino-4-chlorophenol (**5a**) (1.9 mM), and 0.114 g of SeO_2_ (1.0 mM) were used; then 382 μM of TBHP was added (Figure 1). 0.298 g of crude mixture was obtained, which was purified by HPLC to obtain 2-amino-8-chloro-3*H*-phenoxazin-3-one (**15**), 2-amino-8-chloro-3*H*-benzo­[*b*]­pyrido­[2,3-*e*]­[1,4]­oxazin-3-one (**14**) and 2-amino-3*H*-dipyrido­[3,2-b:2′,3′-e]­[1,4]­oxazin-3-one (**11**). Solvent A was water +0.1% acetic acid, and solvent B was acetonitrile +0.1% acetic acid. The optimized gradient system was as follows: 0–5 min 30% B, 5–25 min to 100% B, and 25–30 min 100% B.

### General Methods for the Synthesis Using NaIO_3_ as an Oxidant

The starting phenol, dissolved in methanol or acetone, was added dropwise to an aqueous sodium iodate (NaIO_3_) solution and stirred for 10 min. A second portion of the same or a different phenol, dissolved in methanol or acetone, was then added dropwise. The mixture was stirred overnight at room temperature. The crude mixture was subsequently cooled in an ice bath for 20 min, vacuum filtered, and washed with cold water. The resulting precipitate, containing the desired APO derivatives, was purified by CPC or preparative HPLC.

#### Synthesis Using 2-Aminophenol and 2-Amino-4-Bromophenol as the Starting Material (Reaction 5)

2-Amino-4-bromophenol (**2a**) (0.7 mM, 0.129 g) in 10 mL of methanol was added to 0.7 mM sodium iodate (0.135 g) dissolved in 25 mL of water. After 10 min, 2-aminophenol (**1a**) (0.3 mM, 0.037 g) in 8 mL of methanol was added to the mixture (Figure 2). The reaction afforded 0.118 g of crude mixture, which was purified by CPC using as solvent system *n*-hexane/ethyl acetate/methanol/1,2-dimethoxyethane/water, 2/1/1/1/1, all v/v (HEMDmeWat +2) in ascending mode, with a flow rate of 0.7 mL/min at 800 rpm for FCPC, with 106 test tubes collected every 1.4 min each, and 4 mL/min at 1250 rpm for mCPC, with 140 test tubes collected every minute each, to yield 2-amino-3*H*-phenoxazin-3-one (**1**, **APO**), 2-amino-8-bromo-3*H*-phenoxazin-3-one (**4**), 2-amino-8-bromo-4-iodo-3*H*-phenoxazin-3-one (**5**) and 2-amino-4-iodo-3*H*-phenoxazin-3-one (**6**).

**Figure 2:**
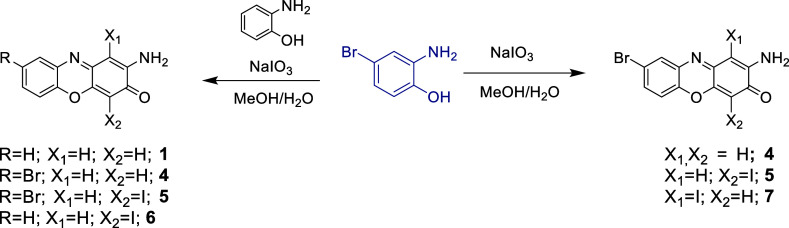
Scheme of the reaction for the oxidation with NaIO_3_. In the right the preparation of **4**, **5**, and **4-I** (R6), in the left the preparation of **1**, **4**, **5**, and **6** (R5).

#### Synthesis Using 2-Amino-4-Bromophenol as the Starting Material (Reaction 6)

A 0.140 g portion of 2-amino-4-bromophenol­(**2a**) (0.7 mM), in 8 mL of methanol was added to a solution of NaIO_3_ (1 mM, 0.197 g) in 30 mL of deionized water. After 10 min of stirring, 0.142 g of 2-amino-4-bromophenol (**2a**) (0.7 mM) in 8 mL of methanol was added to the mixture dropwise. 0.194 g of a crude reaction mixture was obtained (Figure 2). It was purified by CPC using as system solvent 1/1/1/1/1 (all v/v) of a mixture of *n*-hexane/ethyl acetate/methanol/1,2-dimethoxyethane/water (HEMDmeWat 0) in ascending mode with a flow rate of 1 mL/min at 800 rpm for FCPC, 30 test tubes were collected every 2 min each, and a flow rate of 5 mL/min at 1200 rpm for mCPC was used to collect 150 test tubes every minute each. The purifications isolated 2-amino-8-bromo-3*H*-phenoxazin-3-one (**4**) and 2-amino-8-bromo-4-iodo-3*H*-phenoxazin-3-one (**5**) and identified 2-amino-8-bromo-1-iodo-3*H*-phenoxazin-3-one (**4-1I**).

#### Synthesis Using 3-Amino-4-Hydroxybenzoic Acid as the Starting Material (Reaction 7)

A 0.153 g portion of 4-amino-3-hydroxybenzoic acid (**6a**) (1 mM) dissolved in 10 mL of methanol was added to NaIO_3_ (0.5 mM, 0.110 g in 25 mL of H_2_O). After stirring for 10 min, another portion of phenol (0.153 g in 10 mL of MeOH, 1 mM) was added. After 2 h, 0.237 g of the oxidant NaIO_3_ (1.2 mM, in 50 mL of H_2_O) was added to the reaction mixture (Figure 3). The reaction afforded 0.1538 g of the crude mixture. It was purified by CPC in two variants via HEMDmeWat 0 in descending mode, isolating 2-amino-3-oxo-3*H*-phenoxazine-7-carboxylic acid (**8**) by adding 0.1% of formic acid, whereas 2-amino-4-iodo-3-oxo-3*H*-phenoxazine-7-carboxylic acid was identified without the acid. The flow rate was 1 mL/min at 800 rpm in the FCPC collecting 35 test tubes every 2 min each.

**Figure 3:**
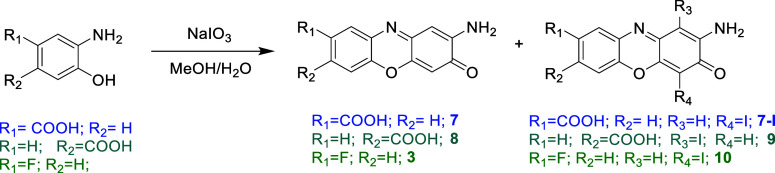
Scheme of the reaction for the oxidation with NaIO_3_ of **7** and **7-I** (R7), **8** and **9** (R8), and **3** and **10** (R9).

#### Synthesis Using 4-Amino-3-Hydroxybenzoic Acid as the Starting Material (Reaction 8)

A 0.154 g portion of 4-amino-3-hydroxybenzoic acid (**7a**) (1 mM) dissolved in 12 mL of methanol was added to 0.118 g of NaIO_3_ (0.6 mM) dissolved in 20 mL of deionized water with stirring. After 10 min, 0.154 g of 4-amino-3-hydroxybenzoic acid (1 mM) in 12 mL of methanol was added to the mixture. After 2 h, another portion of NaIO_3_ (1.2 mM, 0.237 g in 30 mL of H_2_O) was added to the mixture (Figure 3). A 0.376 g amount of the crude reaction mixture was obtained, which was purified via HPLC to afford 2-amino-3-oxo-3*H*-phenoxazine-7-carboxylic acid (**8**) and 2-amino-1-iodo-3-oxo-3*H*-phenoxazine-7-carboxylic acid (**9**). Solvent A was water +0.1% acetic acid, and solvent B was acetonitrile +0.1% acetic acid. The optimized gradient system was as follows: 0–5 min 30% B, 5–25 min to 100% B, and 25–30 min 100% B.

#### Synthesis Using 2-Amino-4-Fluorophenol as the Starting Material (Reaction 9)

A 0.100 g portion of 2-amino-4-fluorophenol (**3a**) (0.8 mM) dissolved in 12 mL of methanol was added to NaIO_3_ (1.6 mM, 0.318 g) dissolved in 20 mL of deionized water and stirred for 10 min. Then, another 0.100 g of 2-amino-4-fluorophenol (0.8 mM) dissolved in 12 mL of methanol was added to the mixture (Figure 3). The reaction afforded 0.221 g of the crude mixture, which was purified by CPC using HEMDmeWat 0 in ascending mode with a flow rate of 1 mL/min at 800 rpm for the FCPC, collecting 40 test tubes every 2 min each, and with a flow rate of 5 mL/min at 2500 rpm for the cCPC, which collects 45 test tubes every minute each. In both experiments, the purification afforded 2-amino-8-fluoro-3*H*-phenoxazin-3-one (**3**) and 2-amino-8-fluoro-4-iodo-3*H*-phenoxazin-3-one (**10**).

#### Synthesis Using 2-Aminopyrindin-3-ol as the Starting Material (Reaction 10)

A 0.330 g portion of 2-aminopyrindin-3-ol (**4a**) (3 mM) dissolved in 15 mL of methanol was added to NaIO_3_ (1.7 mM, 0.337 g) dissolved in 50 mL of deionized water. After 10 min stirring, another portion of 2-aminopyrindin-3-ol (3 mM, 0.330 g in 15 mL MeOH) was added. In this case, after 2 h, 0.722 g of NaIO_3_ (3.6 mM) in 60 mL of deionized water was added (Figure 4) to afford 0.522 g of 2-amino-3*H*-dipyrido­[3,2-b:2′,3′-e]­[1,4]­oxazin-3-one (**11**) after the workup.

**Figure 4:**
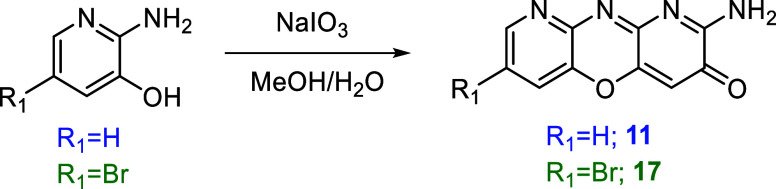
Scheme of the reaction for the oxidation of NaIO_3_ of the nitrogen-containing derivatives **11** (R10) and **17** (R11).

#### Synthesis Using 2-Amino-6-Bromopyrindin-3-ol as the Starting Material (Reaction 11)

A 0.124 g portion of 2-amino-6-bromopyrin-3-ol (**8a**) (0.7 mM) dissolved in 12 mL of methanol was added to the oxidant NaIO_3_ (0.174 g, 1 mM) dissolved in 20 mL of deionized water. After 10 min, another 0.124 g of 2-amino-6-bromopyrindin-3-ol (0.7 mM) dissolved in 12 mL of methanol was added. In this case, after 2 h, 0.312 g of NaIO_3_ (1.5 mM) dissolved in 30 mL of deionized water was added (Figure 4) to the mixture to afford 0.238 g of 2-Amino-7-bromo-3*H*-dipyrido­[3,2-b:2′,3′- e]­[1,4]­oxazin-3-one (**12**) after the workup.

Yields, synthesis, and separation conditions for all reactions are summarized in Tables 1 and 2.

**Table 1:**
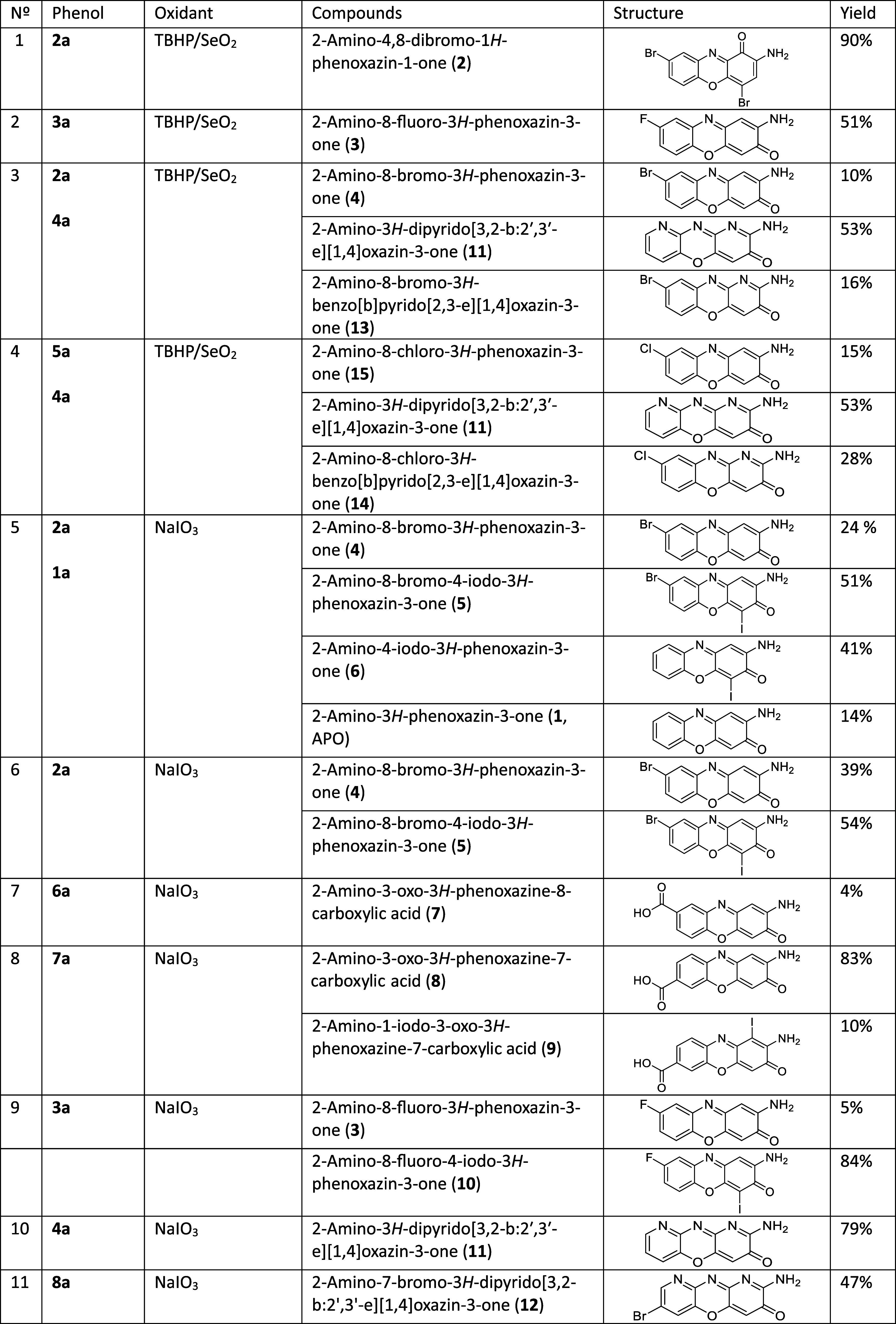
Synthesis Conditions and Yields of Derivatives^a^

a All TBHP/SeO_2_ reactions last 1h and NaIO_3_ last overnight. All reactions were done in room temperature.

**Table 2:** CPC Conditions for Purification of R3, R5, R6, R7, and R9

reaction	solvent system/mode	flow/rpm	*S*_f_ (%)	*S*_D_ (%)	recovery (%)	purity (%)	weight (mg)	compounds	device
R3	HEMWat 0 (*n*-Hexane/Ethyl acetate/methanol/water; 5/5/5/5, v/v) asc	1 mL/min at 800 rpm	68	62	75	85	0.4	**4**	FCPC
						92	0.4	**13**	
						91	5.1	**11**	
		5 mL/min at 1200 rpm	74	45	86	81	1.4	**4**	mCPC
						94	2.0	**13**	
						90	25.0	**1**	
R5	HEMDmeWat +2 (*n*-hexane/ethyl acetate/methanol/1,2- dimethoxy ethane/water; 2/1/1/1/1, v/v)asc	0.7 mL/min at 800 rpm	68	71	99	90	3.1	**1**	FCPC
						87	1.1	**4**	
						90	3.4	**5**	
						99	2.5	**6**	
		4 mL/min at 1200 rpm	74	61	90	99	9.8	**1**	mCPC
						92	1.4	**4**	
						90	5.4	**5**	
						93	5.1	**6**	
R6	HEMDmeWat 0 (*n*-Hexane/Ethyl Acetate/Methanol/1,2- Dimethoxy ethane/Water; 1/1/1/1/1, v/v) asc	1 mL/min at 800 rpm	63	46	99	92	2.1	**4**	FCPC
						70	2.8	**5**	
								**4-1I**	
		5 mL/min at 1300 rpm	73	50	98	96	5.0	**4**	mCPC
						97	7.1	**5**	
						70		**4-1I**	
R7	HEMDmeWat 0 (*n*-Hexane/Ethyl Acetate/Methanol/1,2- Dimethoxy ethane/Water; 1/1/1/1/1, v/v) asc	1 mL/min at 800 rpm	63	17	95			**7**	FCPC
						96	1.1	**7-I**	
	HEMDmeWat 0 + 0.1% FA dsc		66	10	99	97	3.1	**7**	
								**7-I**	
R9	HEMDmeWat 0 (*n*-Hexane/Ethyl Acetate/Methanol/1,2- Dimethoxy ethane/Water; 1/1/1/1/1, v/v) asc	1 mL/min at 800 rpm	68	60	97	94	1.2	**3**	FCPC
						90	2.3	**10**	
		5 mL/min at 2500 rpm	67	65	99	99	1.6	**3**	cCPC
						97	6.9	**10**	

### Wheat Coleoptile Bioassay

This procedure was performed according to the method previously optimized by our research group and described by Rial et al.^36^ Compounds were predissolved in DMSO (5 μL of DMSO/mL of buffer) and then diluted to final concentrations of 1000, 300, 100, 30, and 10 μM in a phosphate/citrate buffer (pH 5.6) with 2% sucrose. The commercial herbicide Logran Extra 60 (Syngenta, Switzerland) served as a positive control at the same concentrations. A solution of 0.1% DMSO in phosphate/citrate buffer was used as a negative control. Five coleoptiles and 2 mL of solution were placed in each test tube, and the tubes were rotated at 6 rpm in a roller tube apparatus for 24 h at 25 °C in the dark. Triplicates were performed for each dilution.

The results are expressed as the percent difference from the control: positive values indicate stimulation of the elongation of the coleoptiles, negative values indicate inhibition, and zero represents the control. Data were statistically analyzed using Welch’s test.

### Phytotoxic Activity against Weed Species

In Petri dishes, 20 seeds per dish (four replicates/concentration) were treated with test compounds at concentrations of 10, 30, 100, 300, and 1000 μM in 1.0 mL of 10 mM MES buffer (10^–2^ M 2-[*N*-morpholino] ethanesulfonic acid (MES) (pH 6.0). A buffer-only solution served as a negative control, while Stomp Aqua’s active ingredient (pendimethalin, P) was the positive control. Dishes were kept in darkness at 25 °C in a climate-controlled growth chamber for 7 days for *L. rigidum*, *P. oleracea*, and *P. lanceolata* and then frozen for 24 h. Shoot and root lengths were measured using a Fitomed digitizing table. Statistical analysis utilized Welch’s test, with significance levels set at 0.01 and 0.05.

### Calculation of IC_50_ and Clog *P* Values

IC_50_ values were determined by fitting the activity data to a sigmoidal dose-response model using GraphPad Prism 5.0 software. Lipophilicity is expressed by the Clog *P* calculation method as implemented in ChemBioDraw Ultra 21.0 software.

### CPC Experiments

The chosen solvent systems were prepared by a separation funnel. The layers were separated and degassed in an ultrasonic bath for 3–5 min. The stationary phase was loaded into the rotor by using twice the volume of the CPC used (FCPC: 55 mL; mCPC: 250 mL; cCPC: 1000 mL). The system was equilibrated using the mobile phase at a flow rate and rpm set for the purification method. The stability of the system was determined by calculating the stationary phase retention volume ratio (*S*_f_) when equilibrium was reached.

The samples were dissolved in an equal mixture of each phase of the solvent system selected. For FCPC, the maximum amount to dissolve was 25 mg in 4 mL; for mCPC, 60 mg in 8 mL; and for cCPC, 30 mg in 8 mL. We consider these amounts 100% sample solubility. The mixture was then centrifuged for 5 min at 2500 rpm and filtered through a polytetrafluorethylene filter (Φ_pore_ = 0.45 μm). The fractions were analyzed by TLC and HPLC-DAD.

#### Solvent System Selection for Centrifugal Partition Chromatography

The system solvent most suitable for our mixtures was HEMWat,^37^ which is composed of *n*-hexane/ethyl acetate/methanol/water and comprises 16 different systems split in polarities. Different other common solvent systems, such as CMat (chloroform/methanol/water), HEMWat amplified (adding 1-butanol), a mix of MTBE/BuOH/ACN/water, and THF/DMSO/water, and some water-free systems, such as different mixes of ACN/Hex and MeOH/Hex, were also tested. However, neither of them showed good theoretical values nor a desirable dissolution of the sample.

1 $$K=\frac{A_{SP}}{A_{MP}}\alpha =\frac{K_{i}}{K_{j}}$$

System selection prioritized stability: those forming an interface within 30 s were chosen. The shake-flask method to calculate K^38^ (partition coefficient) and α (separation factor) (eq 1) was used. This entailed dissolving ∼1 mg of each mixture in the test system (1 mL per layer), shaking, drying 500 μL of each layer, then redissolving in methanol for HPLC/DAD analysis. Acceptable coefficients were 0.125 < *K* < 8 (or 0.4 < *K* < 2.5 for enhanced separation) and α > 1.5. The poor solubility of most reaction products and APO complicated system selection, requiring solvent additives such as 1,2-dimethoxyethane (DME), dimethyl sulfoxide (DMSO), or tetrahydrofuran (THF).

## Results and Discussion

### Synthesis

The synthesis of the target aminophenoxazinone derivatives was approached using two distinct oxidative cyclocondensation strategies, sodium iodate (NaIO_3_) and *tert*-butyl hydroperoxide/selenium oxide (TBHP/SeO_2_), with their efficacy being highly dependent on the starting substrates (halogenated, acidic, or nitrogen-containing).

Our initial strategy employed was sodium iodate (NaIO_3_). This oxidant was effective in promoting the cyclocondensation for all three substrate classes, yielding halogenated derivatives **3** and **4**, and acidic derivatives **7** and **8**. However, this method presented a consistent and significant drawback: the formation of new iodinated byproducts **5**, **6, 9**, **10**. This side-reaction was not trivial; in the synthesis of the fluorinated derivative **3**, the corresponding iodinated byproduct **10** was the major component, reaching an 84% yield. This indicates a strong preference for iodination, particularly at C-4. Notably, this iodination issue was not observed during the synthesis of the nitrogen-containing heterocycles **11** and **12** using NaIO_3_, suggesting a different reactivity profile for these substrates. To prevent iodination, a second strategy using TBHP/SeO_2_ was explored. This approach successfully avoided iodination but introduced new challenges related to regioselectivity and reaction mechanism. When applied to 2-amino-4-bromophenol (**2a**), it did not yield the kinetic product **4**, but instead produced the structurally distinct derivative **2** (brominated at C-4 and C-8). This outcome is consistent with the thermodynamic mechanism described by Granda et al.,^39^ where the oxidant itself dictates a shift from a β-position attack (kinetic) to a α-position attack (thermodynamic). This mechanistic shift, however, was substrate-dependent. When 2-amino-4-fluorophenol (**3a**) was used, the TBHP reaction still followed the kinetic regime, yielding **10** (without iodine, of course). Furthermore, this oxidant system proved unsuitable for the acidic derivatives, as its increased oxidative character “did not work properly” and led to the formation of many byproducts.

Finally, TBHP/SeO_2_ was used in mixed-component reactions (3 and 4) to synthesize nitrogen-fused targets **13** and **14**. While successful, these reactions were not clean, also producing self-reaction byproducts of the starting materials (e.g., **11**, **4**, and **15**).

In summary, our findings demonstrate that the choice of oxidants is a critical parameter that not only governs side-reactions (iodination) but also fundamentally controls the reaction’s regioselectivity (kinetic vs thermodynamic), with the final outcome being highly dependent on the specific phenol substrate employed.

### Purification

Our findings confirm that CPC was an indispensable purification strategy, enabling us to overcome the analyte degradation and irreversible adsorption issues commonly associated with solid stationary phases. As a result, we achieved near 100% sample recovery in most trials with final compound purities reaching 90–99% (Table 2). The critical challenge in method development was not the partition coefficients (*K*_D_) themselves but rather the poor sample solubility in standard biphasic solvent systems. For instance, while the standard HEMWat system showed promising theoretical *K*_D_ values, its practical application for the mixtures from Reactions 5 and 6 was unfeasible, yielding sample solubilities of only 27% and 13%, respectively. The solution was the optimization of the solvent system with additives. The incorporation of 1,2-dimethoxyethane (DME) was essential, improving solubility (up to 71% for Reaction 5) and permitting successful purification and scale-up. In contrast, the mixture from Reaction 3 did not present this solubility issue (62% in System 4) and was effectively purified without requiring additives. The most significant challenge was presented by the acidic derivatives (Reaction 7). Solubility in the standard HEMWat system was negligible (<3%). Even with the addition of DME, solubility remained poor (17%), and anomalous “bleeding” of compound **7-I** was observed. Only through the acidification of the system (adding 0.1% formic acid) was it possible to modulate the partitioning and elute compound **7** with high purity (97%), although solubility remained extremely low (10%). Attempts to improve solubility using a base (TEA) failed. In all successful cases, the method was effectively transferred from the analytical scale (FCPC) to the preparative scale (mCPC/cCPC).

**Figure 5:**
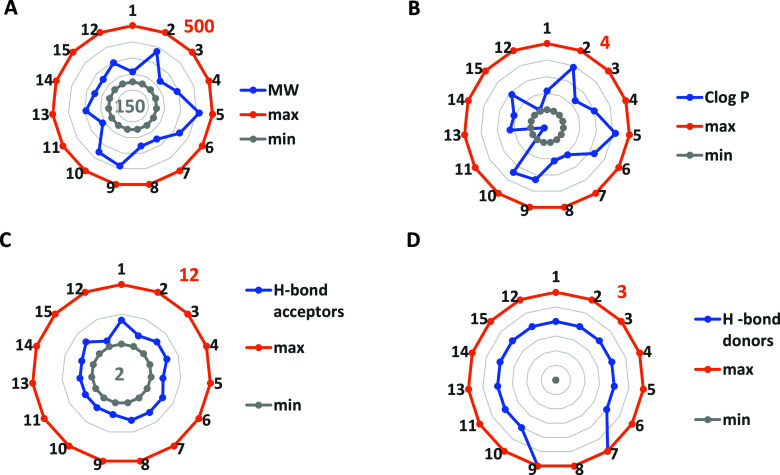
Graphs providing molecular descriptors compounds: (A) molecular weight; (B) Clog *P*, partition coefficient; (C) number of hydrogen bond acceptors; and (D) number of hydrogen bond donors. Gray and orange lines, respectively, indicate the minimum (min) and maximum (max) values according to Tice^36^.

This scale-up was not only viable but generally improved the results, showing higher stationary phase retention (*S*_f_) which leads to a better resolution, likely due to the increased rotational speeds and rotor volumes. This work underscores that while theoretical coefficients are crucial for CPC viability, the properties of the sample matrix (such as solubility), and the experimental optimization of the solvent system are the factors that ultimately dictate purification success.

### Characterization

The structural elucidation of the synthesized derivatives was confirmed by NMR and HRMS (NMR values are found in Tables 3 and 4, and NMR spectra and MS values are in the Supporting Information), with distinctive spectroscopic signatures observed for each compound class. In the halogenated derivatives, NMR was crucial for confirming substituent positions. The incorporation of iodine, as in the new compounds **5**, **6** and **10**, was evidenced by a significant shielding effect in the ^13^C NMR spectrum. Specifically, the carbon signal in C-4 shifted dramatically from 103.4 to 81 ppm. The same effect was observed for acidic iodinated derivatives, such as compound **11**, where substitution at C-1 shielded this signal from 98.7 to 73.9 ppm. Fluorinated derivatives (**3** and **10**) displayed expected multiplicity patterns due to coupling with the active ^19^F nucleus. This resulted in ^1^H–^19^F couplings in the aromatic protons and ^13^C–^19^F couplings in the carbons, with coupling constants as high as 240 Hz for carbons directly bonded to fluorine. Finally, the nitrogen-containing heterocycles (**11**, **12**, **13**, and **14**) showed an entirely different set of changes. In the ^1^H NMR spectrum, the presence of the additional nitrogen atom induced a general deshielding of the protons relative to parent scaffold **1** (APO). Specifically, the H-4 proton was deshielded from 6.37 ppm in **1** to 6.56, 6.53, and 6.67 ppm for **11**, **13**, and **12**, respectively. Similarly, H-6 shifted from 7.51 to 7.95 ppm in **11** and **13**, and up to 8.60 ppm in **12**. H-8 also deshielded from 7.40 ppm to 8.54 and 8.31 ppm in **11** and **12**, respectively. In ^13^C NMR, the evidence was unequivocal: the expected carbon signal at position 1 (around 98 ppm) disappeared completely, confirming its replacement by a nitrogen atom as proposed in the structures. The presence of a carboxylic acid was evidenced by the absence of the signal corresponding to H-8 in **7**, or H-7 in **8** and **9**. This was corroborated with the presence of a signal corresponding to carboxylic carbon at 167.1, 166.1, and 166.5 ppm for compounds **7**, **8**, and **9**, respectively. The exact masses for all compounds were confirmed by HRMS.

**Table 3:** ^1^H NMR Data^a^

H	1	2	3	4	5	6	7	8	9	10	11	13	14	15	12
1	6.37; s		6.35; s	6.35; s	6.35; s	6.38; s	6.37; s	6.40; s		6.37; s				6.35; s	
3		6.41; s													
4	6.37; s		6.36; s	6.37; s			6.41; s	6.38; s	6.41; s		6.56; s	6.53; s	6.06; s	6.39; s	6.67; s
6	7.51; dd *J =* 8.2; 1.3 Hz	7.51; d *J* = 8.8 Hz	7.56; dd *J =* 8.9; 5.0 Hz	7.47; d *J =* 8.9 Hz	7.55; d *J* = 8.8 Hz	7.60; dd *J =* 8.3; 1.3 Hz	7.55; d *J =* 8.5 Hz	7.91; d *J =* 1.5 Hz	7.94; d *J =* 1.5 Hz	7.63; dd *J =* 9.0; 4.3 Hz	7.95; d *J* = 8.2 Hz	7.94; d *J =* 9 Hz	6.93; d *J =* 8.8 Hz	7.55; d *J =* 8.6 Hz	8.60; d *J =* 2.1 Hz
7	7.47; ddd *J =* 8.0, 1.5 Hz	7.65; dd *J =* 8.8; 2.2 Hz	7.33; ddd *J =* 9.0; 8.4; 3.1 Hz	7.58; dd *J =* 8.5; 2.4 Hz	7.63; dd *J* = 8.8; 2.2 Hz	7.53; ddd *J =* 7.4; 8.0; 1.5 Hz	7.97; dd *J =* 8.5; 2.0 Hz			7.37; ddd *J =* 8.5; 2.7 Hz	7.45; dd *J =* 8.8; 3.8 Hz	7.60; dd *J =* 9.0; 2.5 Hz	7.14; dd *J =* 2.3; 8.4 Hz	7.48; dd *J =* 2.6; 9.6 Hz	
8	7.40; ddd *J =* 7.9; 1.5 Hz					7.45; ddd *J =* 7.4; 8.0; 1.5 Hz		7.88; dd *J =* 1.5; 7.9 Hz	7.92; dd *J =* 8.3; 1.5 Hz		8.54; d *J* = 8.8 Hz				8.31; d *J =* 2.1 Hz
9	7.71; dd *J =* 7.9; 1.5 Hz	7.95; d *J =* 2.2 Hz	7.53; dd *J =* 9.3; 3.0 Hz	7.87; d *J =* 2.4 Hz	7.92; dd *J* = 2.2 Hz	7.77; dd *J =* 8.0; 1.4 Hz	8.18; d *J =* 2.1 Hz	7.75; dd *J =* 0.9; 8.0 Hz	7.84; d *J =* 8.2 Hz	7.58; dd *J =* 9.3; 2.7 Hz		7.83; d *J =* 2.5 Hz	7.20; d *J =* 2.6 Hz	7.75; d *J =* 2.6 Hz	
NH_2_	6.80; bs	7.17; bs	7.0; bs	6.97; bs	7.10; bs	6.92; bs	6.91; bs	7.01; bs	7.00; bs	7.1; bs	8.28; bs			6.99; bs	

a Measured in DMSO-*d*_6_ in 500 MHz.

**Table 4:**
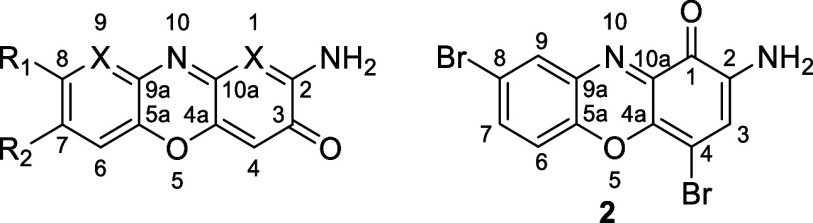
^13^C NMR Data^a^

C	1	2	3	4	5	6	7	8	9	10	11	13	14	15	12
1	98.3	177.9	97.9	98.1	98.1	98.4	98.3	98.7	73.9	98.0					
2	148.9	149.9	149.0	149.0	149.8	149.8	149.0	150.1	150.4	150.0	158.1	158.4	158.3	158.4	156.6
3	180.2	103.2	180.1	180.2	176.2	176.1	180.4	180.7	177.3	176.1	176.3	176.0	175.9	176.0	175.8
4	103.4	94.8	103.4	103.7	81.2	80.9	105.3	104.2	103.3	80.8	105.3	105.7	102.5	105.7	108.5
4a	148.2	146.5	148.9	148.7	148.3	147.5	148.7	149.6	150.1	148.2	151.0	151.6	151.5	151.6	147.7
5a	141.9	141.9	138.5 *J*_*C–F*_ *=* 2.2 Hz	141.2	141.6	142.3	144.6	141.9	142.1	138.9	138.6	141.7	141.7	141.7	138.9
6	115.9	118.3	117.3 *J*_*C–F*_ *=* 9.7 Hz	117.9	118.1	116.1	116.2	128.2	126.3	117.5 *J*_*C–F*_ *=* 9.7 Hz	124.1	118.5	118.6	118.5	146.9
7	128.8	132.3	115.7 *J*_*C–F*_ *=* 24.6 Hz	130.8	131.2	129.2	129.2	131.6	131.6	116.1 *J*_*C–F*_ *=* 24.7 Hz	123.5	131.3	131.3	131.2	118.0
8	125.3	117.7	158.7 *J*_*C–F*_ *=* 240 Hz	116.5	131.8	125.8	127.6	126.2	116.8	158.9 *J*_*C–F*_ *=* 242.0 Hz	146.8	117.3	117.5	117.3	126.6
9	127.9	130.5	112.0 *J*_*C–F*_ *=* 24.6 Hz	129.6	129.5	127.8	128.9	117.1	128.7	112.8 *J*_*C–F*_ *=* 23.5 Hz		130.4	131.4	130.4	
9a	133.7	134.9	134.5 *J*_*C–F*_ *=* 13.6 Hz	135.1	135.3	134.0	133.4	137.1	136.9	134.8 *J*_*C–F*_ *=* 13.0 Hz	145.8	135.9	135.8	136.0	142.8
10a	147.3	145.3	147.9	147.8	146.3	145.8	147.6	148.6	147.3	146.3	153.0	151.3	152.0	151.3	148.7
COOH							167.1	166.1	166.5						

a Measured in DMSO-*d*_6_ in 150 MHz.

**Figure 6:**
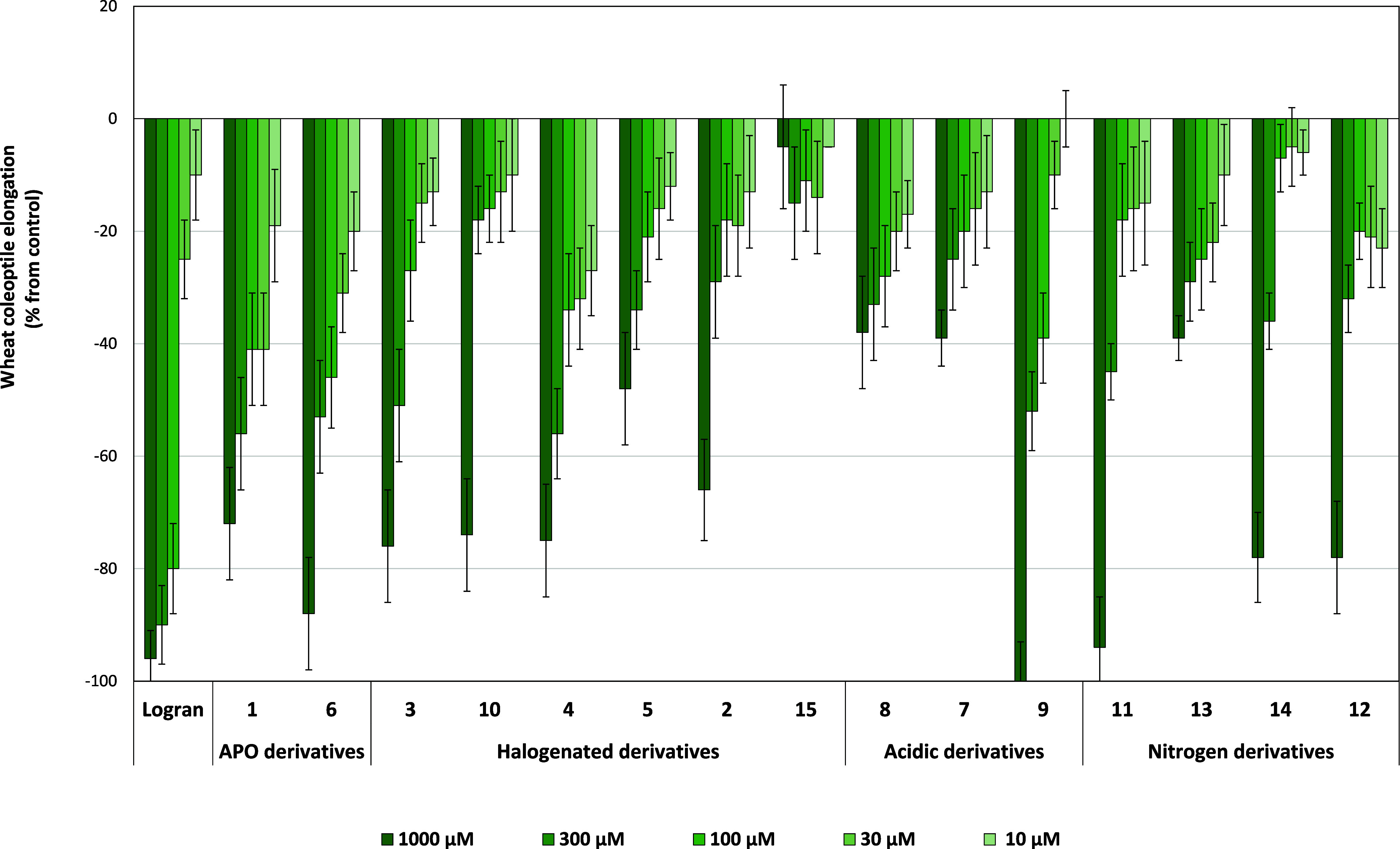
Effects of the synthetic compounds and the herbicide Logran on the elongation of wheat coleoptiles. The values are expressed as the percentage difference from the control. Each bar represents the mean ± standard deviation.

### Bioassay

Agrochemical efficacy depends on availability, stability, and physicochemical properties. Tice’s optimized parameters^40^ (MW 145–500 uma, Clog *P* ≤ 4, H-bond donors 1–2, H-bond acceptors ≤3, rotatable bonds ≤12) were met by all compounds (Figure 5). In brief, all compounds fulfill the parameters required for the development of an agrochemical according to these criteria except compound **11** for Clog *P*. Therefore, the phytotoxicity of these compounds was subsequently studied.

### Wheat Etiolated Coleoptiles Bioassay

The wheat coleoptile elongation assay (Figure 6) offers a rapid and sensitive preliminary assessment of the compound phytotoxicity. Most compounds inhibited growth over 65% at high concentrations and it allowed us to identify the most promising functional groups. Derivatives **5**, **7**, **8**, **13**, and **15** showed reduced activity compared to APO and were subsequently excluded from further bioassays. Conversely, derivatives with iodine at C-1 or C-4 (**6**, **9** or **10**), and fluorine at C-8 (**3** or **10**), displayed promising activities, occasionally reaching 100% inhibition.

### Phytotoxicity Activity against Weeds

Compounds that achieved similar or better APO (**1**) inhibition of coleoptile (72 at 1000 μM) were tested. In this weed-specific bioassay we evaluated germination, root and shoot growth of the chosen seed. *L. rigidum*, *P. oleracea*, and *P. lanceolata* are typical weeds that affect crops such as olives, orchards, and wheat.^19^

For both *P. oleracea* (Figure 7) and L. rigidum (Figure 8), germination was not inhibited by any of the tested compounds (data not shown). The only remarkable effect was observed for compound **9** in *L. rigidum* *,* which reached 50% at the highest concentration.

**Figure 7:**
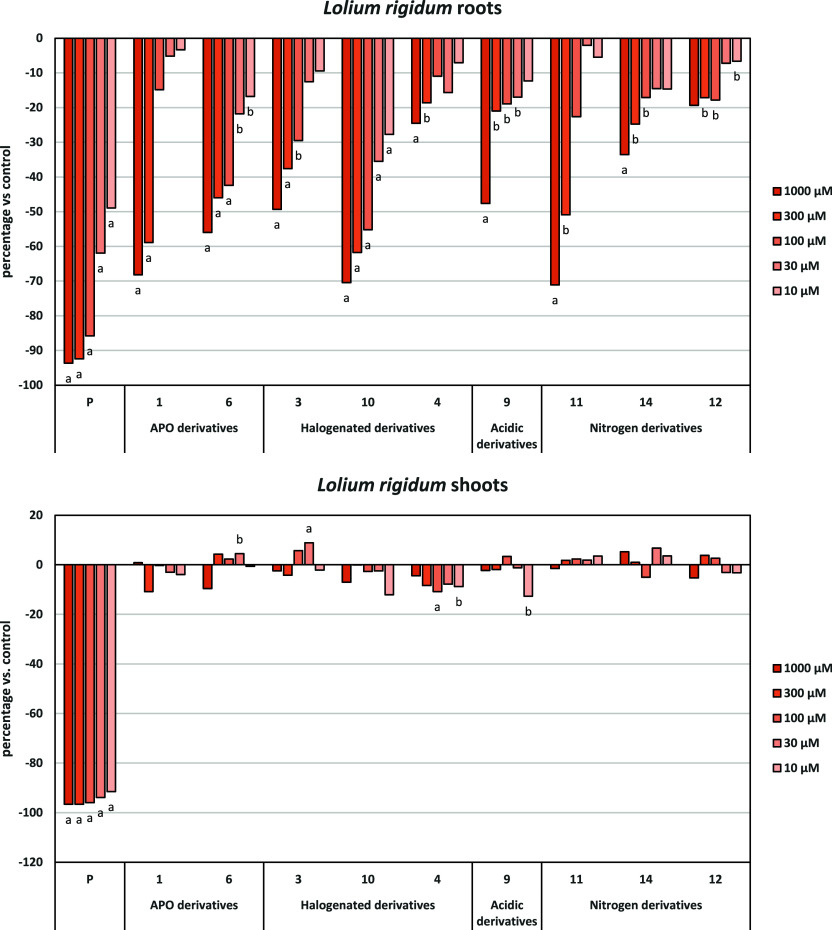
Effects of the synthetic compounds and the active ingredient of the herbicide Stomp Aqua (pendimethalin, P) on the root and shoot of length of *P. oleracea*. Data expressed as the percentage of difference from the control. Significance levels p < 0.01 (a) or 0.01 < p < 0.05 (b).

**Figure 8:**
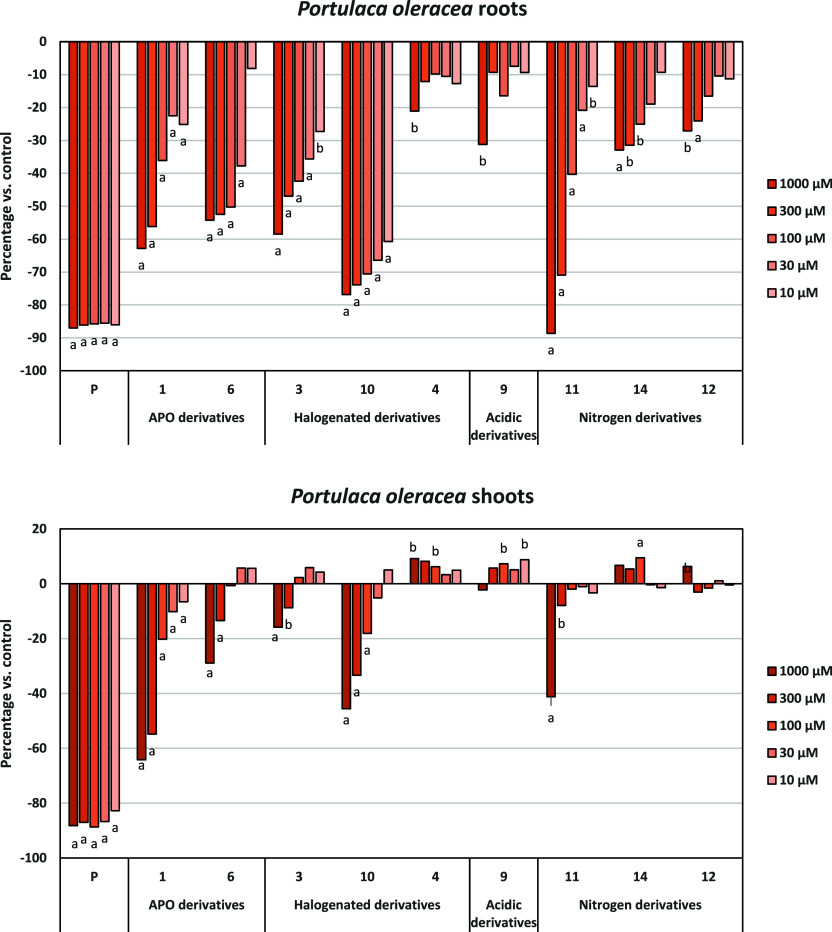
Effects of the synthetic compounds and the active ingredient of the herbicide Stomp Aqua (pendimethalin, P) on the root and shoot of length of *L. rigidum*. Data expressed as the percentage of difference from the control. Significance levels p < 0.01 (a) or 0.01 < p < 0.05 (b).

Regarding root growth, compounds **10** and **11** demonstrated significantly higher inhibitory activity, reaching 70% in *L. rigidum* and, 80% and 90%, respectively in *P. oleracea*. Compound **10**, bearing a fluorine atom at C-8 and an iodine atom at C-4, demonstrated a high inhibitory activity against *P. oleracea*, with the best profile of tested compounds, including APO (**1**). It maintained 61% inhibition even at the lowest tested concentration (10 μM), highlighting its potent phytotoxicity, also in *L. rigidum*. This remarkable activity underscores the critical role of the iodine atom in augmenting the molecule’s inhibitory capacity. Specifically, **10** exhibited a 20% higher inhibition compared to its iodine-free analogue, **3**, and the starting product, APO (**1**). This superior performance is further corroborated by the calculated IC_50_ values (Table 5): **10** demonstrated an IC_50_ of 4.5 μM, significantly lower than the 332.5 μM observed for APO (**1**). Compounds **6** and **11** also showed similar or better IC_50_ values than **1**, especially in *P. olearacea* [IC_50_: 332.5 (**1**); 71.8 (**6**), 143.1 (**11**)]. Concerning shoot growth inhibition, we call it *L. rigidum* shoot growth was not inhibited by any of the tested compounds. In *P. oleracea*, compounds **10** and **11** again exhibited significant inhibitory activity.

**Table 5:** IC_50_ Values of the Phytotoxicity Bioassay on Weeds of Compounds Tested

	L. rigidum roots		P. oleracea roots		P. lanceolata roots	
	IC_50_ (μM)	*R* ^2^	IC_50_ (μM)	*R* ^2^	IC_50_ (μM)	*R* ^2^
**P**	11.7	0.98	0.6	0.98	7.5	0.99
**1**	300.1	0.97	332.5	0.93	107.0	0.98
**6**	422.1	0.99	71.8	0.82		
**3**	897.5	0.99	673.5	0.86	196.2	0.98
**10**	91.1	0.88	4.5	0.91	164.0	0.96
**4**					523.0	0.99
**9**					223.0	0.99
**11**	325.0	0.99	143.1	0.99		
**14**						
**12**						

Equation 1. Theoretical coefficients *K*, partition coefficient, and α, separation factor. Asp and Amp are the recorded peak area of the target compound in the stationary (SP) and mobile phase (MP). *K*_*i*_ and *K*_*j*_ are the partition coefficients of the compounds *i* and *j*, being *K*_i_ > *K*j.

Regarding *P. lanceolata* (Figure 9), significant inhibition of germination, root, and shoot growth by halogenated derivatives was observed. In germination, even nitrogen derivatives with halogens at C-7 and/or C-8 (**14** and **12**) exhibited inhibition reaching 83% at the highest concentration tested (compound **12**, with Br at C-8 and N at positions 1 and 9). This activity decreased in root and shoot growth, where compounds **3**, **10**, and **4** showed inhibition values of 80% for both the root and shoot. This indicates that the presence of highly electronegative substituents at C-8, such as fluorine in **3** and **10**, and bromine in **4**, is crucial for the inhibition of *P. lanceolata* seed growth. In contrast to the previously discussed seeds (*P. oleracea* and *L. rigidum*), where an iodine atom at C-1 was essential for inhibition, it was found that although it does not induce a significant difference, compound **10** maintained inhibitory activity longer with dilution. It should be noted that compound **9** showed root inhibition reaching 85% at the highest concentration tested, but this effect rapidly diminished to 18% at 100 μM. The nitrogenated and acid derivatives show more variable activity, suggesting that specific modifications within these groups could be crucial in determining their phytotoxic potency.

**Figure 9:**
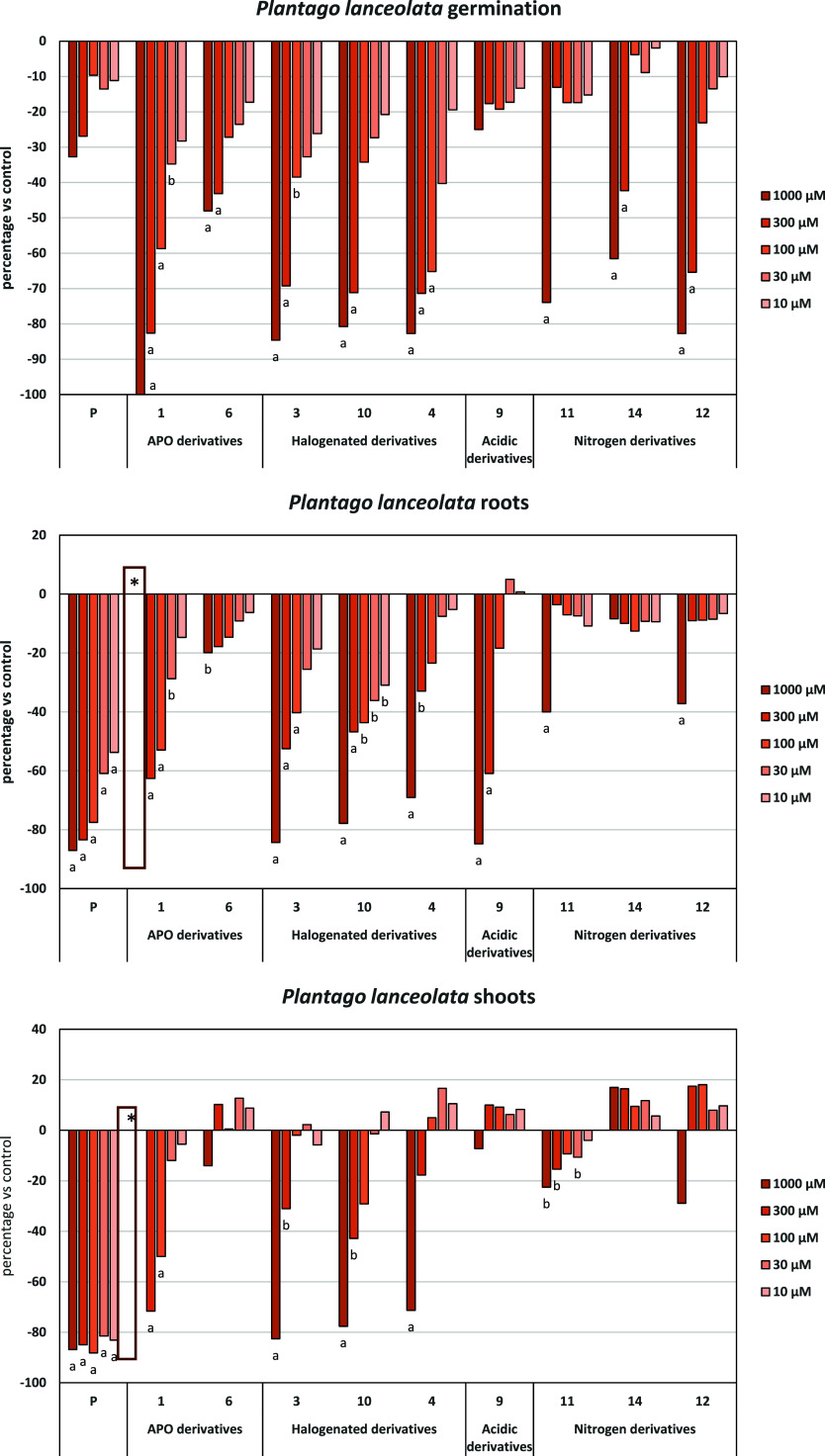
Effects of the synthetic compounds and the active ingredient of the herbicide Stomp Aqua (pendimethalin, P) on the germination, root and shoot of length of *P. lanceolata*. Data expressed as the percentage of difference from the control. Significance levels *p* < 0.01 (a) or 0.01 < *p* < 0.05 (b). *data not shown.

Based on extensive bioassay data, clear Structure–Activity Relationships (SAR) were established for the synthesized library, as summarized in Figure 11. The introduction of electronegative atoms at the position C-8 was determined to enhance general phytotoxic activity. Similarly, the presence of an iodine atom at the C-4 position was found to independently enhance the potency. Furthermore, the specific substitution of carbon atoms with nitrogen at positions 1 or 9 resulted in improved activity in *P. lanceolata*. Most significantly, the synergistic combination of fluorine at C-8 and iodine at C-4 led to enhanced activity across all species tested, providing a structural rationale for the superior broad-spectrum performance. The halogenated derivative **10** (Figure 10) appears to exhibit a more consistent and intense phytotoxic activity on the root growth of the three weed species evaluated, particularly at the highest dilution concentration tested and exhibits the lowest IC_50_ value for roots (Table 5), which means that it still maintains considerable activity, showing in *P. olearacea* and *L. rigidum* better activity profiles than **1**. Concurrently, improved physicochemical properties and enhanced solubility were observed, which facilitates improved product handling, its isolation and separation, and its prospective application against weeds.

**Figure 10:**
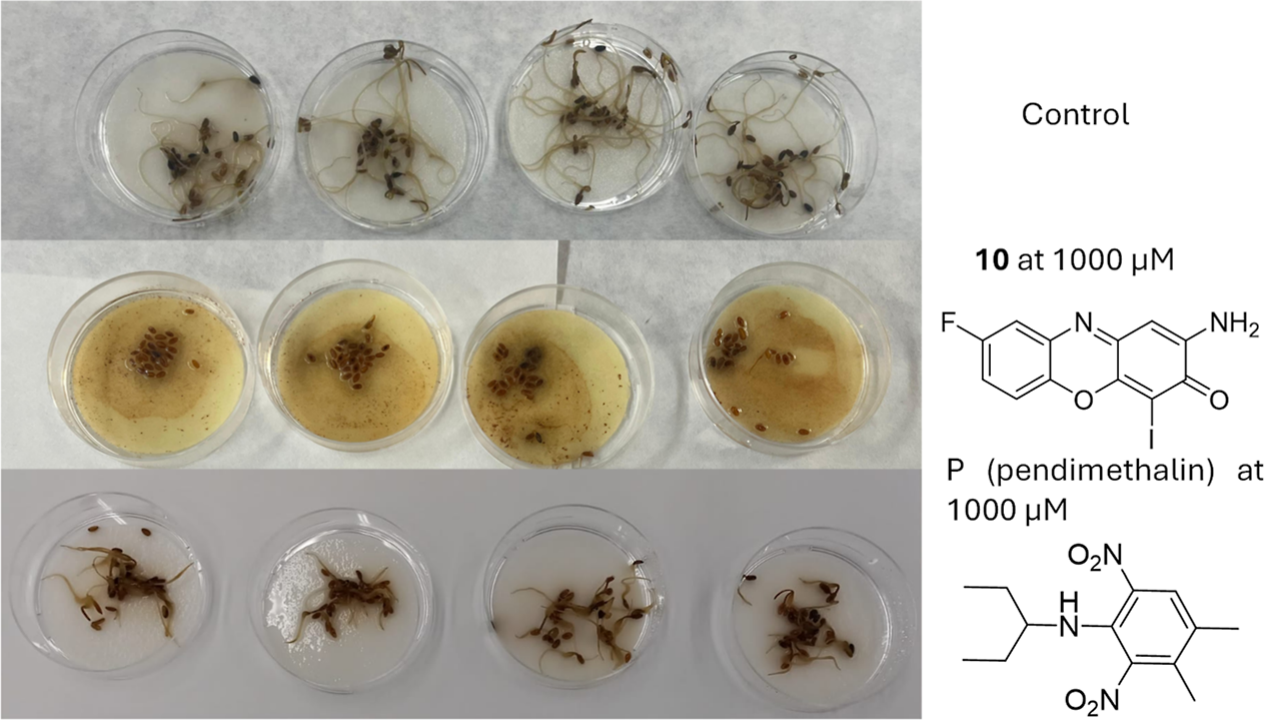
Effects on the seeds of *P. lanceolata* of control, derivative **10**, and pendimethalin at 1000 μM.

**Figure 11:**
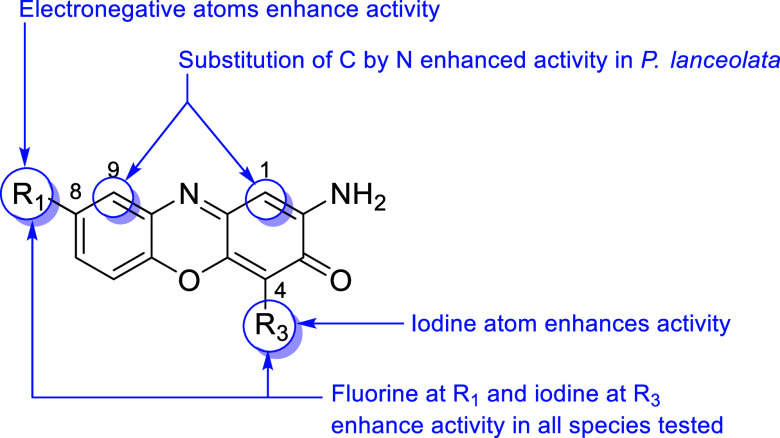
Structure–activity relationship regarding the inhibitory activity in weed.

While the natural APO scaffold offers a promising starting point as a result of its evolutionary selected bioactivity, the introduction of halogen atoms aims to optimize metabolic stability and lipophilicity. Although halogenation is a standard strategy in agrochemistry to enhance half-life, future studies on soil persistence and ecotoxicity will be essential to confirm that these derivatives retain an environmentally favorable profile compared to synthetic standards.

In conclusion, a focused library of aminophenoxazinone (APO) derivatives was synthesized via oxidative cyclocondensation and successfully purified by CPC. This technique proved essential for overcoming the strong irreversible adsorption observed on traditional solid stationary phases, ensuring high recovery rates. The phytotoxic assessment against the target weeds *L. rigidum*, *P. oleracea*, and *P. lanceolata* revealed that although germination remained largely unaffected, root and shoot development was significantly inhibited in a dose-dependent manner. In particular, compounds **10** and **11** emerged as the most active analogues, exhibiting superior phytotoxicity compared to the parent compound, APO. An analysis of IC_50_ values confirms the efficacy of these modifications; for instance, compound **10** displayed an IC_50_ of 4.5 μM against *P. oleracea*, drastically lower than the 332.5 μM of natural APO. The SAR analysis indicated that electronegative substituents at the C-8 position generally enhance phytotoxicity, while an iodine atom at C-4 independently increases potency. Furthermore, the incorporation of nitrogen at positions 1 or 9 improves the selectivity toward *P. lanceolata*. Consequently, the synergistic combination of fluorine at C-8 and iodine at C-4 observed in compound **10** places these derivatives as promising lead candidates for the development of an innovative, sustainable solution for weed management.

## Supplementary Material



## Notes

Shake-Flask method data and CPC purification discussion, structural characterization of compounds **1**–**15**, and HPLC chromatograms of reaction 3, 5, 6, 7, and 9, (PDF)
