# Supplementary material for: Improving the Activity of Aminophenoxazinones: Synthesis, CPC Purification, and Phytotoxicity Potential
Source: J Agric Food Chem. 2026 Mar 4;74(10):8124–38. doi: 10.1021/acs.jafc.5c09345 (PMC13003509; doi:10.1021/acs.jafc.5c09345)
Supplement: Supplementary file 1 [file jf5c09345_si_001.pdf]

SUPPORTING INFORMATION

Improving the Activity of Aminophenoxazinones: Synthesis, CPC Purification, and Phytotoxicity Potential.

Cristina Díaz-Franco†‡, Carlos Rial†, Stefan Schwaiger‡, Rosa M. Varela†*, Francisco A. Macías† and José M.G. Molinillo†,

†Allelopathy Group, Department of Organic Chemistry, Institute of Biomolecules (INBIO), Campus de Excelencia Internacional (ceiA3), School of Science, University of Cádiz, Spain.

‡ Institute of Pharmacy/Pharmacognosy and Center for Molecular Biosciences Innsbruck (CMBI), University of Innsbruck, Innrain 80-82, 6020 Innsbruck, Austria.

*__Corresponding author__: Rosa María Varela Montoya – Full Professor of Organic Chemistry; rosa.varela@uca.es

REACTION 5 CPC INFORMATION:

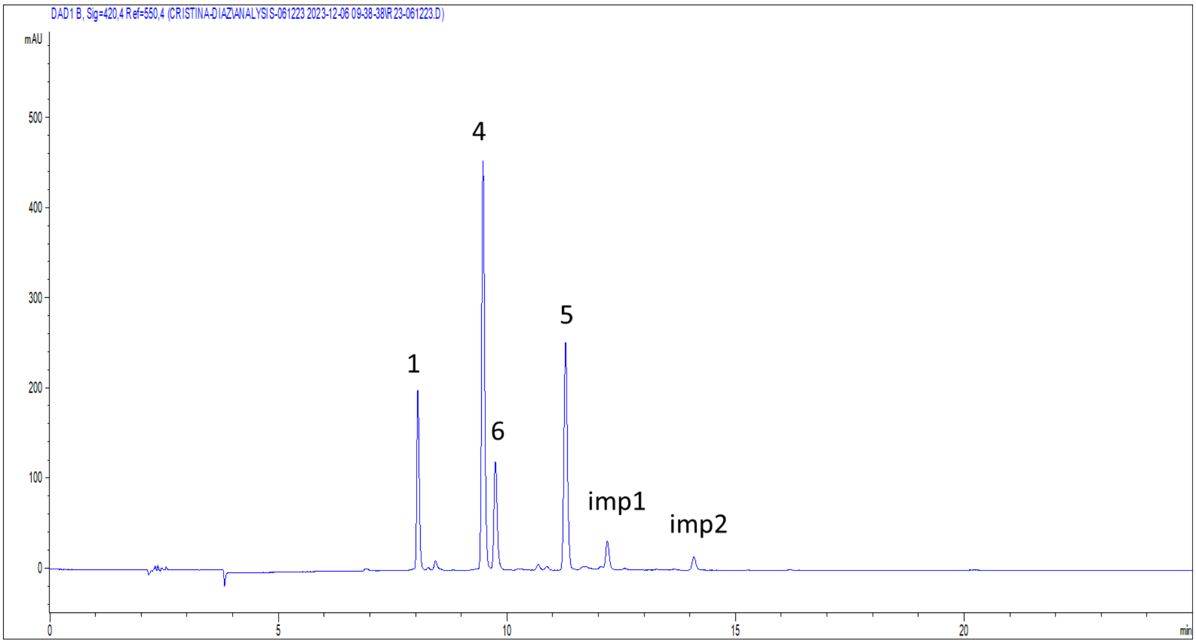

Figure S1. HPLC Chromatogram Reaction 5 (420 nm).

Reaction 5. As shown in the HPLC chromatogram (Figure S1), this sample contained 4 major compounds (**1**, **4**, **5**, and **6**) and two impurities, which were considered for the evaluation of the shake flask experiments. In total, more than 30 different solvent systems were tested and calculated for ascending mode. The selection of this mode was chosen due to the medium polarity of the target compounds, as shown in Table S1.

We decided to start an instrumental pretrial with solvent system 4 (HEMWat 0; *n*-Hex/EtOAc/MeOH/Water; 1/1/1/1, all v/v) since the partition coefficients (K_1=0.35; K_4=1.04; K_6=0.83; K_5=2.19) were over the range 0.4<K<2.5 and the separation factors ($\alpha_{I1/5}$=1.5; $\alpha_{5/4}$=2.1; $\alpha_{4/6}$=1.3; $\alpha_{6/1}$=2.4) were close to the lower recommended limit, indicating possible coelution of compounds **4**-**6**. The results achieved were consistent with the theoretical coefficients. We were able to recover 85% of the sample injected, but the sample solubility in system 4 was very poor (27%; 12.5 mg in 3 mL). This led us to modify the solvent systems by adding additives such as 1,2-dimethoxyethane (DME), dimethyl sulfoxide (DMSO), and tetrahydrofuran (THF) and performing the shake flask method again. Out of those, system 28 (HEMDmeWat +2;

Hex/EtOAc/MeOH/DME/Water; 2/1/1/1/1, all v/v) was chosen, not only because of its promising coefficients (K_1=0.62; K_4=0.84; K_6=0.95; K_5=1.46; $\alpha_{I1/5}$=1.5; $\alpha_{5/6}$=1.5; $\alpha_{6/4}$=1.1; $\alpha_{4/1}$=1.3), but also because the increased solubility of the sample in this system. Optimization of the further separation conditions was performed with system 28.

The CPC experiment was carried out in the FCPC instrument in ascending mode with system 28, with a flow rate of 0.7 mL/min at 800 rpm, which allowed us to recover 99% of the sample and enhance the sample solubility (20.9 mg in 3 mL) in comparison with system 4, reaching a total of 71% of the sample in solution. Even though the partition coefficients were promising, the separation factors were very close to the recommended limits. Additionally, the compounds were very similar, which did not ensure complete purification. Fractions were analyzed by TLC and HPLC/DAD at 420 nm, which allowed the reconstruction of a chromatogram (Figure S2A).

The system showed significant stability, with 68% stationary phase retention. The impurities were eluted at the beginning of the experiment. The brominated derivatives **5** and **4**, were eluted first with 90% and 87.2% purity, respectively. Then, we obtained APO (1) and its iodinated derivatives, **6** and **1**, with 90% and 99% purity, respectively. During the extrusion process, no compounds of interest were obtained.

Finally, upscaling was performed by transferring the optimized method to the mCPC. The reconstructed chromatogram (Figure S2B) was in line with what we obtained in the FCPC, which showed 90% recovery. The purities of the compounds and resolution were increased (**1**: 99%, **4**: 92%, **5**: 90%, and **6**: 93%), as was the retention of the stationary phase (S_f=74%). This improvement could be attributed to the increased rotation speed and larger rotor volume. On the other hand, the percentage of sample dissolution decreased from 71% to 61%, which may be due to the dilution of the sample (36.3 mg in 8 mL).

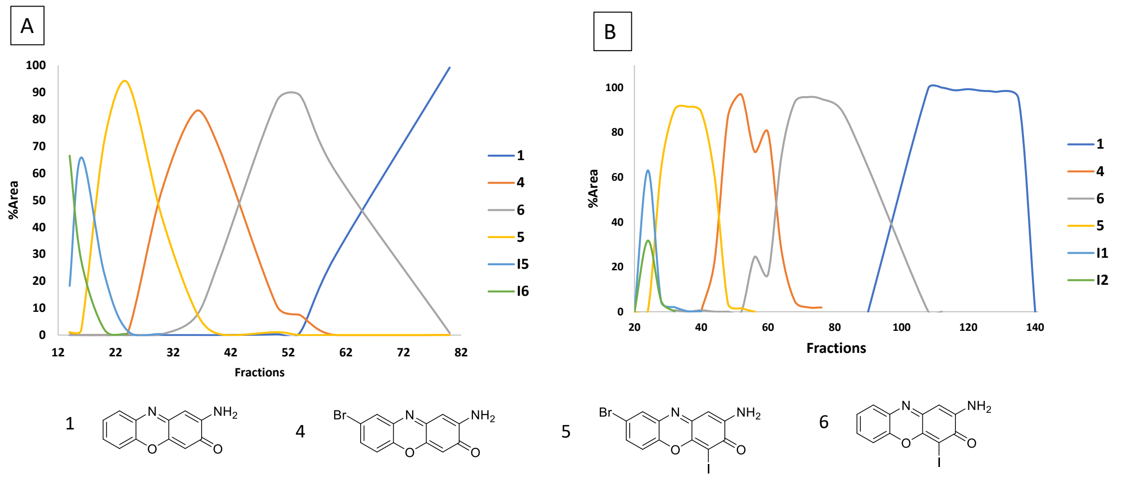

Figure S2. Reconstructed Chromatogram for separation of reaction 5 at 420 nm. A: FCPC, 0.7 mL/min at 800 rpm; B: mCPC, 4mL/min at 1200 rpm

**SHAKE FLASK METHOD REACTION 5**											
№	Solvent System	(v/v)	K_1	K_4	K_5	K_6	K_{IMP1}	K_{IMP2}	$\alpha_{1/4}$	$\alpha_{4/5}$	$\alpha_{5/6}$	$\alpha_{5/IMP1}$
n-Hexane/Ethyl Acetate/Methanol/Water											
1	HEMWat-2: 7/3/5/5		0.7	0.7	0.7	1.2	2.1	3.7	1.0	1.0	0.6	0.6
2	HEMWat -4: 7/3/6/4		0.5	1.0	0.8	1.7	2.2	3.3	0.5	1.3	0.5	0.7
3	HEMWat -7: 9/1/9/1		0.5	0.9	0.7	1.2	3.7	4.3	0.6	1.4	0.5	0.3
4	HEMWat 0: 5/5/5/5		0.4	1.0	0.8	2.2	3.3	-	0.3	1.3	0.4	0.7
5	HEMWat 2: 3/7/5/5		0.4	1.0	1.0	2.1	3.2	-	0.4	1.0	0.5	0.7
6	HEMWat 4: 3/7/4/6		1.0	0.6	2.2	1.0	2.2	4.7	1.6	0.3	2.1	0.5
7	HEMWat 7: 1/9/1/9		1.5	64.8	0.2	1.2	1.1	2.3	0.0	358.0	0.2	1.0
n-Hexane/Ethyl Acetate/1-Butanol/Methanol/Water											
8	HEMWat amp 12: 0/4/1/0/5		1.5	0.8	1.9	0.8	1.1	-	1.8	0.4	2.5	0.7
9	HEMWat amp 13: 0/3/2/0/5		1.6	0.7	2.2	1.0	1.1	1.7	2.3	0.3	2.2	0.9
10	HEMWat amp 15: 0/1/4/0/5		1.2	0.8	1.8	0.9	1.2	1.5	1.6	0.5	1.9	0.8
Tetrahydrofuran/Dimethyl sulfoxide/Water											
11	TDWat 1: 42/30/28		0.4	1.0	0.9	1.9	2.1	-	0.4	1.1	0.5	0.9
12	TDWat 2: 50/15/35		0.6	0.8	1.3	0.0	1.6	-	0.8	0.7	170.8	0.0
13	TDWat 3: 34/35/31		0.6	0.8	1.3	1.6	1.8	-	0.7	0.6	0.8	0.9
14	TDWat 4: 39/28/36		0.6	0.8	1.3	1.6	1.8	-	0.7	0.6	0.8	0.9
n-Hexane/Ethyl Acetate/Methanol/Dimethyl sulfoxide/Water											
15	HEMDWat 0: 5/5/5/5/5		0.8	1.0	1.0	1.2	1.6	-	0.8	1.0	0.8	0.8
Hexane/Ethyl Acetate/Methanol/Tetrahydrofuran/Water											
16	HEMTWat 0: 5/5/5/5/5		1.3	1.2	0.8	0.7	0.5	-	1.3	0.6	0.3	0.2
n-Hexane/Ethyl Acetate/Methanol/Dimethoxy ethane/Water											
17	HEMDmeWat 0: 5/5/5/5/5		2.3	1.6	2.4	1.8	1.9	0.8	1.4	0.7	1.3	0.9
n-Hexane/Methanol											
18	HM 0: 1/1		0.9	0.9	1.0	1.2	0.6	0.6	1.2	1.1	1.0	1.5

Table S1.Theoretical coefficients K and α for mix of compounds in R5

n-Hexane/Acetonitrile											
19	HAc 0: 1/1	1.1	0.9	1.4	1.0	0.4	-	1.3	0.6	1.3	2.9
Methyl tert-Butyl Ether/Butanol/Acetonitrile/Water											
20	tBBuAcW -6: 6/4/5/5	0.5	1.0	0.9	1.4	-	-	0.5	1.1	0.6	-
21	tBBuAcW -2: 2/2/1/5	1.0	0.9	1.2	0.9	1.1	-	1.1	0.7	1.3	0.8
22	tBBuAcW 1: 1/0/0/1	1.5	0.7	1.9	1.1	1.0	1.4	2.1	0.4	1.7	1.1
23	tBBuAcW 4: 4/0/1/5	1.5	0.7	1.9	1.1	1.1	1.7	2.1	0.4	1.8	1.0
24	tBBuAcW 6: 6/0/3/8	1.2	0.7	2.2	1.1	1.3	2.2	1.7	0.3	2.0	0.8
n-Hexane/Ethyl Acetate/Methanol/Dimethoxy ethane/Water											
25	HEMDmeWat -4: 1/1/1/2/1	0.8	0.8	1.2	1.3	1.5	2.1	1.0	0.7	0.9	0.9
26	HEMDmeWat -2: 1/1/2/1/1	0.6	0.9	0.9	1.3	2.0	2.7	0.7	0.9	0.7	0.6
27	HEMDmeWat 1: 2/1/2/1/1	0.7	0.8	1.1	1.2	2.2	3.0	0.9	0.7	0.9	0.6
28	HEMDmeWat 2: 2/1/1/1/1	0.6	0.8	1.0	1.5	2.2	3.3	0.7	0.9	0.6	0.7
29	HEMDmeWat 4: 1/2/1/1/1	0.6	1.0	0.9	1.4	1.8	2.4	0.6	1.1	0.6	0.8

Reaction 3 CPC information

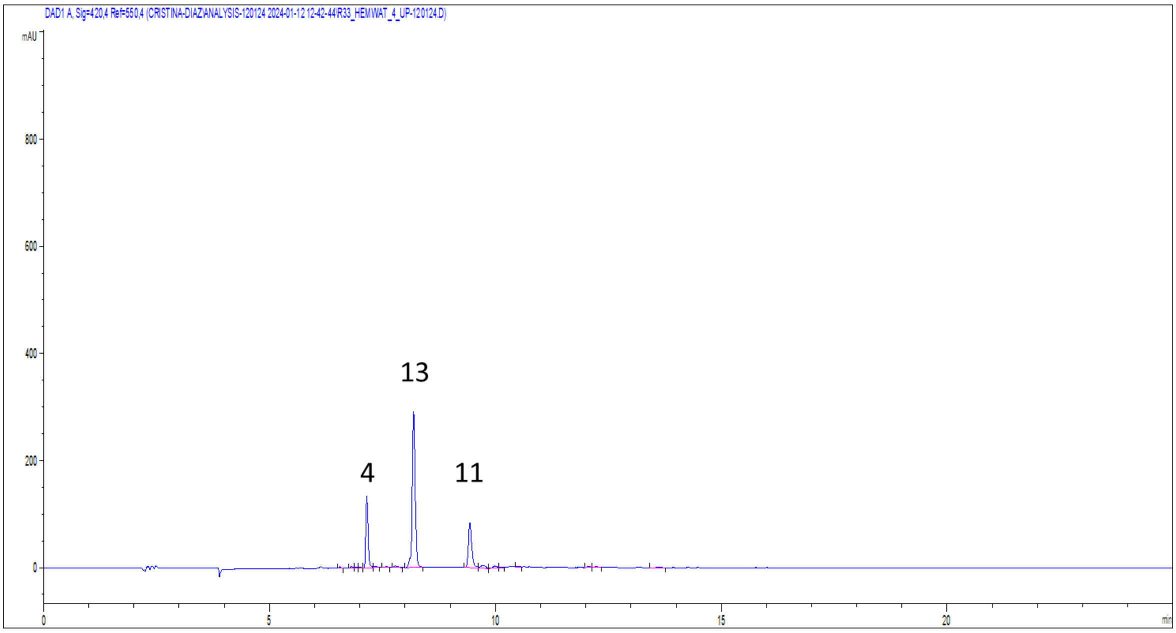

Figure S3. HPLC Chromatogram Reaction 3 (420 nm).

Reaction 3. The objective was to isolate **13** as the major component from this sample. However, the HPLC chromatogram (Figure S3) revealed the presence of **4** and **11** as well. To achieve separation, twelve different solvent systems (table S2) were evaluated using the shake flask method in ascending mode. System 4 (HEMWat 0, *n*-hexane/ethyl acetate/methanol/water; 1/1/1/1, v/v) was selected due to its partition coefficients (K_4=0.49; K_{13}=1.23; K_{11}=2.31), which were within the optimal range for effective resolution, and its separation factors ($\alpha_{13/14}$=1.9; $\alpha_{14/4}$=2.5), which ensured the isolation of compound **13**. System 4 demonstrated good stability in FCPC (S_f=68%). As illustrated in Figure S4A, the three compounds in the sample were separated with excellent purity percentages (**4:** 85%, **13**: 92%, and **11**: 91%), and the overall sample recovery was 75%. While the previous sample (Reaction 5) exhibited poor dissolution without additives, the solubility (SD) in this case reached 62% (15.5 mg in 4 mL). Consequently, this method was transferred to mCPC. The system's stability increased to 74%, and as depicted in Figure S4B, the compounds were again purified with comparable purities (**4**: 81%, **13**: 94%,

and **11**: 90%). The sample recovery improved to 86%, although the sample solubility decreased to 45% (27 mg in 8 mL).

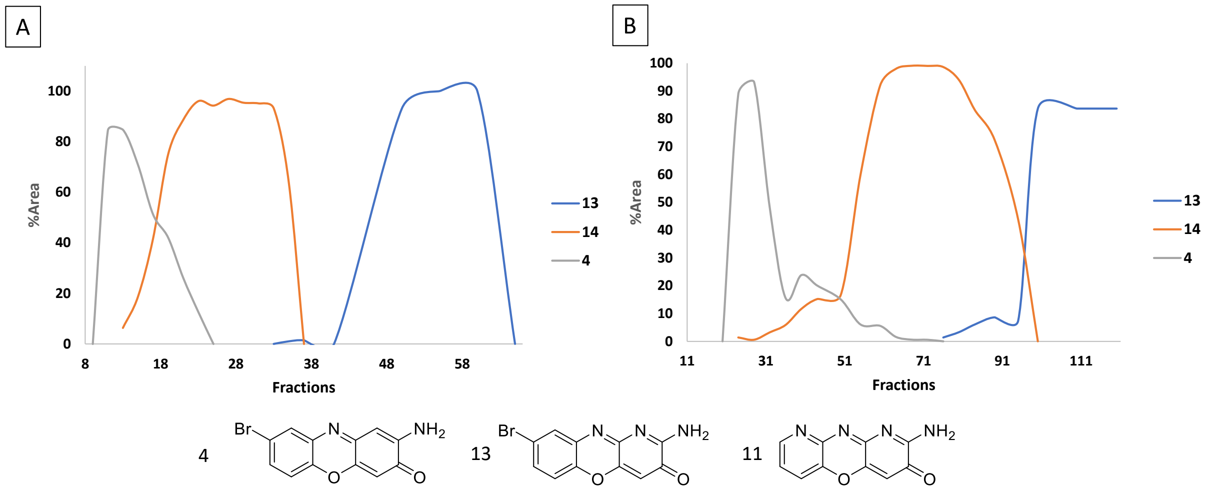

Figure S4 Reconstructed Chromatogram for separation of reaction 3 at 420 nm. A: FCPC, 1 mL/min at 800 rpm; B: mCPC, 5 mL/min at 1200 rpm.

SHAKE FLASK METHOD R3						
№	System Solvent (v/v)	K_4	K_{13}	K_{13}	$\alpha_{4/13}$	$\alpha_{13/11}$
n-Hexane/Ethyl Acetate/Methanol/Water						
1	HEMWat-2: 7/3/5/5	0.4	1.2	2.3	0.3	0.5
2	HEMWat -4: 7/3/6/4	0.4	1.1	2.4	0.3	0.5
3	HEMWat -7: 9/1/9/1	0.0	1.1	2.1	0.0	0.5
4	HEMWat 0: 5/5/5/5	0.5	1.2	2.3	0.4	0.5
5	HEMWat 2: 3/7/5/5	0.8	1.1	1.3	0.7	0.8
6	HEMWat 4: 3/7/4/6	0.6	1.1	1.7	0.6	0.6
7	HEMWat 7: 1/9/1/9	1.3	0.9	0.9	1.3	1.0
n-Hexane/Methanol						
8	HM 0: 1/1	0.3	1.3	2.1	0.2	06
n-Hexane/Ethyl Acetate/1-Butanol/Methanol/Water						
9	HEMWat amp 12: 0/4/1/0/5	1.1	0.9	0.9	1.2	1.0
10	HEMWat amp 13: 0/3/2/0/5	1.1	0.9	0.9	1.1	1.1
11	HEMWat amp 15: 0/1/4/0/5	1.0	1.0	1.0	1.0	1.0
n-Hexane/Ethyl Acetate/Methanol/Dimethoxy ethane/Water						
12	HEMDmeWat 0: 5/5/5/5/5	0.3	1.2	1.8	0.3	0.7

Table S2. Theoretical coefficients K and α for mix of compounds in R3

REACTION 6 CPC INFORMATION

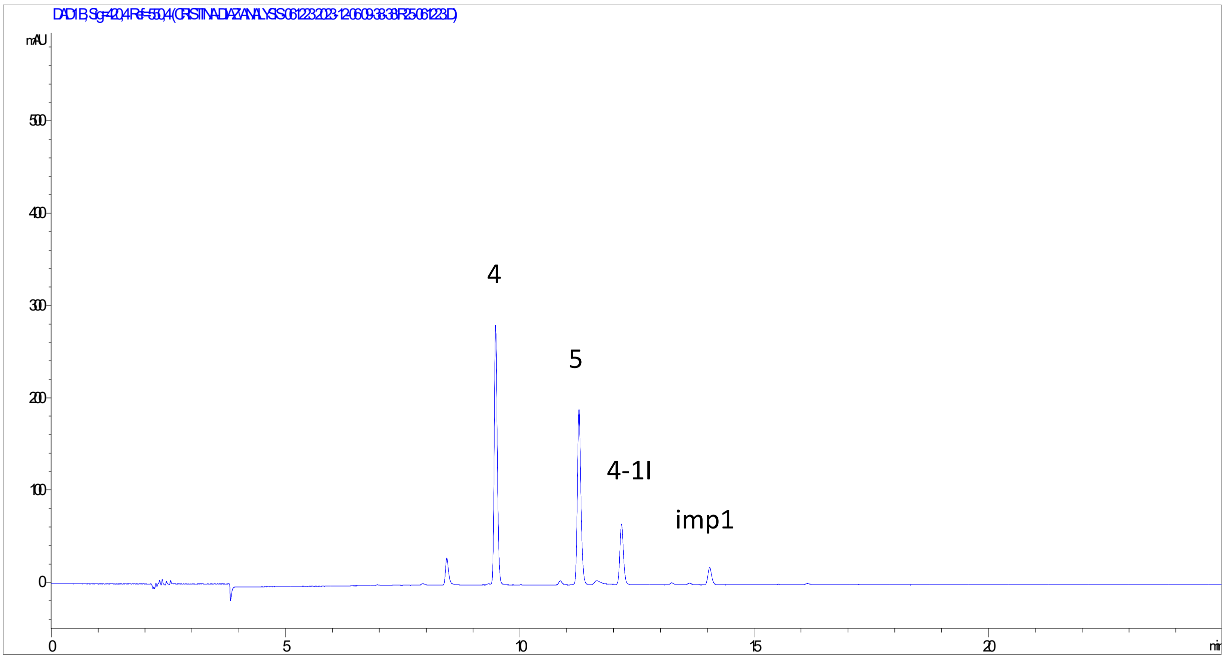

Figure S5. HPLC Chromatogram Reaction 6 (420 nm).

Reaction 6. The predominant compounds (Figure S5) in this sample were **4**, **5**, and **4-1I**, with compound **4-1I** showing the lowest yield. There was also one impurity (I1) observed. We tested 15 system solvents (Table S3) in the shake-flask method, focusing our attention on the HEMWat system with additives after checking that the HEMWat system did not result in good sample dissolution. For example, system 4 (HEMWat 0, *n*-Hex/EtOAc/MeOH/Water, 1/1/1/1, all v/v) revealed appropriate partition coefficients and separation factors (K_4=1.55; K_5=0.81; $K_{4\text{-}1I}$=0.41; K_{I1}=0.30; $\alpha_{4/5}$=1.9; $\alpha_{5/4\text{-}1I}$=2.0; $\alpha_{4\text{-}1I/I1}$=1.3), and the FCPC experiment showed separation of **4** and **5**, but the sample solubility was only 13% (4 mg in 3 mL). Therefore, we selected system 11 (HEMDmeWat 0, *n*-Hex/EtOAc/MeOH/DME/Wat; 1/1/1/1/1 v/v) to optimize the CPC conditions. System 11 showed a sample solubility of 46% (11.5 mg in 4 mL), which was a significant improvement compared to system 4, as discussed above. The coefficients for the ascending mode were in the ideal range (K_4=0.26; K_5=0.56; $K_{4\text{-}1I}$=1.46; K_{I1}=1.28), and the separation factors were promising ($\alpha_{4\text{-}1I/I1}$=1.1; $\alpha_{I1/5}$=2.5; $\alpha_{5/4}$=2.2).

The trials were carried out in the FCPC instrument. System 11 was stable (S_f=63%), allowing us to recover 99% of the sample. As shown in Figure S6A, we obtained compounds **7** and **I1** co-eluded, then, we obtained compounds **5** with 70% purity and **4** with 92% purity. We transferred this experiment to the mCPC (Figure S6B), where the system stability improved to 73%, which allowed us to increase the purities of compounds **4**, **5**, and **4-1I** to 96%, 97% and 70%, respectively.

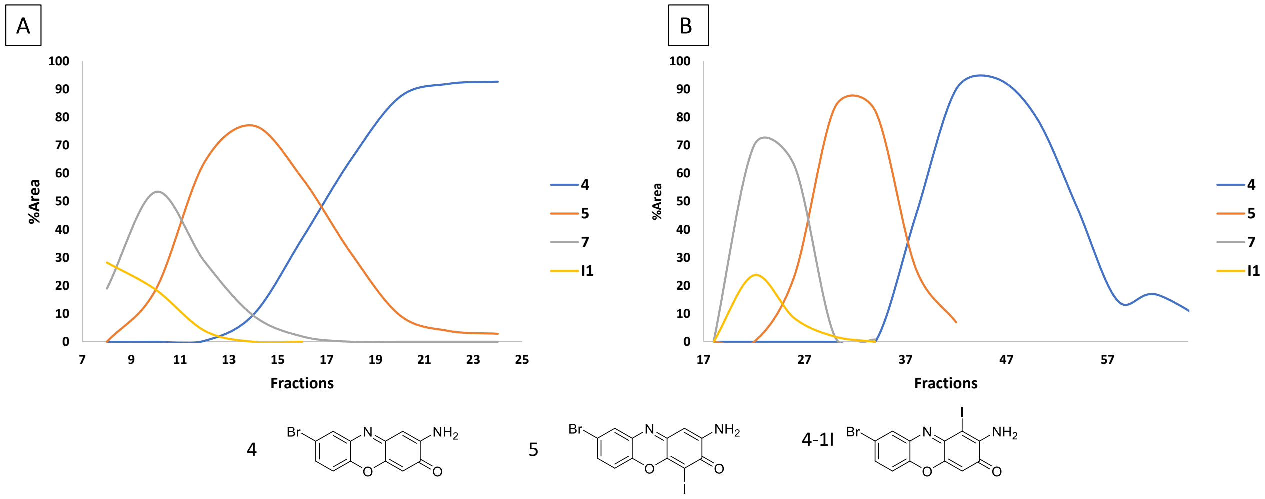

Figure S6 Reconstructed Chromatogram for separation of reaction 6 at 420 nm. A: FCPC, 1 mL/min at 800 rpm; B: mCPC, 5 mL/min at 1300 rpm.

SHAKE FLASK METHOD REACTION 6									
№	System Solvent	(v/v)	K_4	K_5	K_{4-1I}	K_{IMP1}	$\alpha_{4/5}$	$\alpha_{5/4-1}$	$\alpha_{4-1I/imp1}$
n-Hexane/Ethyl Acetate/Methanol/Water									
1	HEMWat -7: 9/1/9/1		0.7	1.1	1.8	1.7	0.6	0.6	1.1
2	HEMWat -4: 7/3/6/4		1.5	0.8	0.7	0.9	1.9	1.1	0.8
3	HEMWat-2: 7/3/5/5		0.7	1.2	1.5	1.2	0.5	0.9	1.2
4	HEMWat 0: 5/5/5/5		0.6	1.2	2.4	3.2	0.5	0.5	0.8
5	HEMWat 2: 3/7/5/5		0.6	1.4	1.9	2.8	0.5	0.7	0.7
6	HEMWat 4: 3/7/4/6		0.6	1.3	1.5	1.4	0.5	0.9	1.1
7	HEMWat 7: 1/9/1/9		0.9	1.1	0.9	0.9	0.8	1.2	1.0
n-Hexane/Ethyl Acetate/1-Butanol/Methanol/Water									
8	HEMWat amp 12: 0/4/1/0/5		0.9	1.1	0.9	0.8	0.8	1.2	1.2
9	HEMWat amp 13: 0/3/2/0/5		0.9	1.0	0.9	0.9	0.9	1.2	1.0
10	HEMWat amp 15: 0/1/4/0/5		0.9	1.1	0.9	0.8	0.8	1.2	1.1
n-Hexane/Ethyl Acetate/Methanol/Dimethyl sulfoxide/Water									
11	HEMDWat 0: 5/5/5/5/5		1.5	0.9	0.7	0.8	1.8	1.2	1.0
n-Hexane/Ethyl Acetate/Methanol/Dimethoxy ethane/Water									
12	HEMDmeWat 0: 5/5/5/5/5		3.9	1.8	0.7	0.8	2.2	2.6	0.9
n-Hexane/Ethyl Acetate/Methanol/Tetrahydrofuran/Water									
13	HEMTWat 0: 5/5/5/5/5		0.6	1.3	1.5	2.1	0.5	0.8	0.7
n-Hexane/Methanol									
14	HM 0: 1/1		1.0	1.0	1.3	1.0	1.0	0.7	1.3
n-Hexane/Acetonitrile									
15	HAcn 0: 1/1		0.9	0.9	1.6	1.2	1.0	0.5	1.3

Table S3. Theoretical coefficients K and α for mix of compounds in R6

REACTION 7 CPC INFORMATION

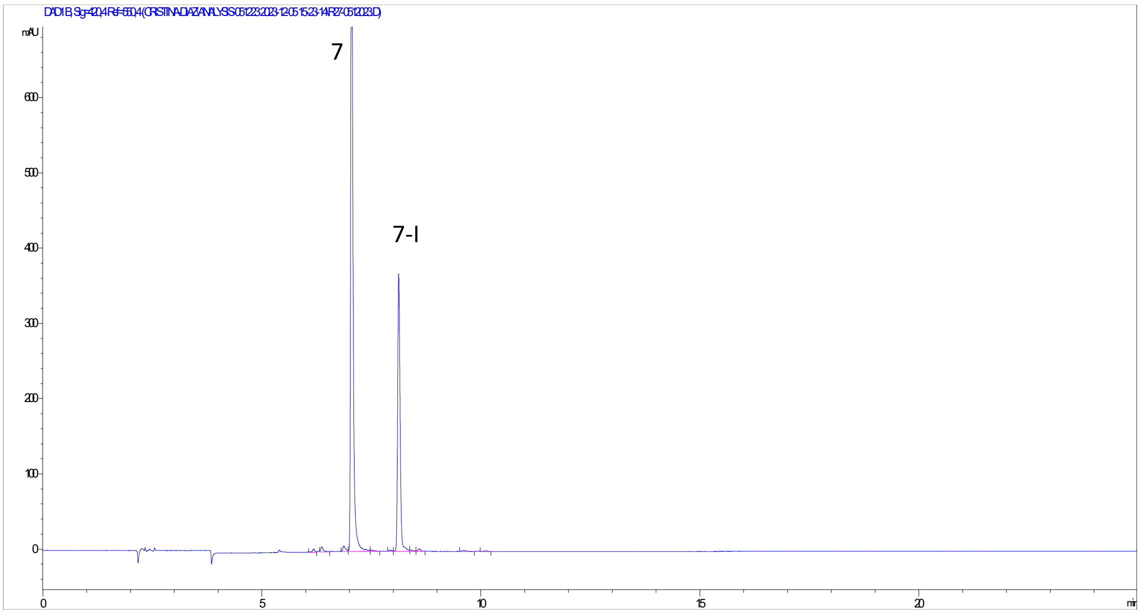

Figure S7. HPLC Chromatogram Reaction 7 (420 nm).

Reaction 7. Compounds **7** and **7-I** are the only peaks observable in the chromatogram of the HPLC analysis of reaction 7 (Figure S7), showing no evidence for further impurities. To select a fitting solvent system, we tested more than 20 systems (table S4). Even if the HEMWat systems showed good coefficients in the shake-flask trial, dissolution of the sample was particularly difficult, and none of them was able to dissolve more than 3%(<1 mg in 4 mL) of the sample. We decided to optimize the CPC conditions with system 12 (HEMDmeWat 0; *n*-Hex/EtOAc/MeOH/DME/Water; 1/1/1/1/1, all v/v), which had remarkable theoretical coefficients for descending mode (K_7= 0.83; $K_{7\text{-I}}$=1.55; $\alpha_{7\text{-I}/7}$=1.9). The results of the CPC experiment with system 12 are shown in Figure S8A. The recovery of the sample and the system stability were acceptable, with percentages of 95% and 63%, respectively. This allowed us to obtain **7-I** with 96% purity. After this, the separation trend was peculiar: **7-I** continued to elute throughout the entire method, which prevented the purification of **7**. We believe that this was due to the poor dissolution of the sample, which was 17% (6 mg in 3 mL). We decided to acidify

the system by adding 0.1% formic acid, obtaining the reconstructed chromatogram in Figure S8B. The recovery and stability of the system were adequate at 99% and 66%, respectively. Sample dissolution could not be improved, reaching a value of 10% (3.7 mg in 3 mL), and the tendency of the chromatogram reconstructed in the HPLC was the same. The significant difference was that **7** was obtained pure this time with 97% purity.

Sample dissolution improved significantly when triethylamine (TEA) was added to the solvent system. We performed several trials with system 12+0.1% TEA, but purification was not achieved. Theoretical coefficients were calculated again by adding 0.1% TEA to the HEMWat systems, which initially seemed promising, but the values indicated (Table S4) that no purification would occur and were not performed experimentally.

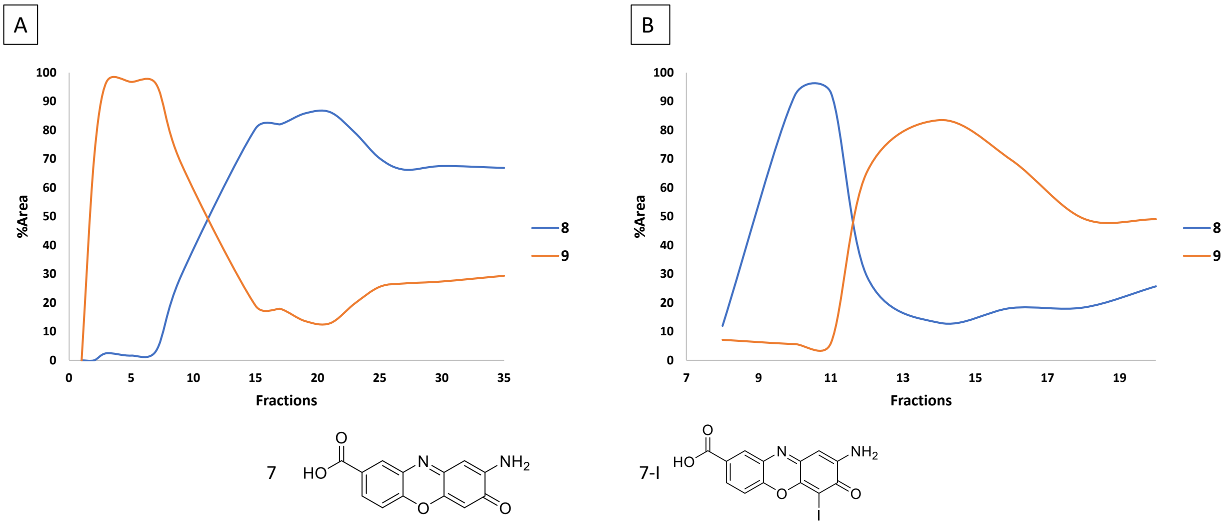

Figure S8. CPC experiment of separation of reaction 7 at 420 nm. A: FCPC system 12, 1 mL/min at 800 rpm. B: FCPC system 12 + 0.1% FA, 1 mL/min at 800 rpm

SHAKE FLASK METHOD R7					
Nº	System Solvent	(v/v)	K_7	K_{7-I}	$\alpha_{7/7-1}$
n-Hexane/Ethyl Acetate/Methanol/Water					
1	HEMWat -7: 9/1/9/1		1.5	0.0	-
2	HEMWat -4: 7/3/6/4		1.0	0.7	1.4
3	HEMWat-2: 7/3/5/5		1.4	0.5	2.6
4	HEMWat 0: 5/5/5/5		1.4	0.5	2.7
5	HEMWat 2: 3/7/5/5		1.3	0.6	2.3
6	HEMWat 4: 3/7/4/6		1.6	0.4	3.6
7	HEMWat 7: 1/9/1/9		1.6	0.4	3.7
n-Hexane/Ethyl Acetate/1-Butanol/Methanol/Water					
8	HEMWat amp 12: 0/4/1/0/5		1.5	0.4	3.7
9	HEMWat amp 13: 0/3/2/0/5		1.6	0.4	4.2
10	HEMWat amp 15: 0/1/4/0/5		1.6	0.4	4.3
n-Hexane/Ethyl Acetate/Methanol/Dimethyl sulfoxide/Water					
11	HEMDWat 0: 5/5/5/5/5		0.9	2.1	0.4
n-Hexane/Ethyl Acetate/Methanol/dimethoxy ethane/Water					
12	HEMDmeWat 0: 5/5/5/5/5		1.2	0.6	1.9
n-Hexane/Ethyl Acetate/Methanol/Tetrahydrofuran/Water					
13	HEMTWat 0: 5/5/5/5/5		0.8	1.5	0.6
n-Hexane/Acetonitrile					
14	HAc 0: 1/1		0.8	-	-
n-Hexane/Methanol					
15	HM 0: 1/1		1.1	0.8	-
n-Hexane/Ethyl Acetate/Methanol/Water + 0.1% Triethylamine					
16	HEMWat -7: 9/1/9/1 + 0.1% TEA		1.0	0.8	1.3
17	HEMWat -4: 7/3/6/4 + 0.1% TEA		1.0	1.0	1.0
18	HEMWat-2: 7/3/5/5 + 0.1% TEA		1.0	1.0	0.9
19	HEMWat 0: 5/5/5/5 + 0.1% TEA		1.1	0.7	1.5
20	HEMWat 2: 3/7/5/5 + 0.1% TEA		1.0	0.9	1.2

21	HEMWat 4: 3/7/4/6 + 0.1% TEA	1.0	0.9	1.2
22	HEMWat 7: 1/9/1/9 + 0.1% TEA	1.0	0.9	1.1

Table S4. Theoretical coefficients K and α for mix of compounds in R7

REACTION 9 CPC INFORMATION

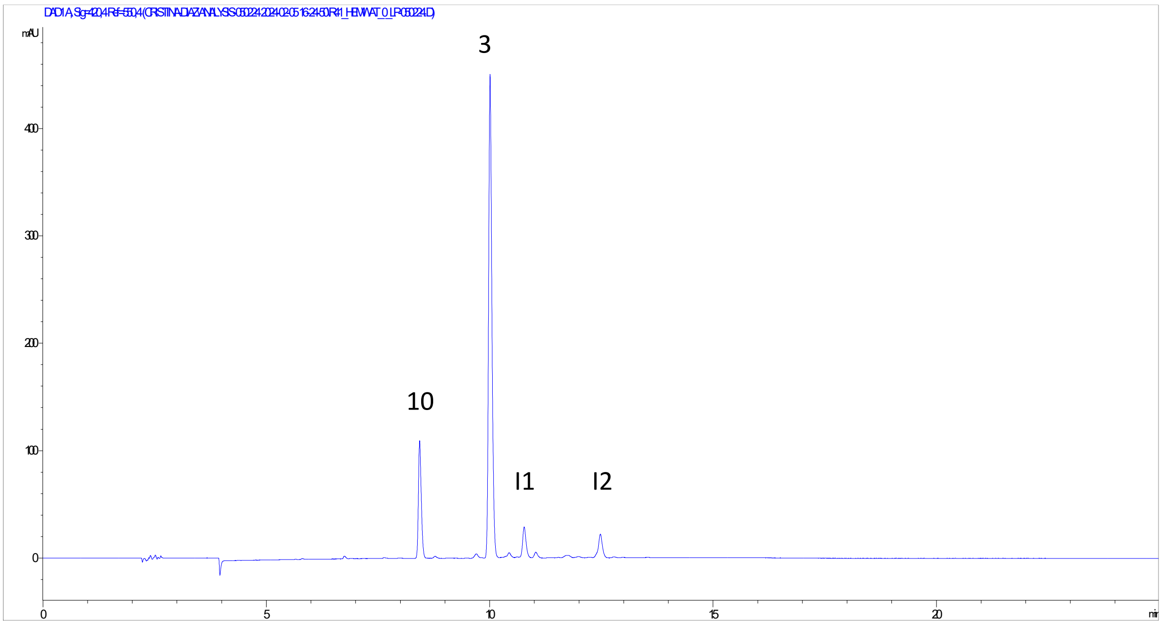

Figure S9. HPLC Chromatogram Reaction 9 (420 nm).

Reaction 9. In this sample (Figure S9), we had two impurities (I1 and I2), **3** and **10**. The impurities had theoretical coefficients lower than 0.2, which means that they will not be retained in stationary phase and leave the equipment once the experiment starts. We calculated the K and α (table S5) coefficients for 14 different system solvents and selected system 11 (HEMDmeWat 0; *n*-Hex/EtOAc/MeOH/DME/Water; 1/1/1/1/1, all v/v). The coefficients in ascending mode (K_3=0.78; K_{12}=2.37; $\alpha_{12/3}$=3.0) were promising, and we started pretrials in the FCPC. Figure S10A shows the results of the pretrials: the impurities were eluted from the FCPC, almost when the experiment started, as predicted by the determined coefficients. We obtained **3** and **10** with 94% and 90% purity, respectively. The recovery of the sample and stability of system were high (97% and 68%, respectively), and the sample dissolution reached 60% (12.2 mg in 3 mL).

For an up-scale experiment, the conditions were transferred to the cCPC instrument utilizing the 1000 mL rotor. The chromatogram provided (Figure S10B) by the equipment showed better resolution because of the increased rotation speed. The recovery rate and stability of the system were similar to those of the FCPC system, with percentages of 99% and 67%, respectively. This

allowed us to obtain **3** with 99% purity and **10** with 97% purity. Sample dissolution increased to 65% (19.5 mg in 8 mL) due to the increase in volume used for injection.

CPC enabled us to solve the problems caused by the strong interaction of APO derivatives with solid stationary phases; we recovered almost 100% of the injected sample in all trials, and the purities of the compounds reached 90–99%. The pretrials conducted to obtain theoretical coefficients and separation factors were crucial for achieving satisfactory purification results. However, it is important to emphasize that even with adequate coefficients, the properties of the sample matrix, such as the nature of the compounds and the solvent system, as well as additional experimental settings, significantly influence the final purification outcome.

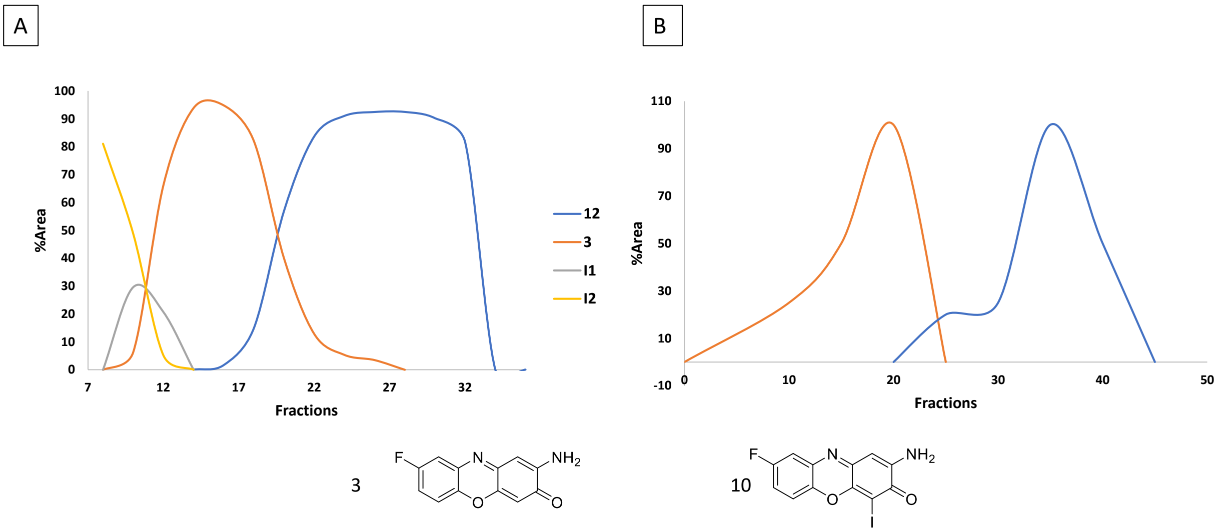

Figure S10 A: Reconstructed chromatogram of FCPC separation of reaction 9 at 420 nm; 1 mL/min at 800 rpm. B: Chromatogram of cCPC; 5 mL/min at 2500 rpm.

SHAKE FLASK METHOD R9					
№	System Solvent	(v/v)	K_{10}	K_3	$\alpha_{10/3}$
n-Hexane/Ethyl Acetate/Methanol/Water					
1	HEMWat -7: 9/1/9/1		1.1	1.0	1.1
2	HEMWat -4: 7/3/6/4		1.3	1.0	1.3
3	HEMWat-2: 7/3/5/5		2.0	0.9	2.2
4	HEMWat 0: 5/5/5/5		0.6	1.1	0.6
5	HEMWat 2: 3/7/5/5		1.6	0.9	1.7
6	HEMWat 4: 3/7/4/6		1.7	1.0	1.7
7	HEMWat 7: 1/9/1/9		1.3	1.1	1.2
n-Hexane/Methanol					
8	HM 0: 1/1		0.8	1.1	0.7
9	HAcn 0: 1/1		0.5	0.9	0.6
n-Hexane/Ethyl Acetate/Methanol/Tetrahydrofuran/Water					
10	HEMTWat 0: 5/5/5/5/5		1.7	0.8	2.1
n-Hexane/Ethyl Acetate/Methanol/Dimethoxy ethane/Water					
11	HEMDmeWat 0: 5/5/5/5/5		2.4	0.8	3.0
12	HEMDmeWat 1: 2/1/2/1/1		1.3	0.9	1.4
13	HEMDmeWat 2: 2/1/1/1/1		1.6	0.9	1.7
14	HEMDmeWat -2: 1/1/2/1/1		1.8	0.9	1.9

Table S5. Theoretical coefficients K and α for mix of compounds in R9

Structural characterization of 2-amino-3*H*-phenoxazin-3-one (**1**)

2-Amino-3*H*-phenoxazin-3-one, dark red powder (**1**, 14% yield), ^{1}H-NMR (DMSO-d_6, 600 MHz) δ (ppm) 7.71 (1H, dd, *J*= 8.0; 1.5 Hz, H-9), 7.51 (1H, dd, *J*=8.2; 1.3 Hz, H-6), 7.47 (1H, ddd, *J*=8.0, 1.5, H-7), 7.40 (1H, ddd, *J*= 7.9; 1.5 Hz, H-8), 6.80 (2H,bs, NH_2), 6.37 (1H, s, H-4), 6.37 (1H, s, H-1); ^{13}C-NMR (DMSO-d_6, 150 MHz) δ (ppm) 180.2 (C, C-3), 148.9 (C, C-2), 148.2 (C, C-4a), 147.3 (C, C-10a), 141.9 (C, C-5a), 133.7 (C, C-9a), 128.8 (CH, C-7), 127.9 (CH, C-9), 125.3 (CH, C-8), 115.9 (CH, C-6), 103.4 (CH, C-4), 98.3 (CH, C-1). HRESIMS *m/z* 213.0680 $[M+H]^+$ (calcd for $C_{12}H_9N_2O_2$, 213.0664).

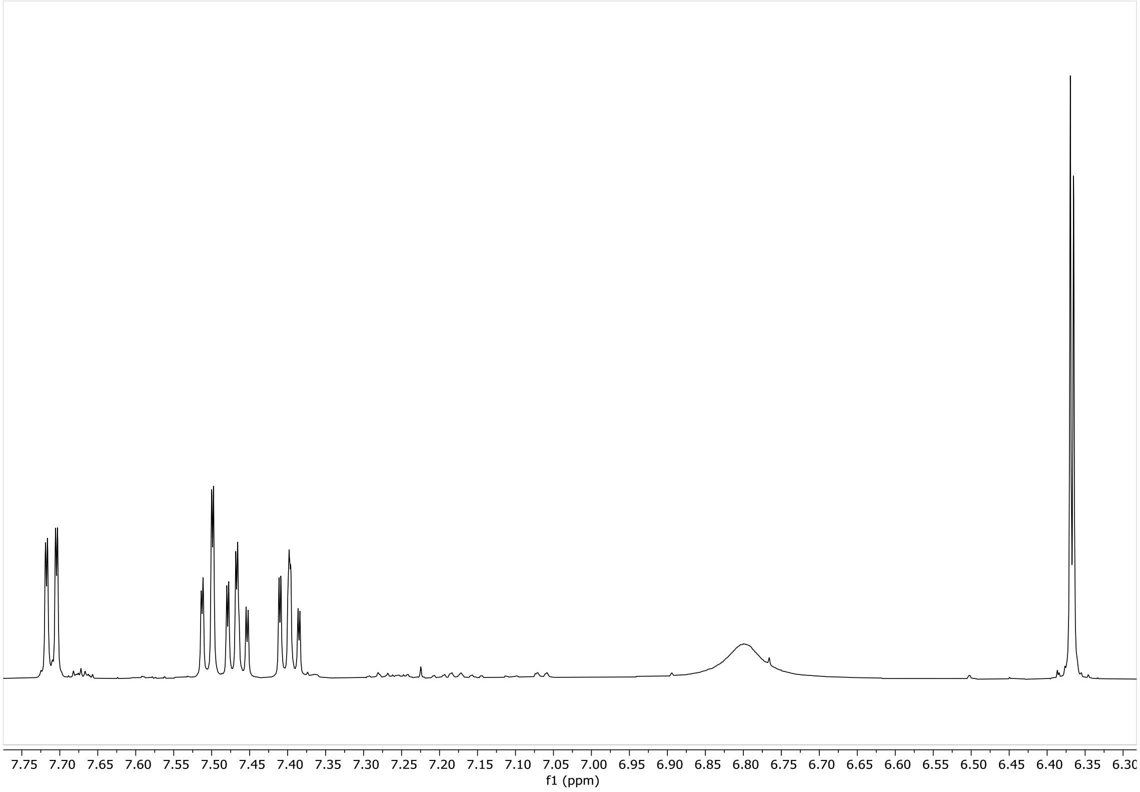

Figure S11 ^{1}H-NMR Spectrum of 2-amino-3H-phenoxazin-3-one.

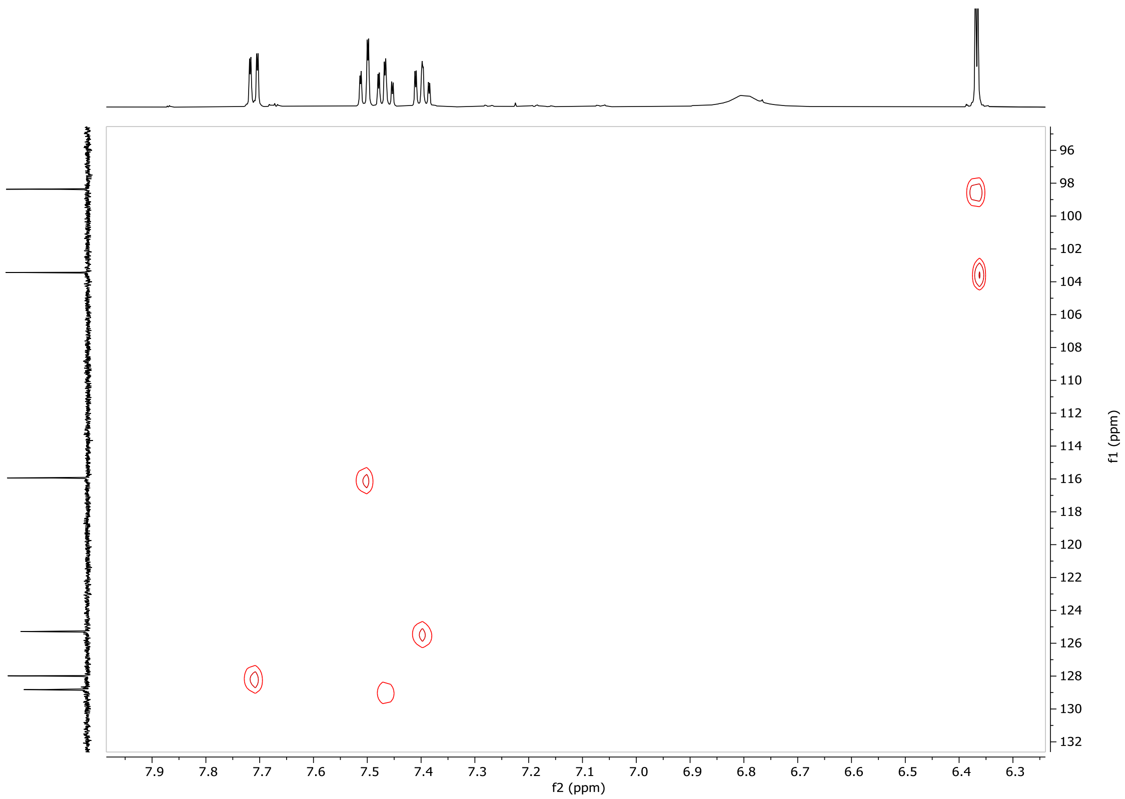

Figure S12 HSQC Spectrum of 2-amino-3H-phenoxazin-3-one

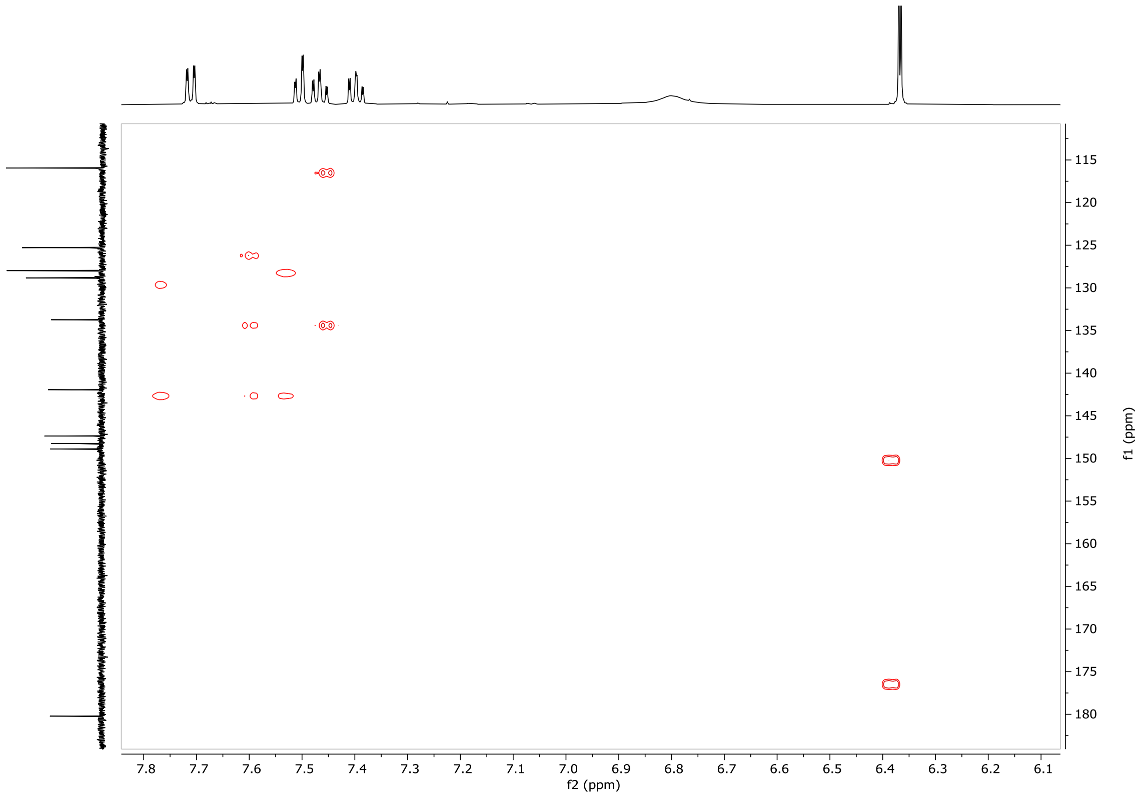

Figure S13 HMBC spectrum of 2-amino-3H-phenozaxin-3-one

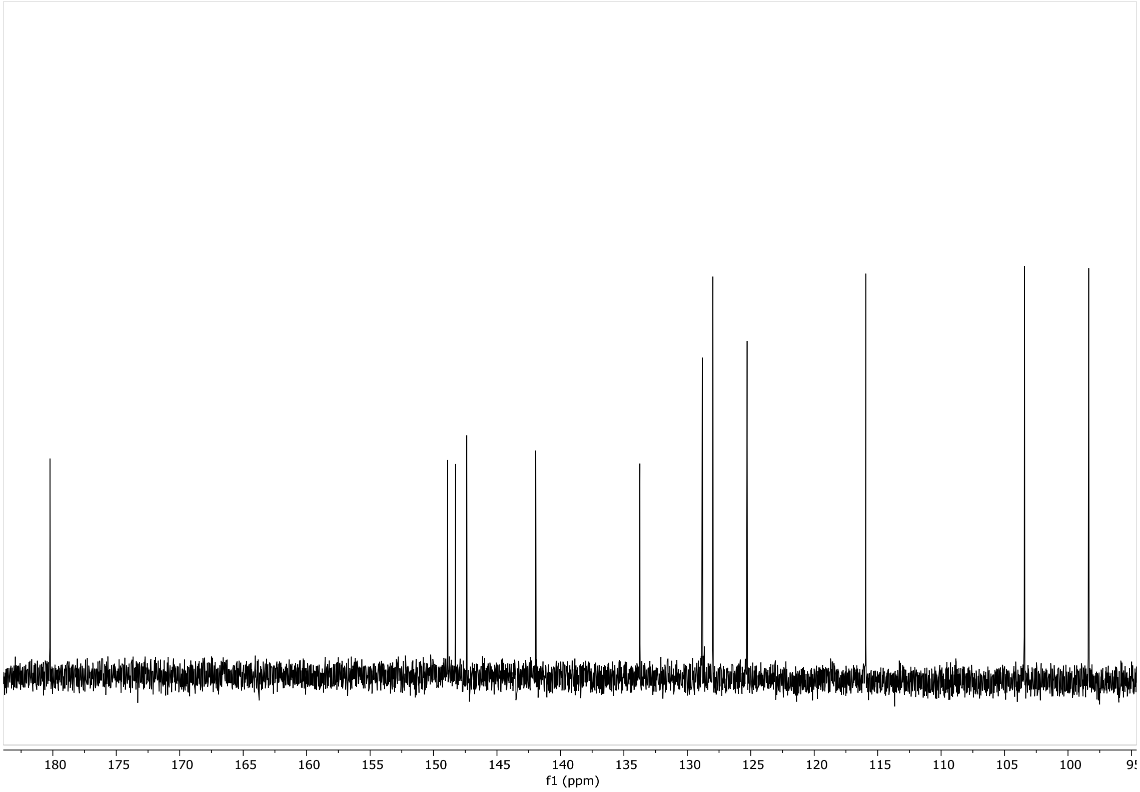

Figure S14 ^{13}C-NMR Spectrum of 2-amino-3H-phenoxazin-3-one

Structural characterization of 2-amino-4,8-dibromo-1*H*-phenoxazin-1-one (**2**)

2-Amino-4,8-dibromo-1*H*-phenoxazin-1-one (**2**), dark orange powder, ^{1}H-NMR (DMSO-d_6, 500 MHz) δ (ppm) 7.95 (1H, d, *J*= 2.2 Hz, H-9), 7.65 (1H, dd, *J*= 8.8; 2.2 Hz, H-7), 7.51 (1H, d, *J*= 8.8 Hz, H-6), 7.17 (2H, bs, NH_2), 6.41 (1H, s, H-3); ^{13}C-NMR (DMSO-d_6, 125 MHz) δ (ppm) 177.9 (C, C-1), 149.9 (C, C-2), 146.5 (C, C-4a), 145.3 (C, C-10a), 141.9 (C, C-5a), 134.9 (C, C-9a), 132.3 (CH, C-7), 130.5 (CH, C-9), 118.3 (CH, C-6), 117.7 (C, C-8), 103.2 (C, C-3), 94.8 (C, C-4). HRESIMS *m/z* 368.8877 $[M+H]^+$ (calcd for $C_{12}H_7Br_2N_2O_2$, 368.8874).

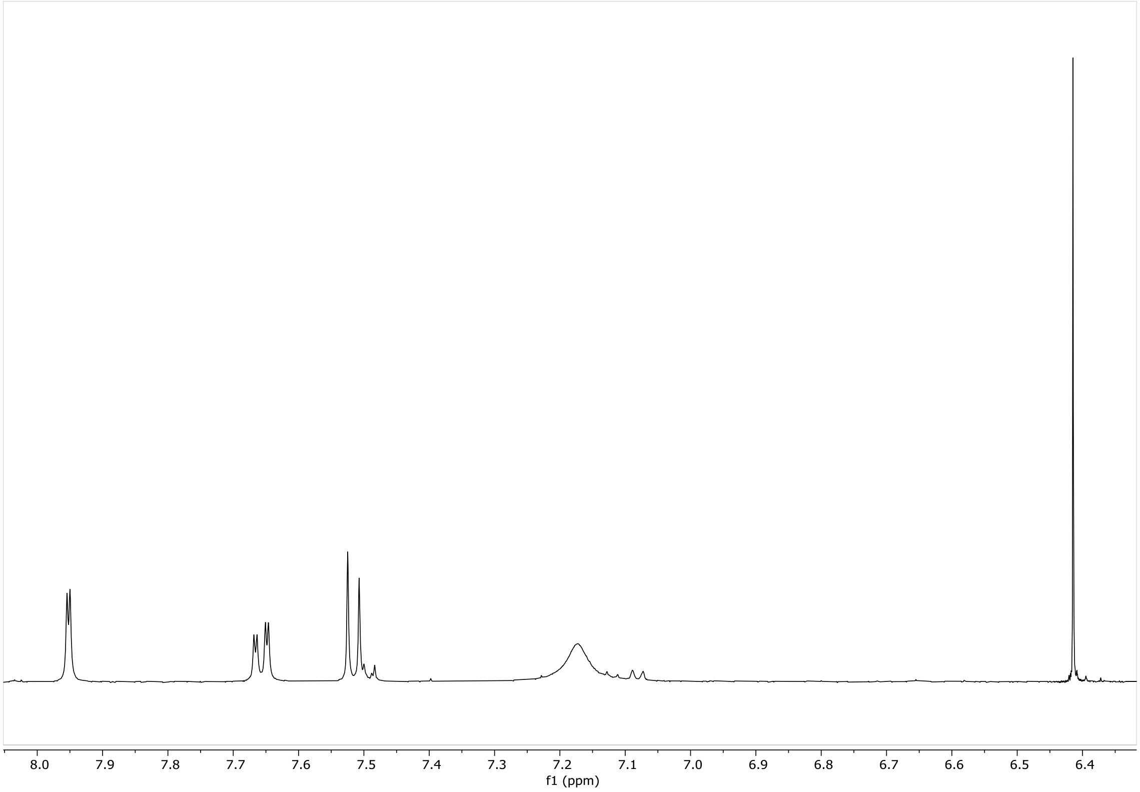

Figure S15 ^{1}H-NMR Spectrum of 2-amino-4,8-dibromo-1H-phenoxazin-1-one

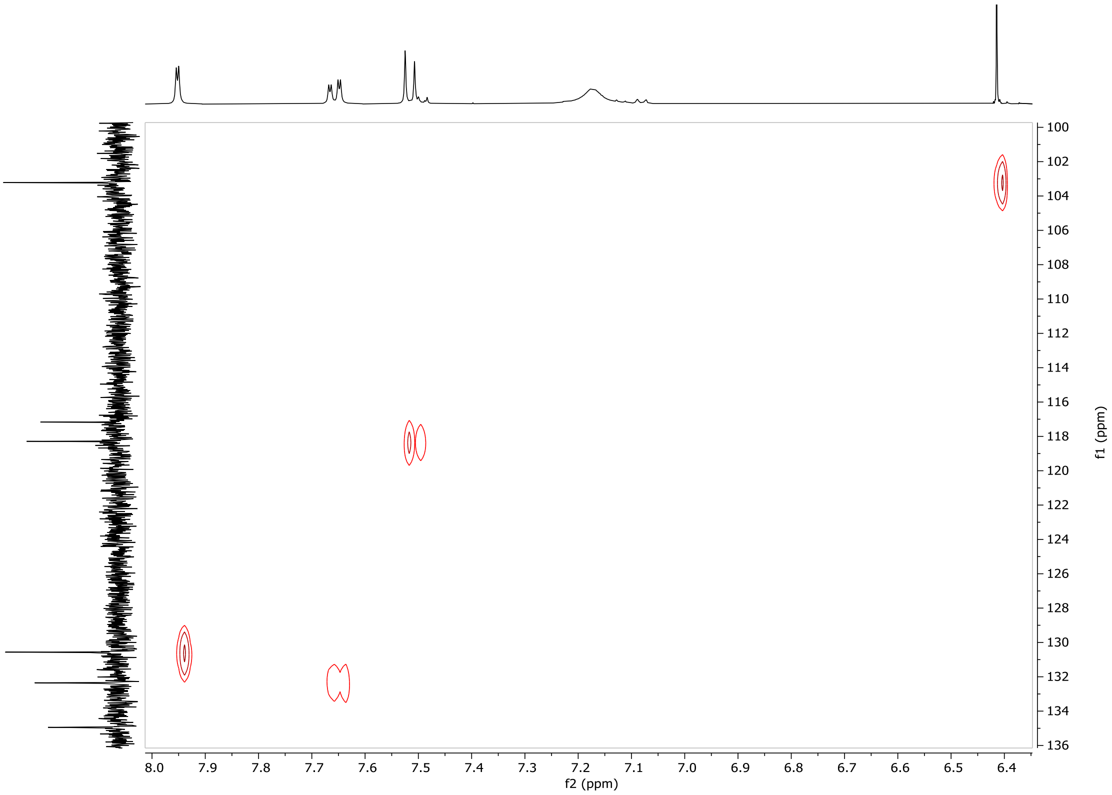

Figure S16 HSQC Spectrum of 2-amino-4,8-dibromo-1H-phenoxazin-1-one

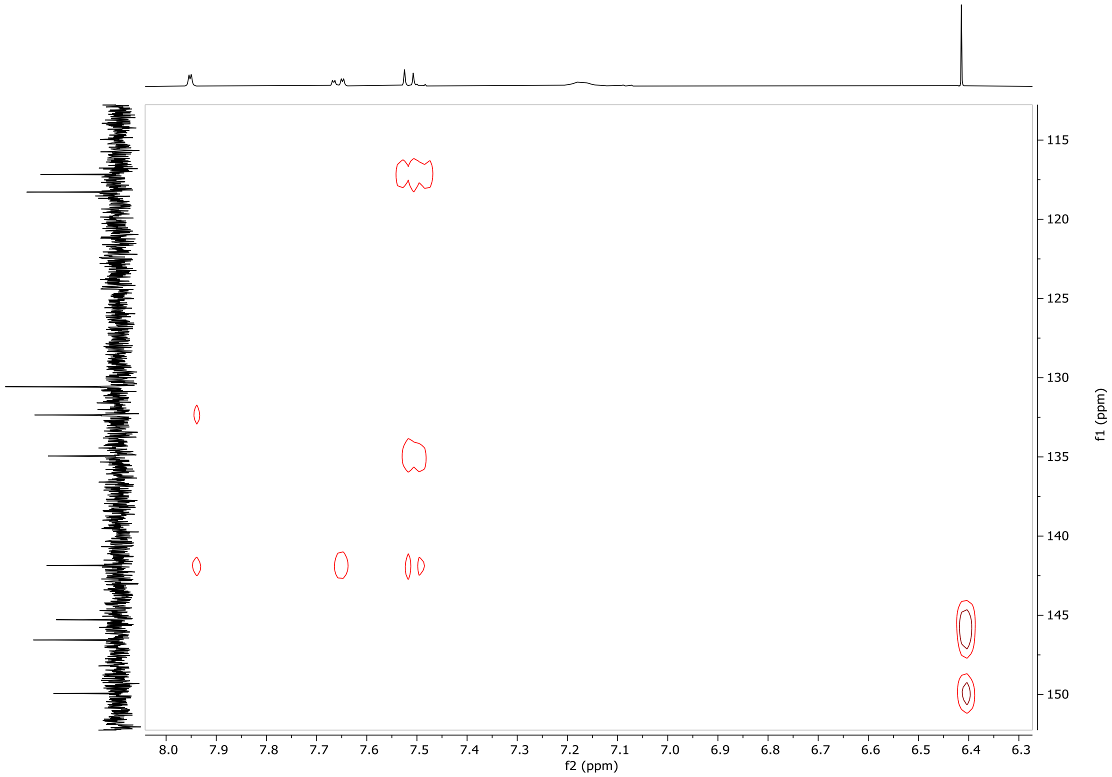

Figure S17. HMBC Spectrum of 2 amino-4,8-dibromo-1H-phenoxazin-1-one

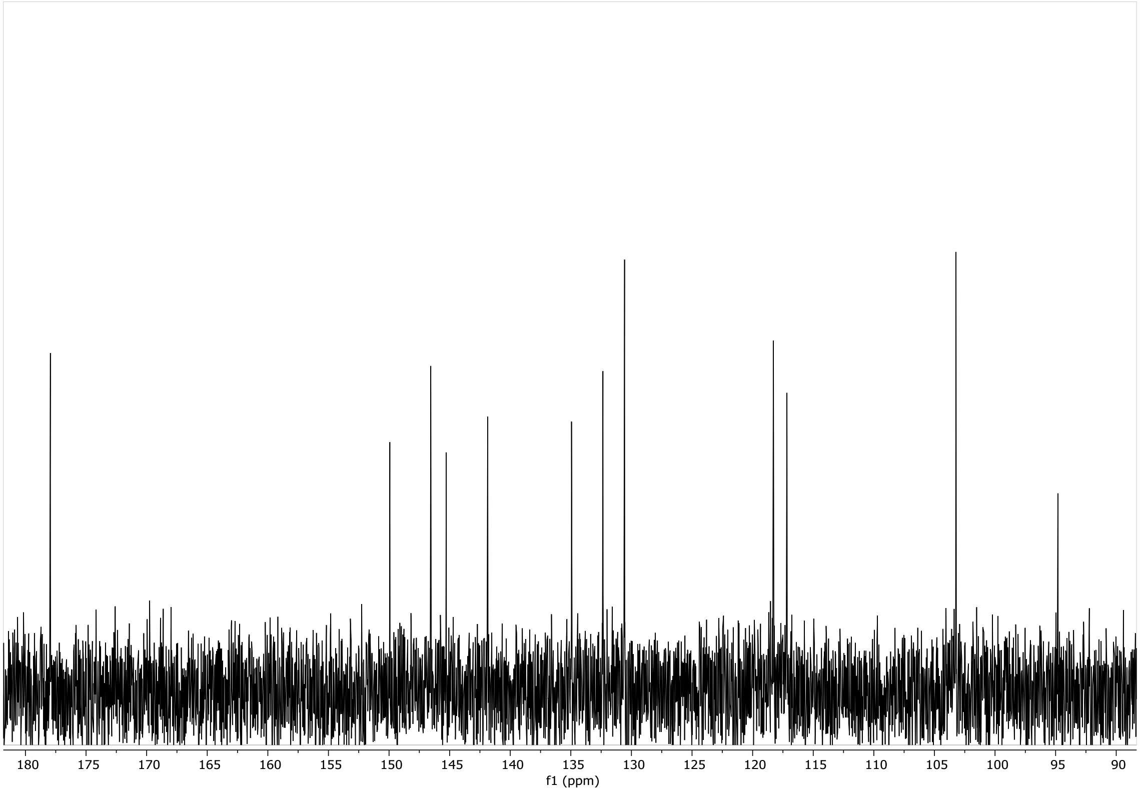

Figure S18 ^{13}C-NMR Spectrum of 2 amino-4,8-dibromo-1H-phenoxazin-1-one

Structural characterization of 2-amino-8-fluoro-3*H*-phenoxazin-3-one (**3**)

2-Amino-8-fluoro-3*H*-phenoxazin-3-one (**3**), dark yellow powder, ^{1}H-NMR (DMSO-d_6, 500 MHz) δ (ppm) 7.56 (1H, dd, *J*= 8.9; 5.0 Hz, H-6), 7.53 (1H, dd, *J*= 9.3; 3.0 Hz, H-9), 7.33 (1H, ddd, *J*= 9.0; 8.4; 3.1 Hz, H-7), 7.0 (2H, bs, NH_2), 6.36 (1H, s, H-4), 6.35 (1H, s, H-1); ^{13}C-NMR (DMSO-d_6, 125 MHz) δ (ppm) 180.1 (C, C-3), 158.7 (C, $J_{C\text{-}F}$= 240 Hz, C-8),149.0 (C, C-2) 148.9 (C, C-4a), 147.9 (C, C-10a), 138.5 (C, $J_{C\text{-}F}$= 2.2 Hz, C-5a), 134.5 (C, $J_{C\text{-}F}$= 13.6 Hz, C-9a), 117.3 (CH, $J_{C\text{-}F}$= 9.7 Hz, C-6), 115.7 (CH, $J_{C\text{-}F}$= 24.6 Hz, C-7), 112.0 (CH, $J_{C\text{-}F}$= 24.6 Hz, C-9), 103.4 (CH, C-4), 97.9 (CH, C-1). HRESIMS *m/z* 229.0416 $[M\text{-}H]^-$ (calcd for $C_{12}H_6FN_2O_2$, 229.0413).

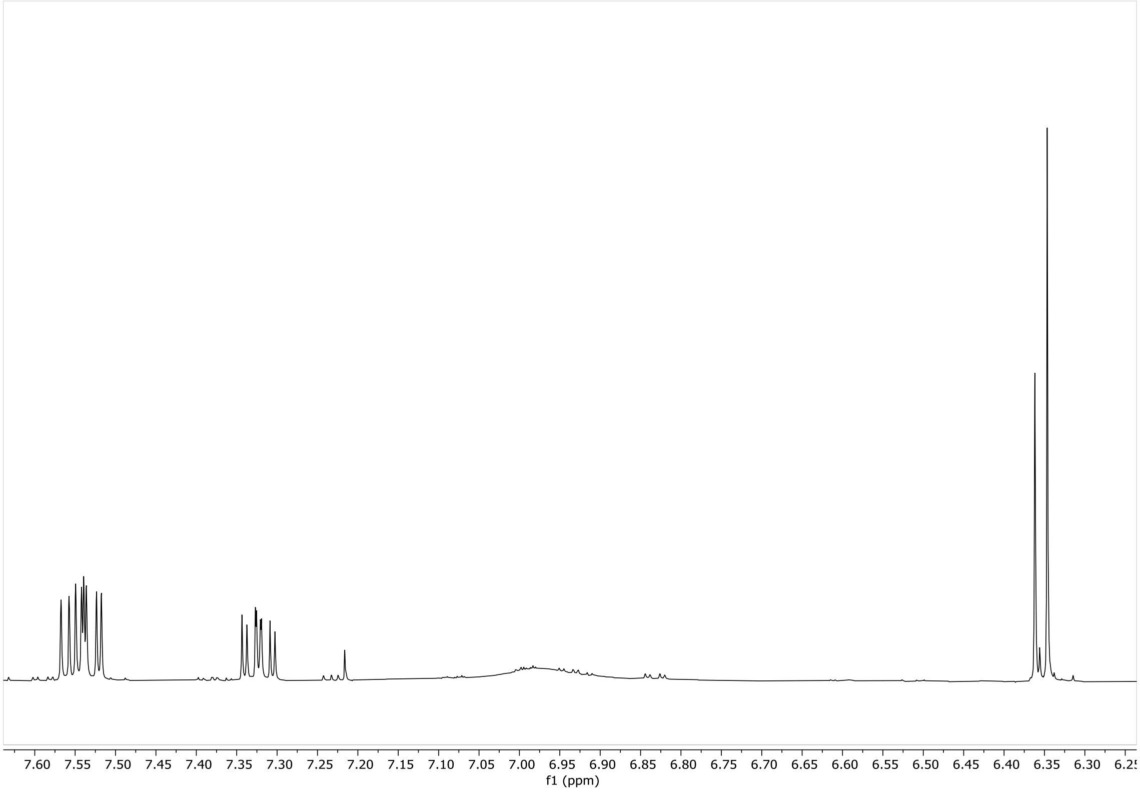

Figure S19 ^{1}H-NMR spectrum of 2-amino-8-fluoro-3H-phenoxazin-3-one

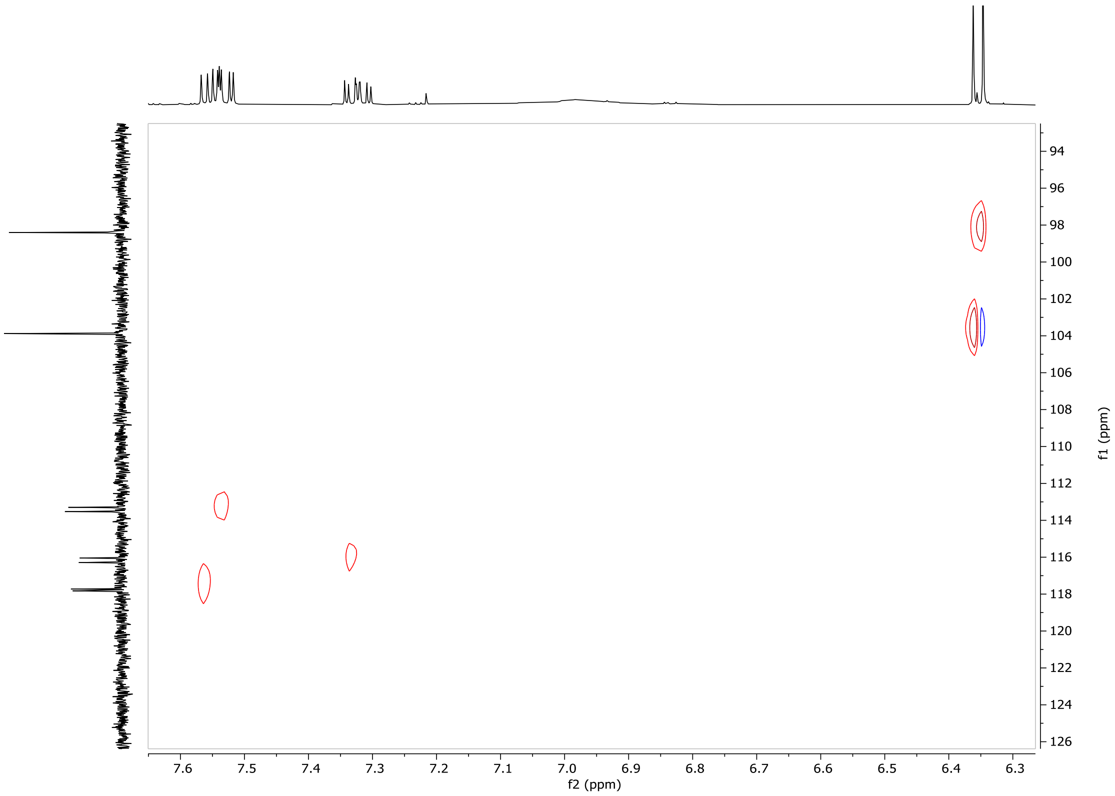

Figure S20. HSQC spectrum of 2-amino-8-fluoro-3H-phenoxazin-3-one

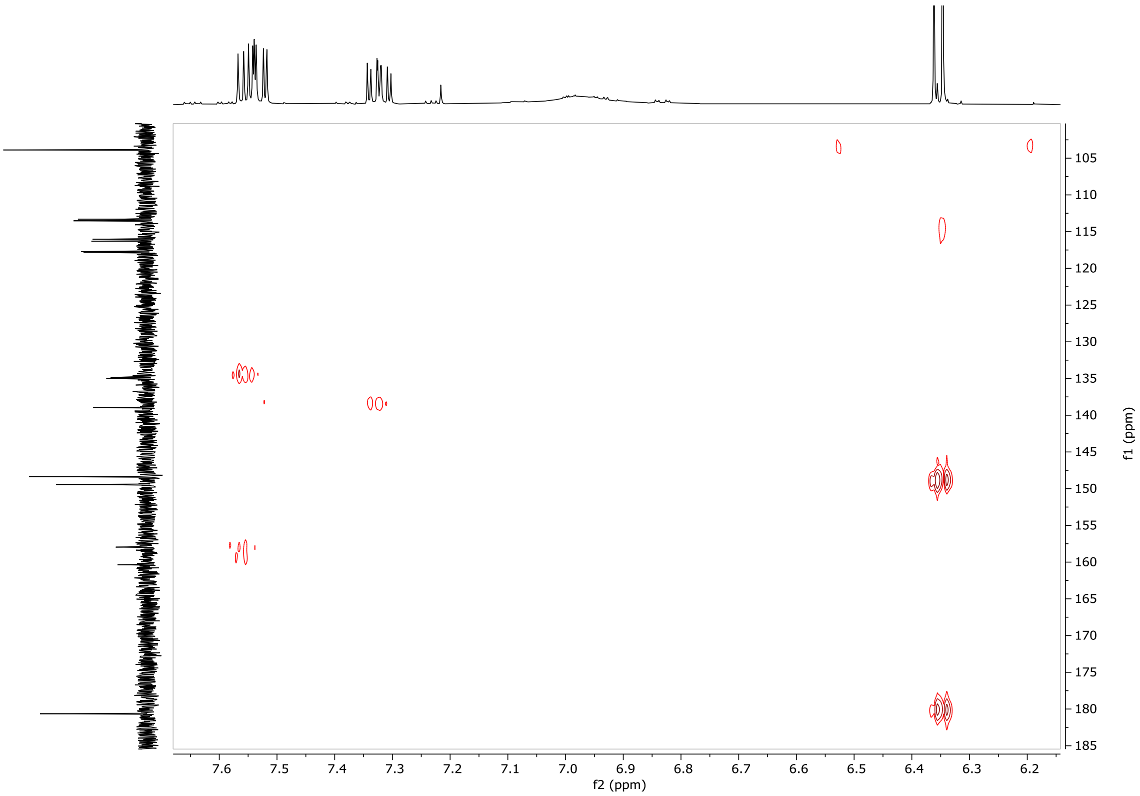

Figure S21. HMBC Spectrum of 2-amino-8-fluoro-3H-phenoxazin-3-one

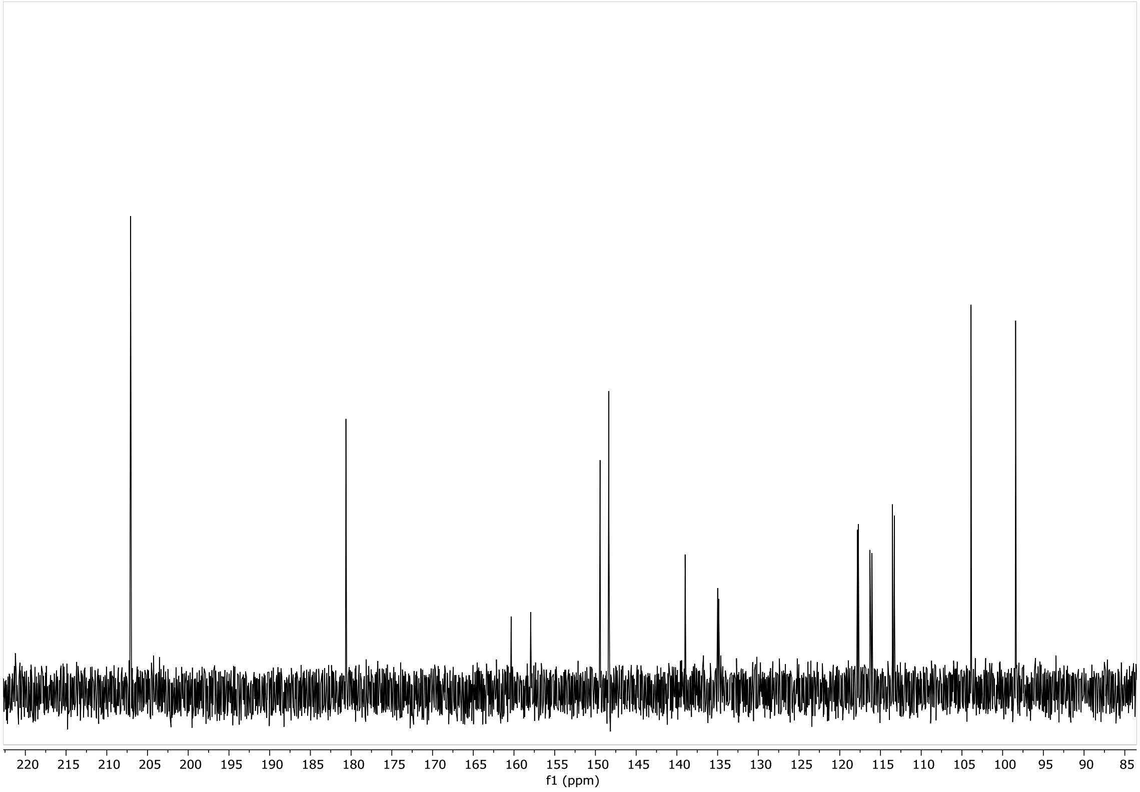

Figure S22. ^{13}C-NMR Spectrum of 2-amino-8-fluoro-3H-phenoxazin-3-one

Structural characterization of 2-amino-8-bromo-3*H*-phenoxazin-3-one (**4**)

2-Amino-8-bromo-3*H*-phenoxazin-3-one (**4**), dark orange powder, ^{1}H-NMR (DMSO-d_6, 600 MHz) δ (ppm) 7.87 (1H, d, *J*= 2.4 Hz, H-9), 7.58 (1H, dd, *J*= 8.5; 2.4 Hz, H-7), 7.47 (1H, d, *J*= 8.9 Hz, H-6),6.97 (2H, bs, NH_2); 6.37 (1H, s, H-4), 6.35 (1H, s, H-1); ^{13}C-NMR (DMSO-d_6, 150 MHz) δ (ppm) 180.2 (C, C-3), 149.0 (C, C-2), 148.7 (C, C-4a), 147.8 (C, C-10a), 141.2 (C, C-5a), 135.1 (C, C-9a), 130.8 (CH, C-7), 129.6 (CH, C-9), 117.9 (CH, C-6), 116.5 (C, C-8), 103.7 (CH, C-4), 98.1 (CH, C-1). HRESIMS *m/z* 290.9773 $[M+H]^+$ (calcd for $C_{12}H_8BrN_2O_2$, 290.9769).

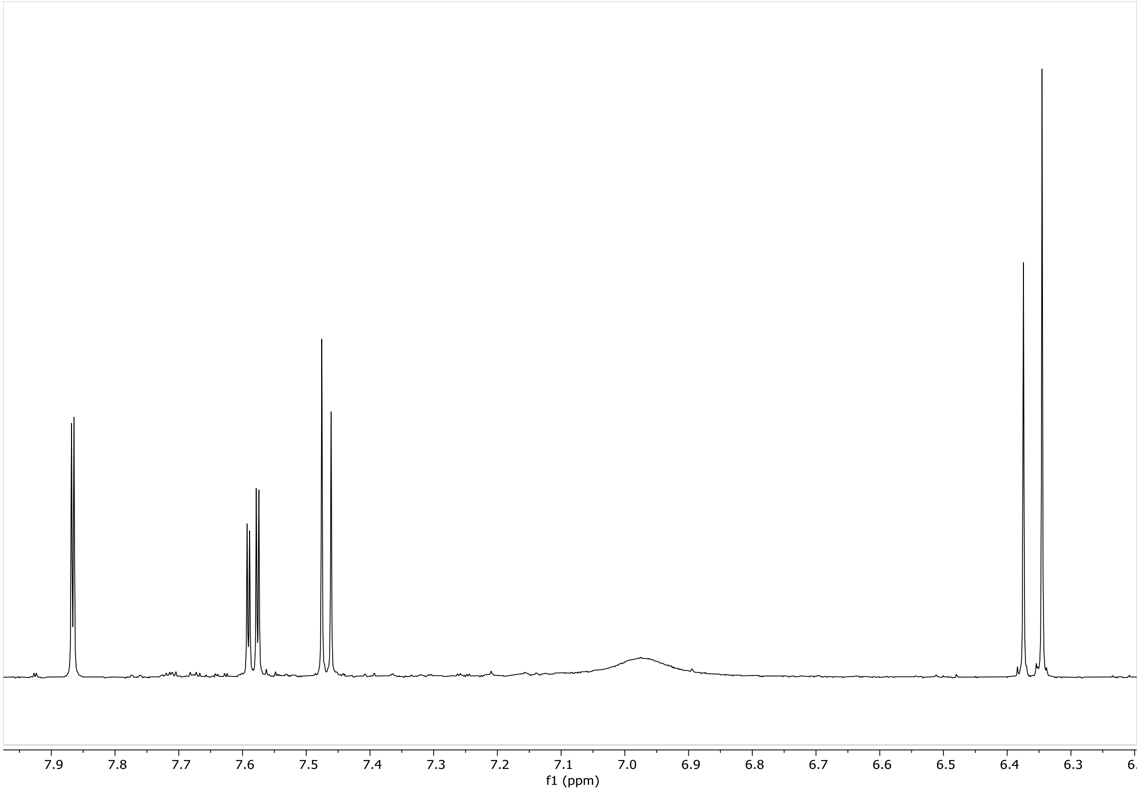

Figure S23 ^{1}H-NMR Spectrum of 2-amino-8-bromo-3H-phenoxazin-3-one

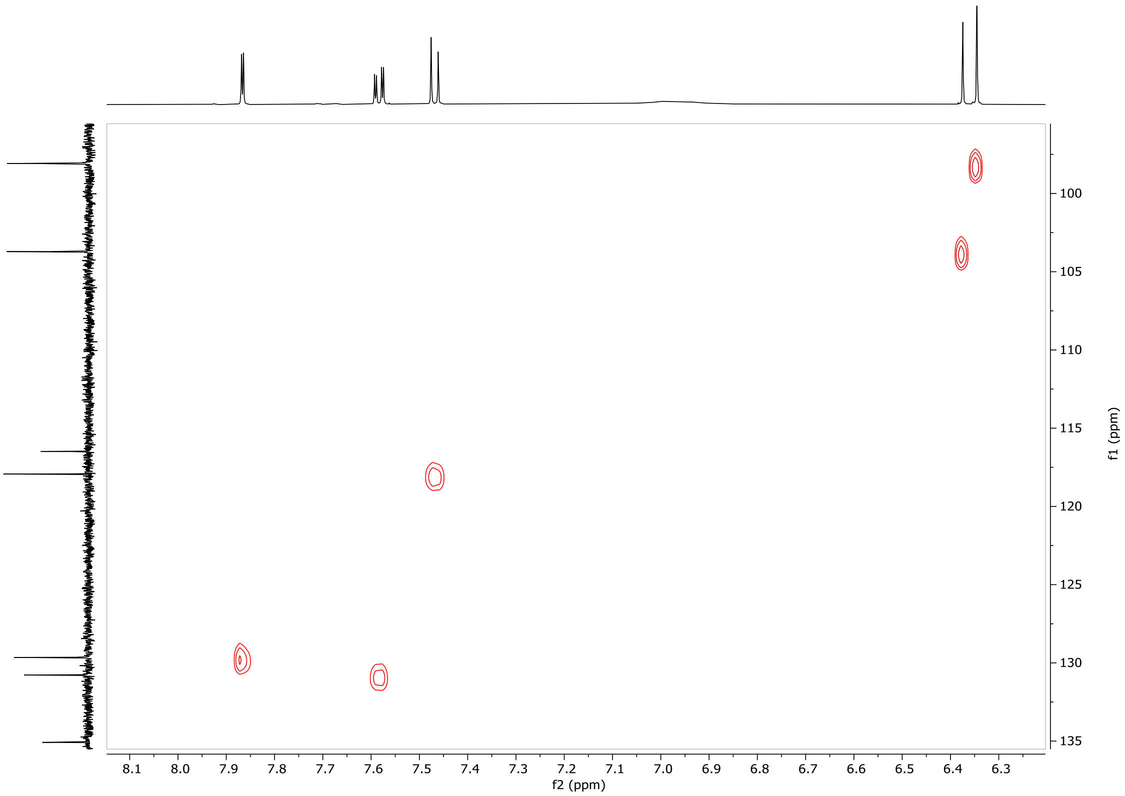

Figure S24 HSQC Spectrum of 2-amino-8-bromo-3H-phenoxazin-3-one

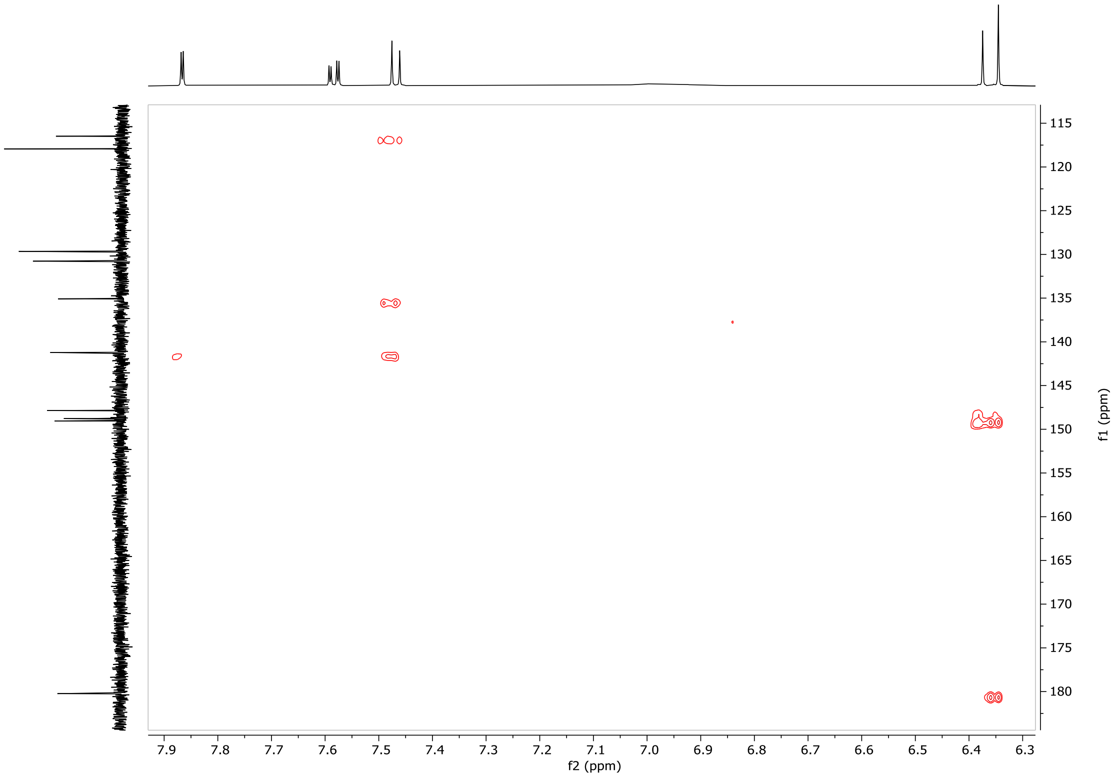

Figure S25 HMBC Spectrum of 2-amino-8-bromo-3H-phenoxazin-3-one

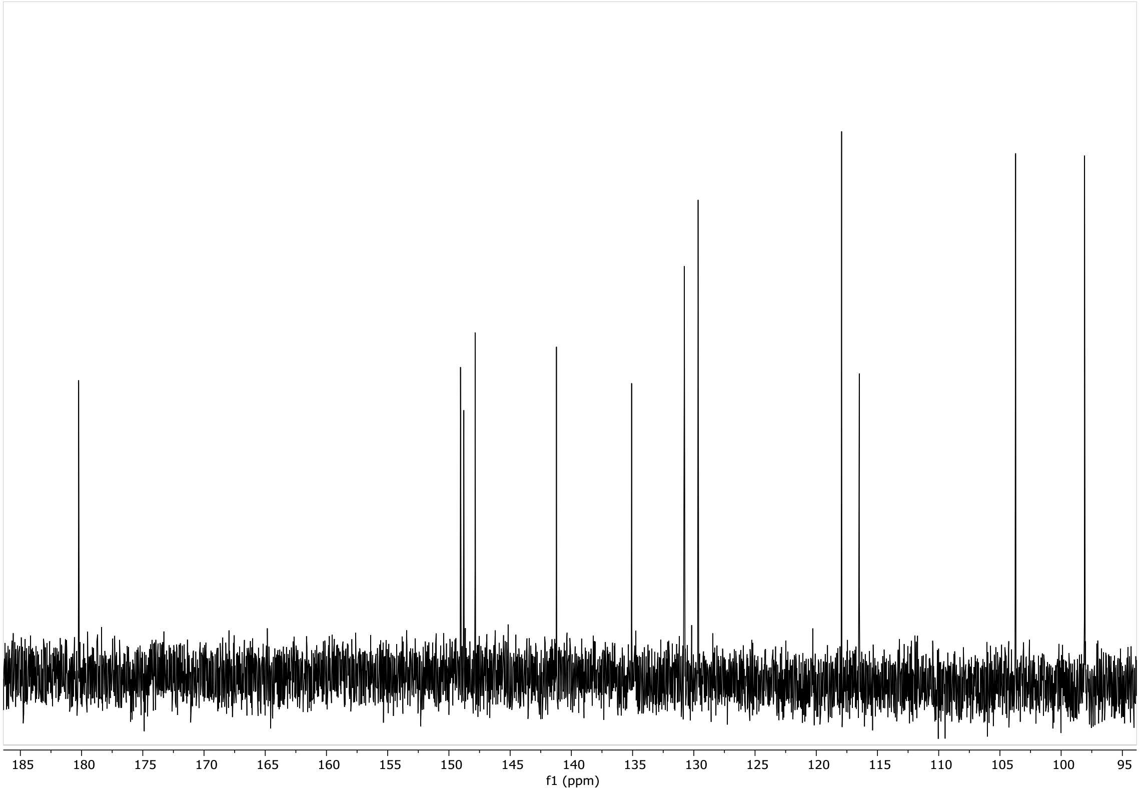

Figure S26 ^{13}C-NMR Spectrum of 2-amino-8-bromo-3H-phenoxazin-3-one

Structural characterization of 2-amino-8-bromo-4-iodo-3*H*-phenoxazin-3-one (**5**)

2-Amino-8-bromo-4-iodo-3*H*-phenoxazin-3-one (**5**), dark red powder, ^{1}H-NMR (DMSO-d_6, 600 MHz) δ (ppm) 7.92 (1H, d, *J*= 2.2 Hz, H-9), 7.63 (1H, dd, *J*= 8.8; 2.2 Hz, H-7), 7.55 (1H, d, *J*= 8.8 Hz, H-6); 7.10 (2H, bs, NH_2); 6.35 (1H, s, H-1); ^{13}C-NMR (DMSO-d_6, 150 MHz) δ (ppm) 176.2 (C, C-2), 149.8 (C, C-2), 148.3 (C, C-4a), 146.3 (C, C-10a), 141.6 (C, C-5a), 135.3 (C, C-9a), 131.8 (C, C-8), 131.2 (CH, C-7), 129.5 (CH, C-9), 118.1 (CH, C-6), 98.1 (CH, C-1), 81.2 (C, C-4). HRESIMS *m/z* 416.8749 $[M+H]^+$ (calcd for $C_{12}H_7Br_1I_1N_2O_2$, 416.8736).

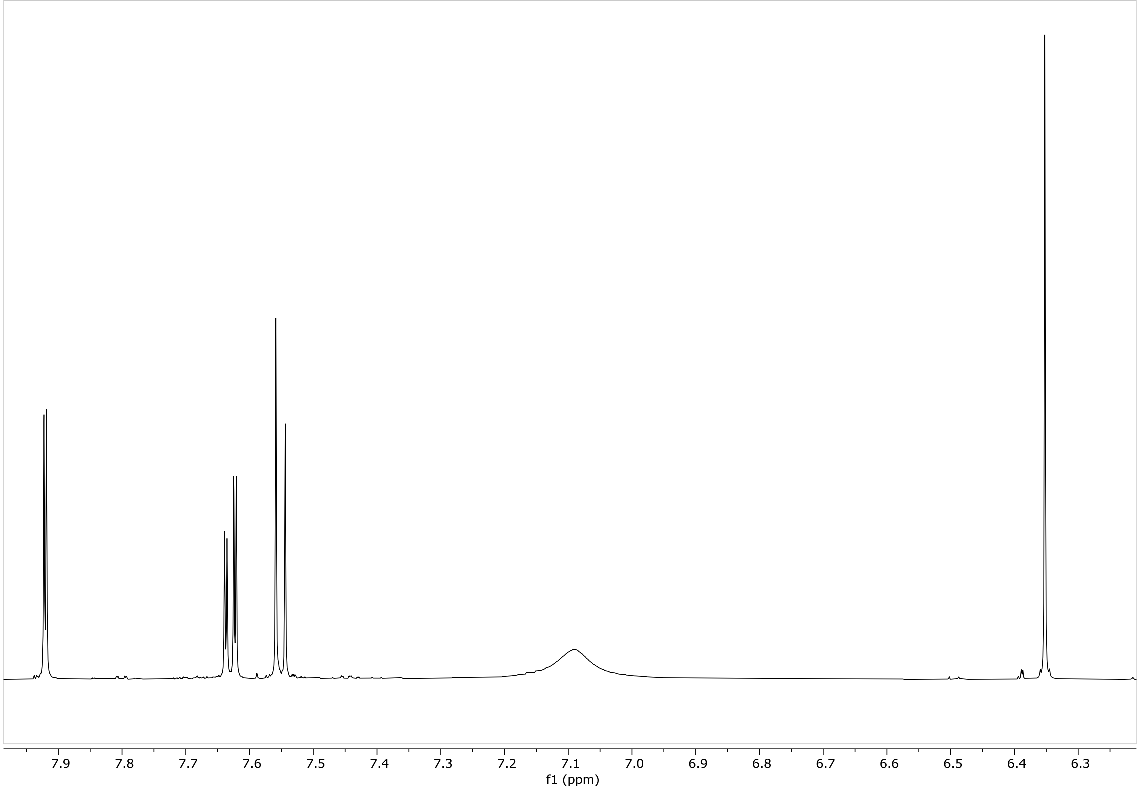

Figure S27 ^{1}H-NMR Spectrum of 2-amino-8-bromo-4-iodo-3H-phenoxazin-3-one

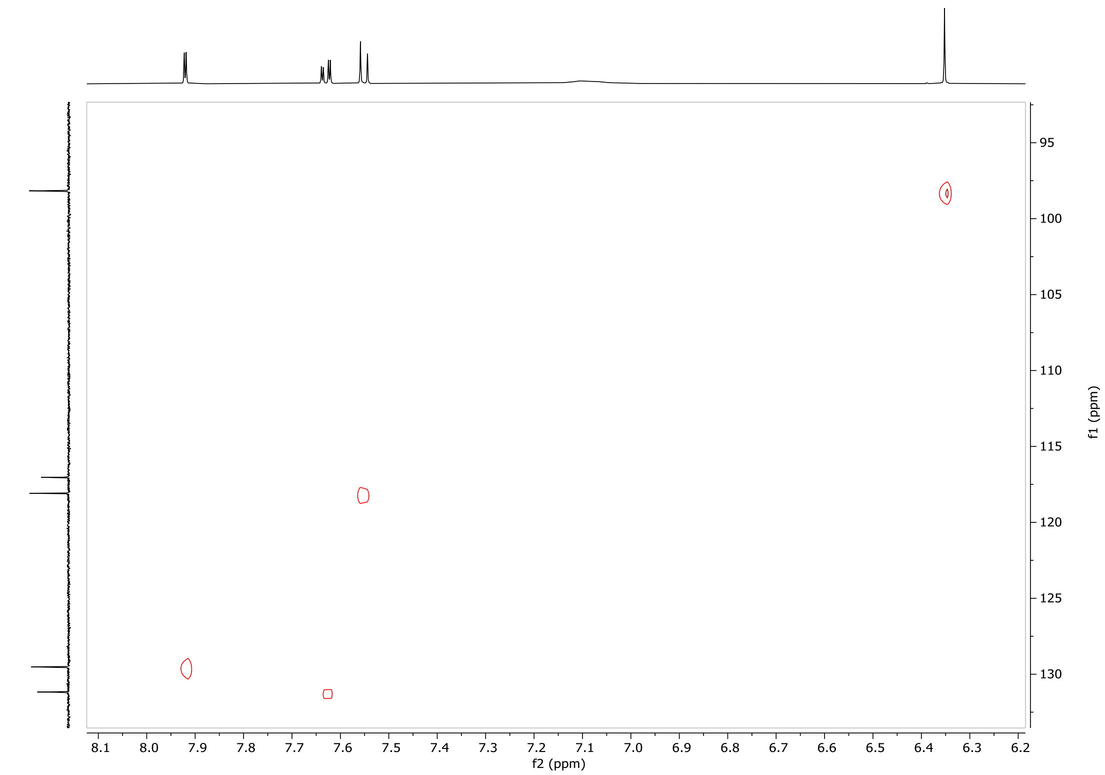

Figure S28 HSQC Spectrum of 2-amino-8-bromo-4-iodo-3H-phenoxazin-3-one

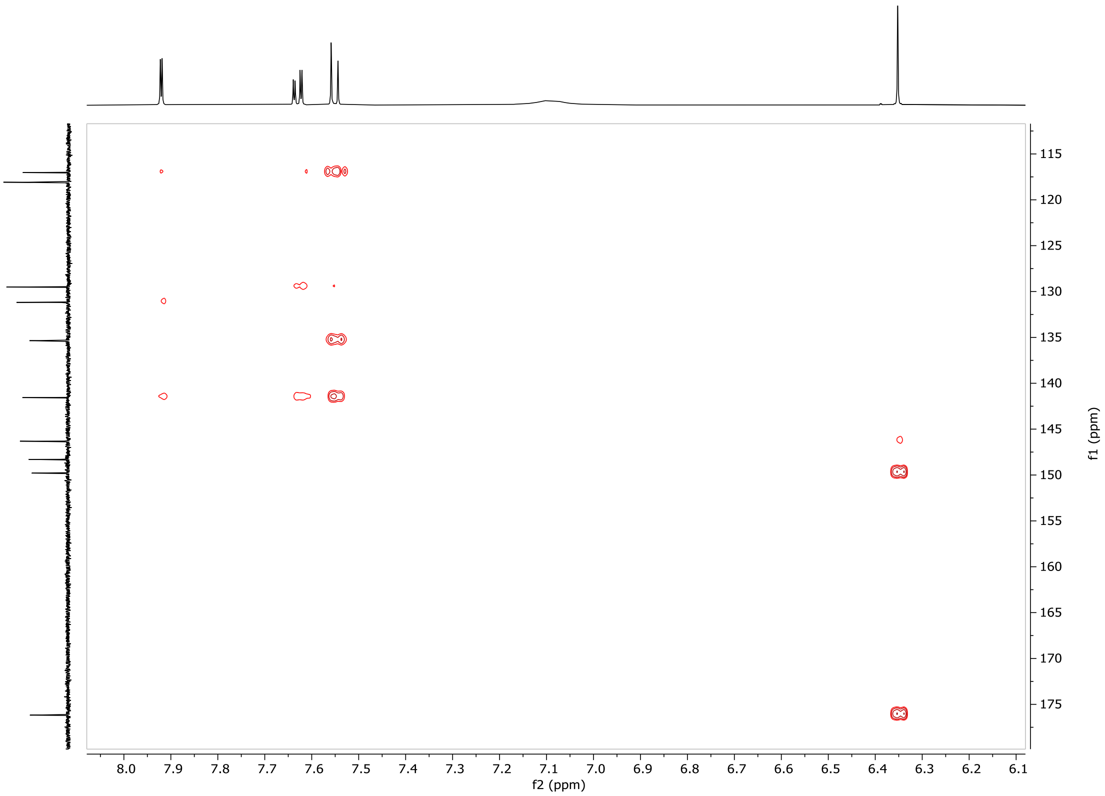

Figure S29 HMBC Spectrum of 2-amino-8-bromo-4-iodo-3H-phenoxazin-3-one

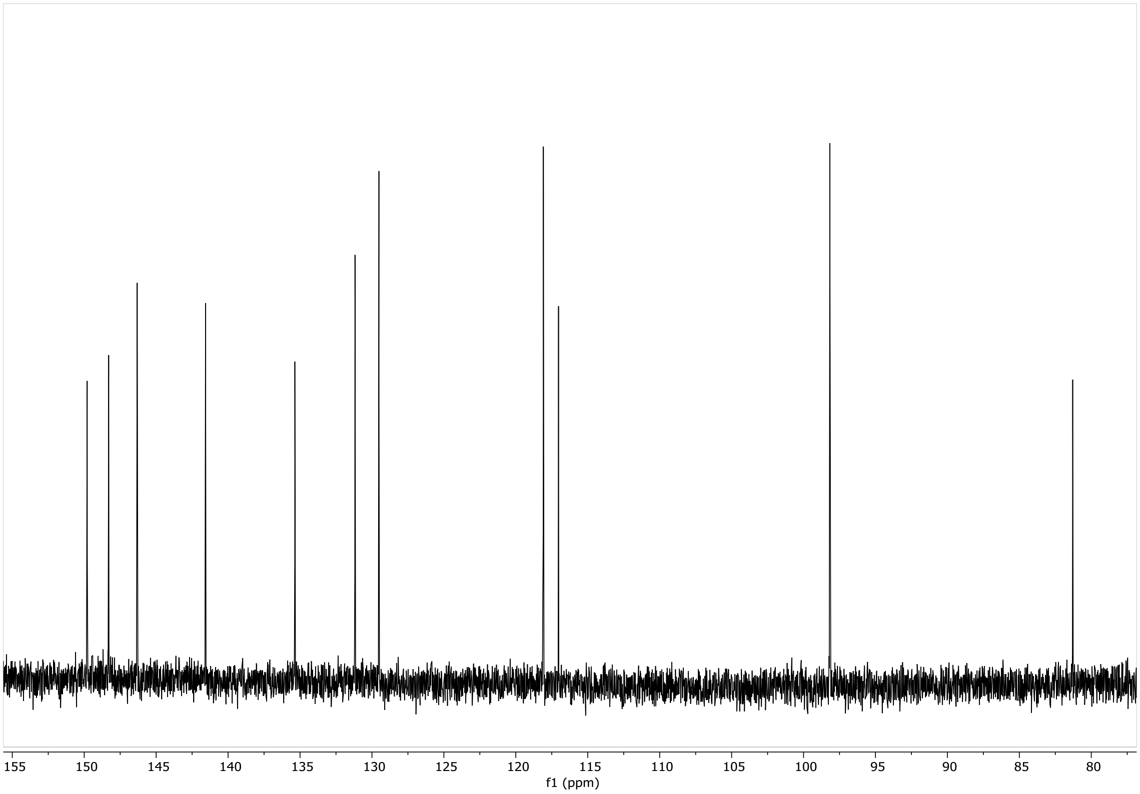

Figure S30 ^{13}C-NMR Spectrum of 2-amino-8-bromo-4-iodo-3H-phenoxazin-3-one

Structural characterization of 2-amino-4-iodo-3*H*-phenoxazin-3-one (**6**)

2-Amino-4-iodo-3*H*-phenoxazin-3-one (**6**), dark red powder, ^{1}H-NMR (DMSO-d_6, 600 MHz) δ (ppm) 7.77 (1H, dd, *J*= 8.0; 1.4 Hz, H-9), 7.60 (1H, dd, *J*= 8.3; 1.3 Hz, H-1), 7.53 (1H, ddd, *J*= 7.4; 8.0; 1.5 Hz, H-7), 7.45 (1H, ddd, *J*= 7.4; 8.0; 1.5 Hz, H-8), 6.92 (2H, bs, NH_2), 6.38 (1H, s, H-1); ^{13}C-NMR (DMSO-d_6, 150 MHz) δ (ppm) 176.1 (C, C-3), 149.8 (C, C-2), 147.5 (C, C-4a), 145.8 (C, C-10a), 142.3 (C, C-5a), 134.0 (C, C-9a), 129.2 (CH, C-7), 127.8 (CH, C-9), 125.8 (CH, C-8), 116.1 (CH, C-6), 98.4 (CH, C-1), 80.9 (C, C-4). HRESIMS *m/z* 338.9652 [M+H]$^+$ (calcd for $C_{12}H_8I_1N_2O_2$, 338.9630).

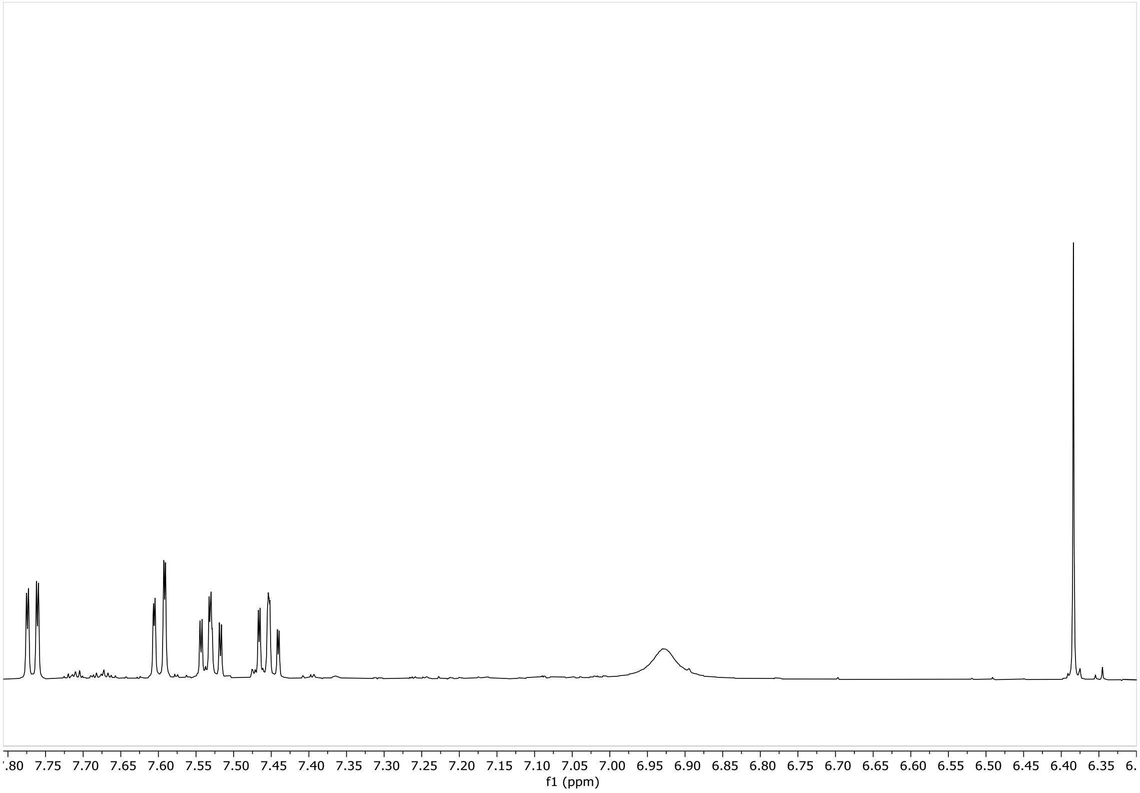

Figure S31 ^{1}H-NMR Spectrum of 2-amino-4-iodo-3H-phenoxazin-3-one

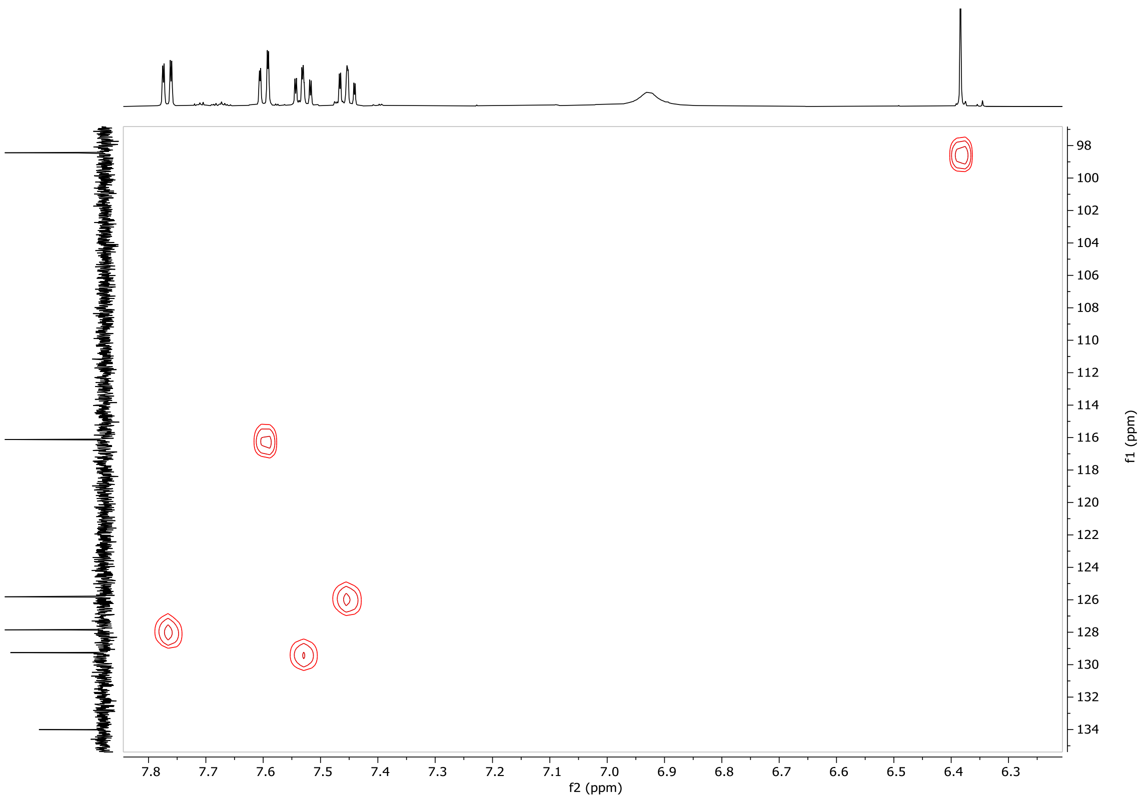

Figure S32 HSQC Spectrum of 2-amino-4-iodo-3H-phenoxazin-3-one

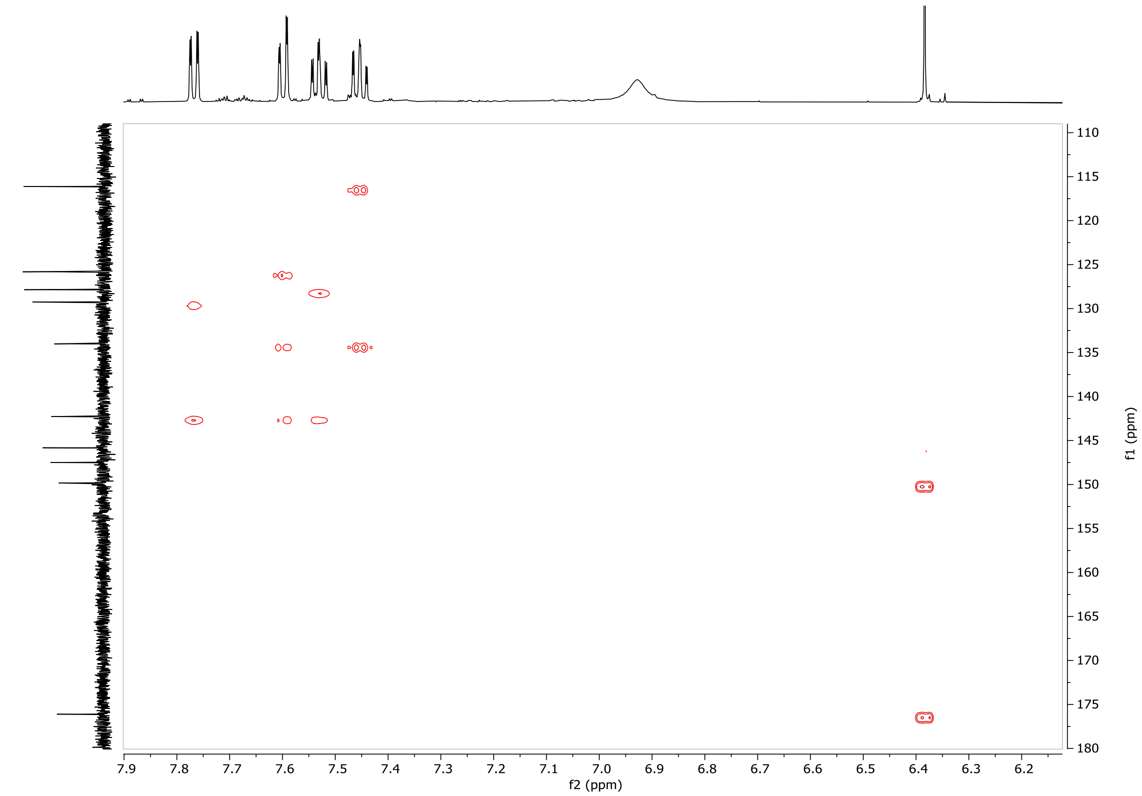

Figure S33 HMBC Spectrum of 2-amino-4-iodo-3H-phenoxazin-3-one

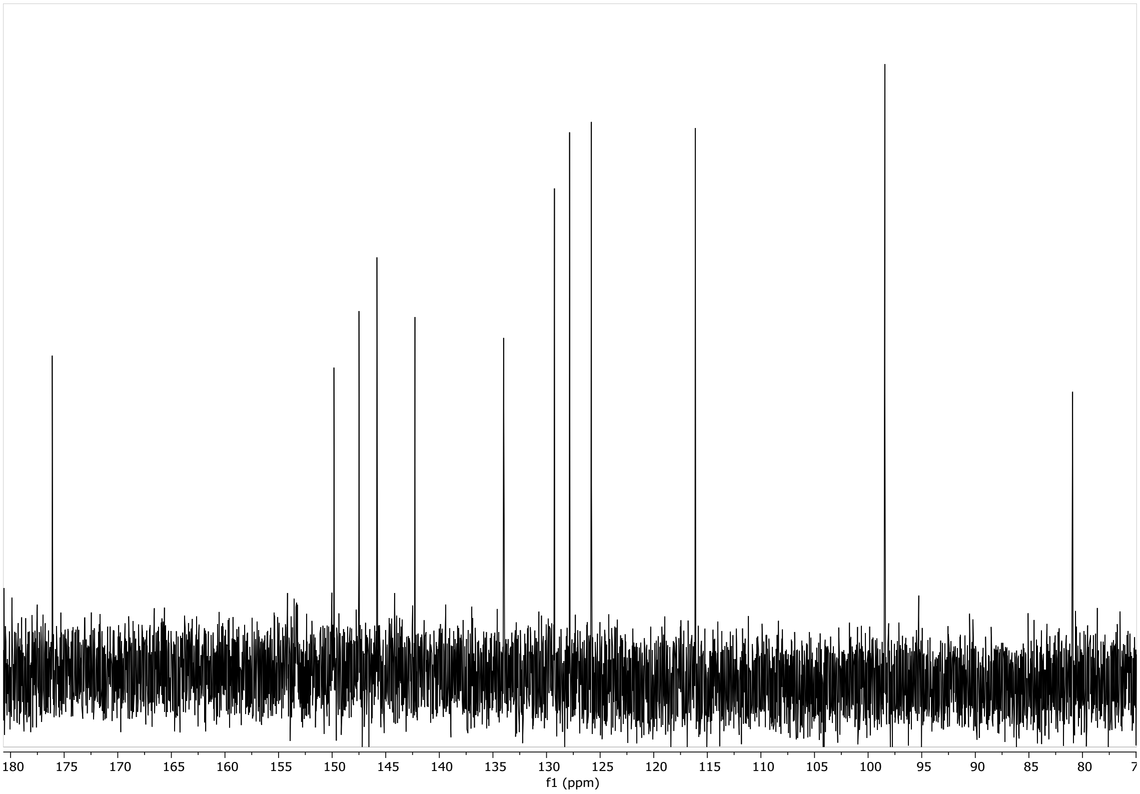

Figure S34 ^{13}C-NMR Spectrum of 2-amino-4-iodo-3H-phenoxazin-3-one

Structural characterization of 2-amino-3-oxo-3*H*-phenoxazine-8-carboxilyc acid (**7**)

2-Amino-3-oxo-3*H*-phenoxazine-8-carboxilyc acid (**7**), dark orange powder. ^{1}H-NMR (DMSO-d_6, 500 MHz) δ (ppm) 8.18 (1H, d, *J*= 2.1 Hz, H-9), 7.97 (1H, dd, *J*= 8.5; 2.0 Hz, H-7), 7.55 (1H, d, *J*= 8.5 Hz, H-6), 6.91 (2H, bs, NH_2), 6.41 (1H, s, H-4), 6.37 (1H, s, H-1); ^{13}C-NMR (DMSO-d_6, 125 MHz) δ (ppm) 180.4 (C, C-3), 167.1 (C, C-11), 149.0 (C, C-2), 148.7 (C, C-4a), 147.6 (C, C-10a), 144.6 (C, C-5a), 133.4 (C, C-9a), 129.2 (CH, C-7), 128.9 (CH, C-9), 127.6 (C, C-8), 116.2 (CH, C-6), 105.3 (CH, C-4), 98.3 (CH, C-1). HRESIMS *m/z* 255.0415 $[M-H]^-$ (calcd for $C_{13}H_7N_2O_4$, 255.0406).

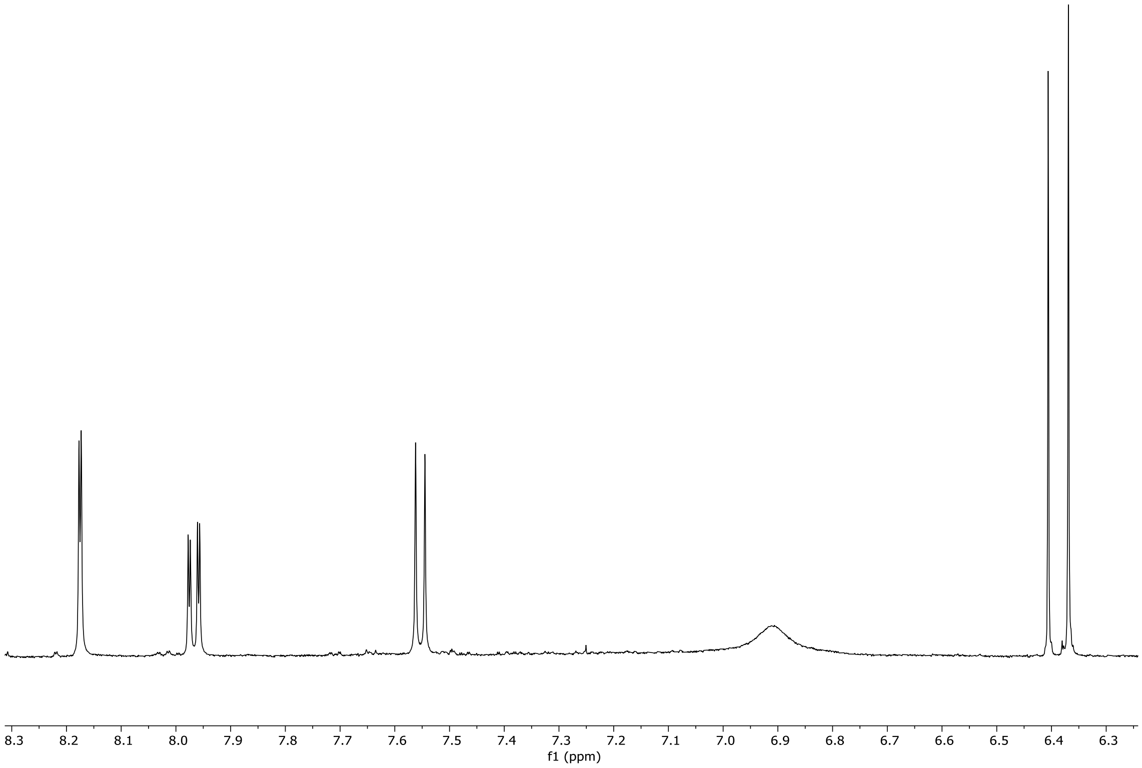

Figure S35 ^{1}H-NMR Spectrum of 2-amino-3-oxo-3H-phenoxazine-8-carboxilyc acid

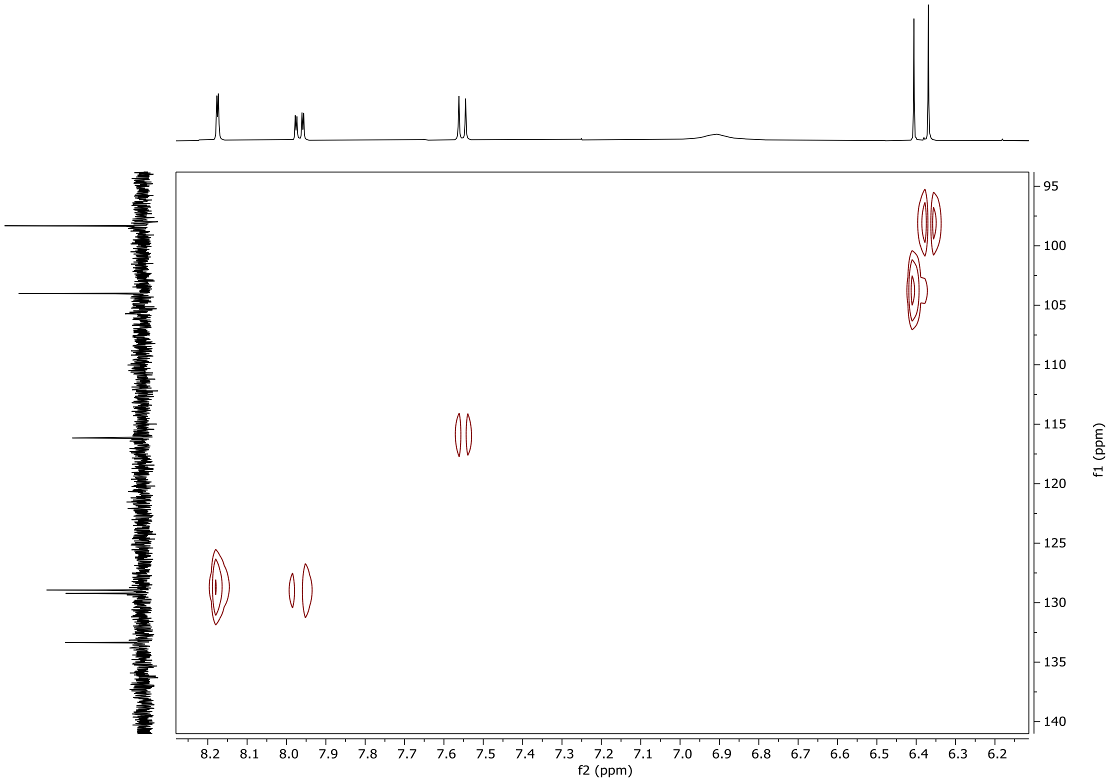

Figure S36. HSQC Spectrum of 2-amino-3-oxo-3H-phenoxazine-8-carboxilyc acid

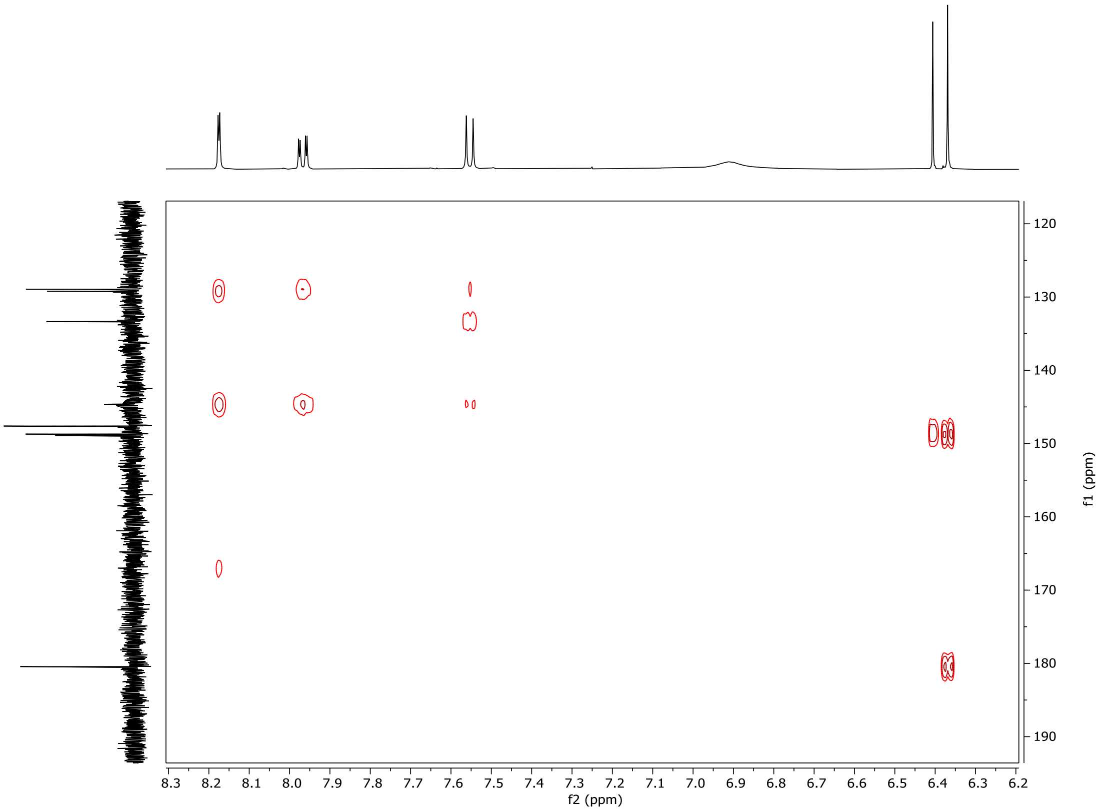

Figure S37. HMBC Spectrum of 2-amino-3-oxo-3H-phenoxazine-8-carboxilyc acid

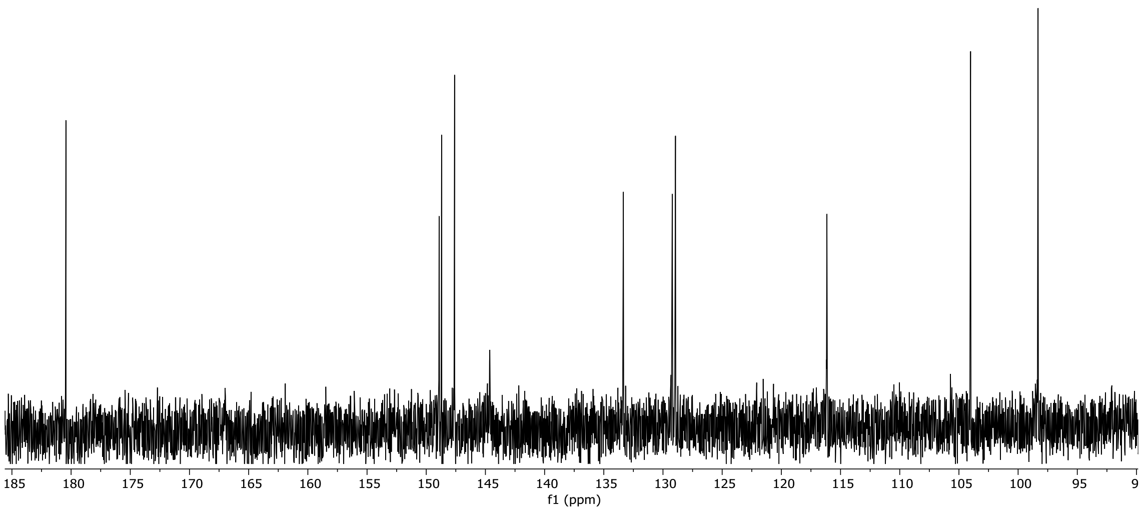

Figure S38. ^{13}C-NMR Spectrum of 2-amino-3-oxo-3H-phenoxazine-8-carboxilyc acid

Structural characterization of 2-amino-3-oxo-3*H*-phenoxazine-7-carboxilyc acid (**8**)

2-Amino-3-oxo-3*H*-phenoxazine-7-carboxilyc acid (**8**), dark orange powder, ^{1}H-NMR (DMSO-d_6, 500 MHz) δ (ppm) 7.91 (1H, d, *J*= 1.5 Hz, H-6), 7.88 (1H, dd, *J*= 1.5; 7.9 Hz, H-8), 7.75 (1H, dd, *J*= 0.9; 8.0 Hz, H-9), 7.01 (2H, bs, NH_2), 6.40 (1H, s, H-1), 6.38 (1H, s, H-4);^{13}C-NMR (DMSO-d_6, 125 MHz) δ (ppm) 180.7 (C, C-3), 166.1 (C, C-11), 150.1 (C, C-2), 149.6 (C, C-4a), 148.6 (C, C-10a), 141.9 (C, C-5a), 137.1 (C, C-9a), 131.6 (C, C-7), 128.2 (CH, C-6), 126.2 (CH, C-8), 117.1 (CH, C-9), 104.2 (CH, C-4), 98.7 (CH, C-1). HRESIMS *m/z* 255.0404 [M-H]$^{-}$ (calcd for $C_{13}H_9N_2O_4$, 255.0406).

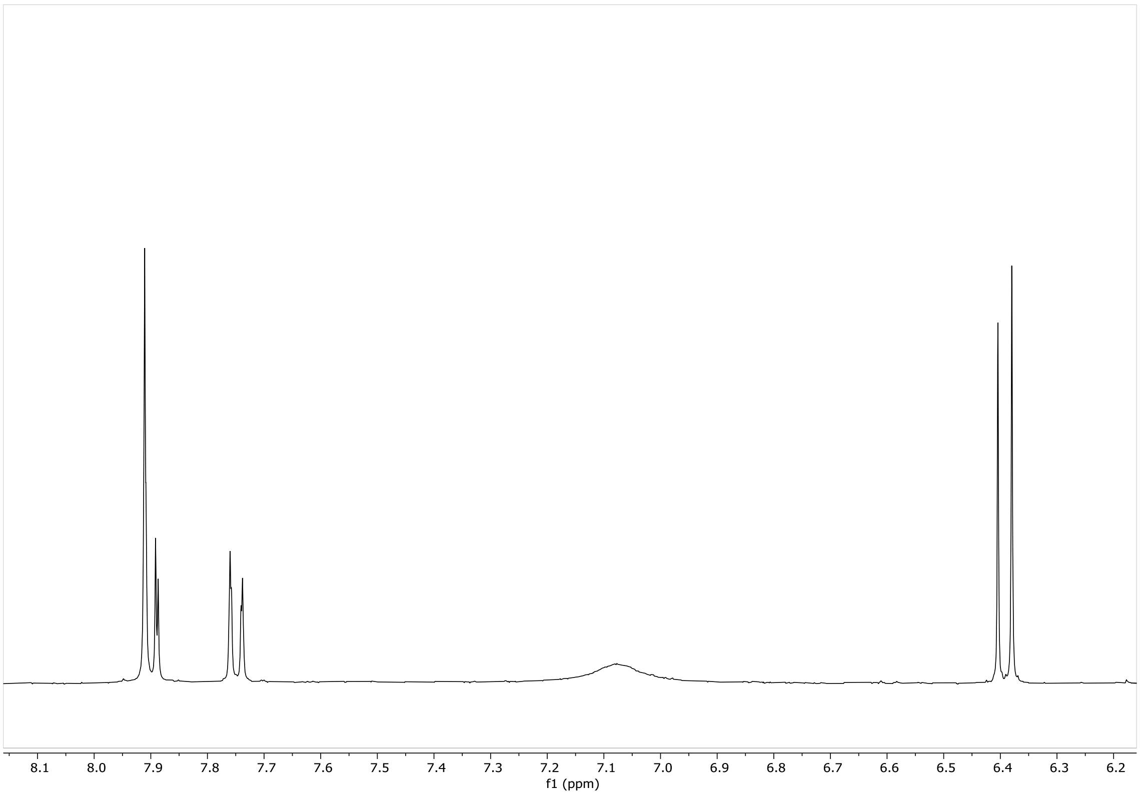

Figure S39. ^{1}H-NMR Spectrum of 2-amino-3-oxo-3H-phenoxazine-7-carboxilyc acid

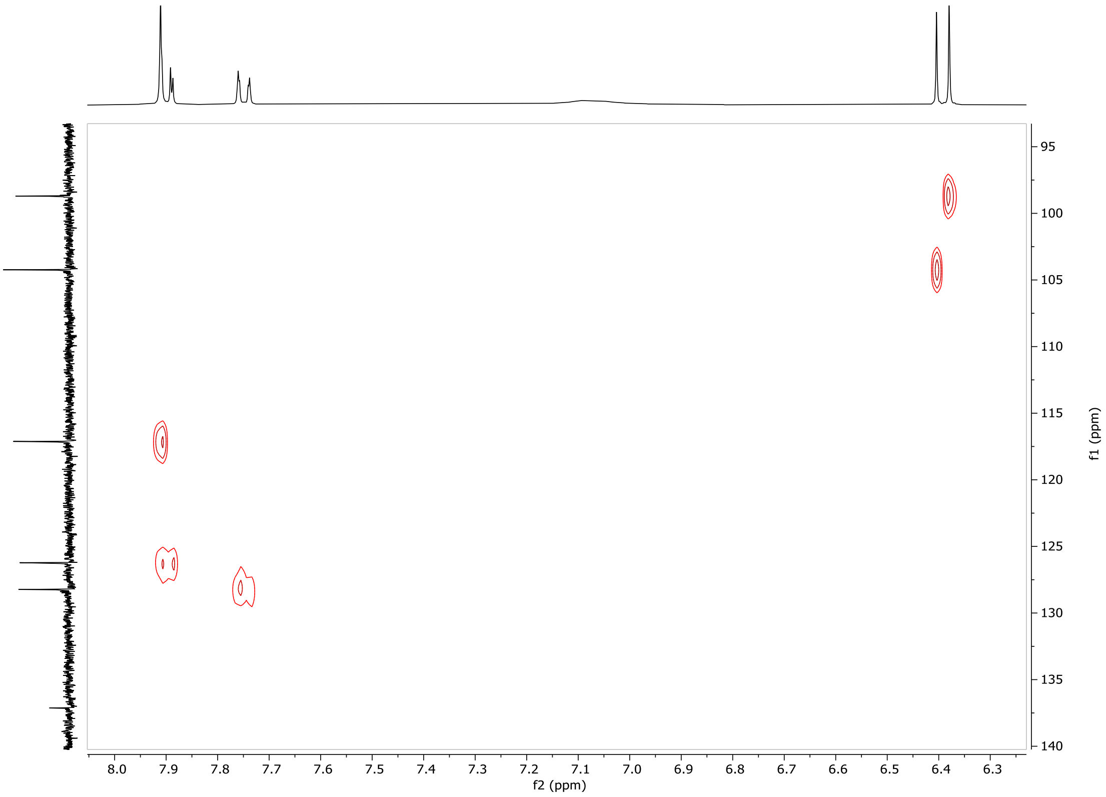

Figure S40. HSQC Spectrum of 2-amino-3-oxo-3H-phenoxazine-7-carboxilyc acid

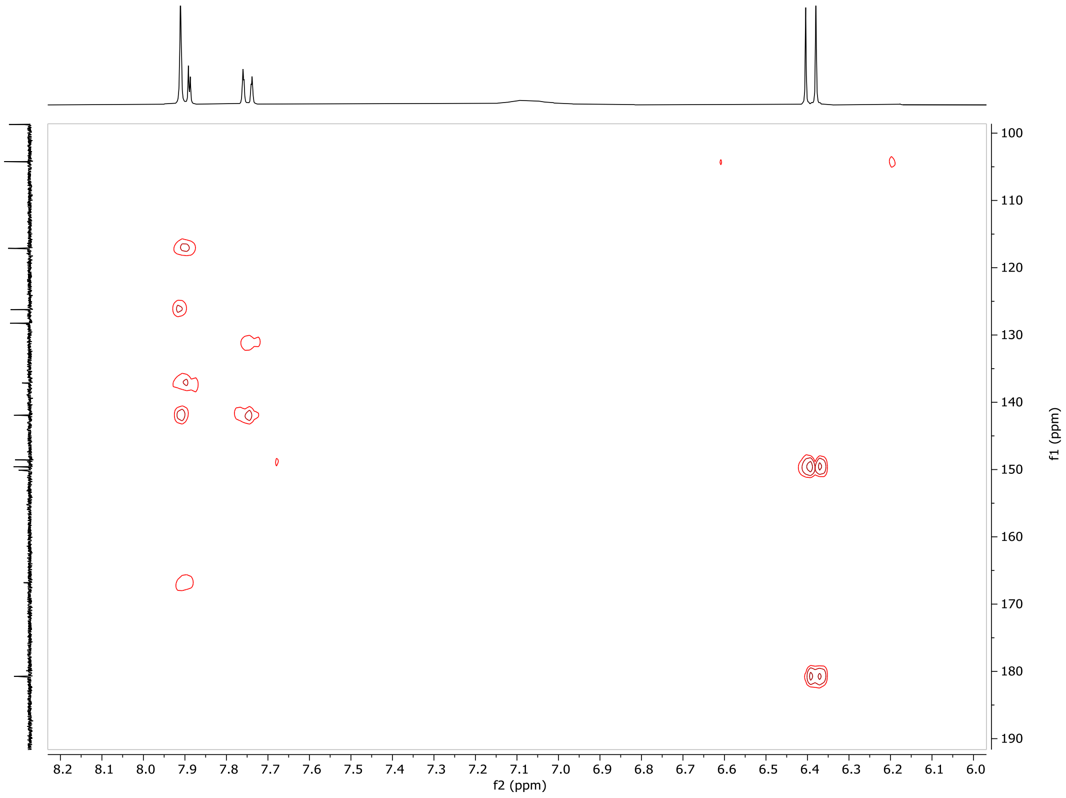

Figure S41. HMBC Spectrum of 2-amino-3-oxo-3H-phenoxazine-7-carboxilyc acid

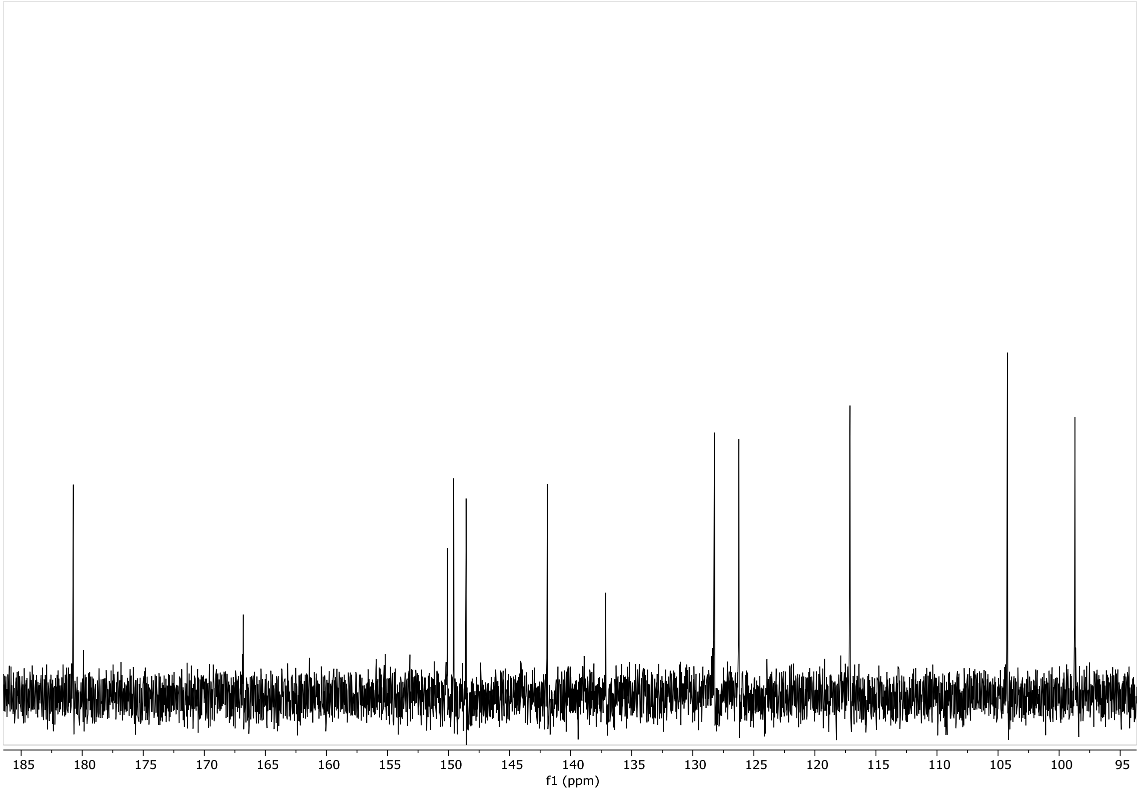

Figure S42. ^{13}C-NMR Spectrum of 2-amino-3-oxo-3H-phenoxazine-7-carboxilyc acid

Structural characterization of 2-amino-4-iodo-3-oxo-3*H*-phenoxazine-7-carboxilyc acid (**9**)

2-Amino-4-iodo-3-oxo-3*H*-phenoxazine-7-carboxilyc acid (**9**), dark orange powder. ^{1}H-NMR (DMSO-d_6, 500 MHz) δ (ppm) 7.94 (1H, d, *J*= 1.5 Hz, H-6), 7.92 (1H, dd, *J*= 8.3; 1.5 Hz, H-8), 7.84 (1H, d, *J*= 8.2 Hz, H-9), 7.00 (2H, bs, NH_2), 6.41 (1H, s, H-1); ^{13}C-NMR (DMSO-d_6, 125 MHz) δ (ppm) 177.3 (C, C-3), 166.5 (C, C-11), 150.4 (C, C-2), 150.1 (C, C-4a), 147.3 (C, C-10a), 142.1 (C, C-5a), 136.9 (C, C-9a), 131.6 (C, C-7), 128.7 (CH, C-9), 126.3 (CH, C-6), 116.8 (CH, C-8), 103.3 (CH, C-4), 73.9 (C, C-1). HRESIMS *m/z* 382.9535 $[M+H]^+$ (calcd for $C_{13}H_6I_1N_2O_4$, 382.9529).

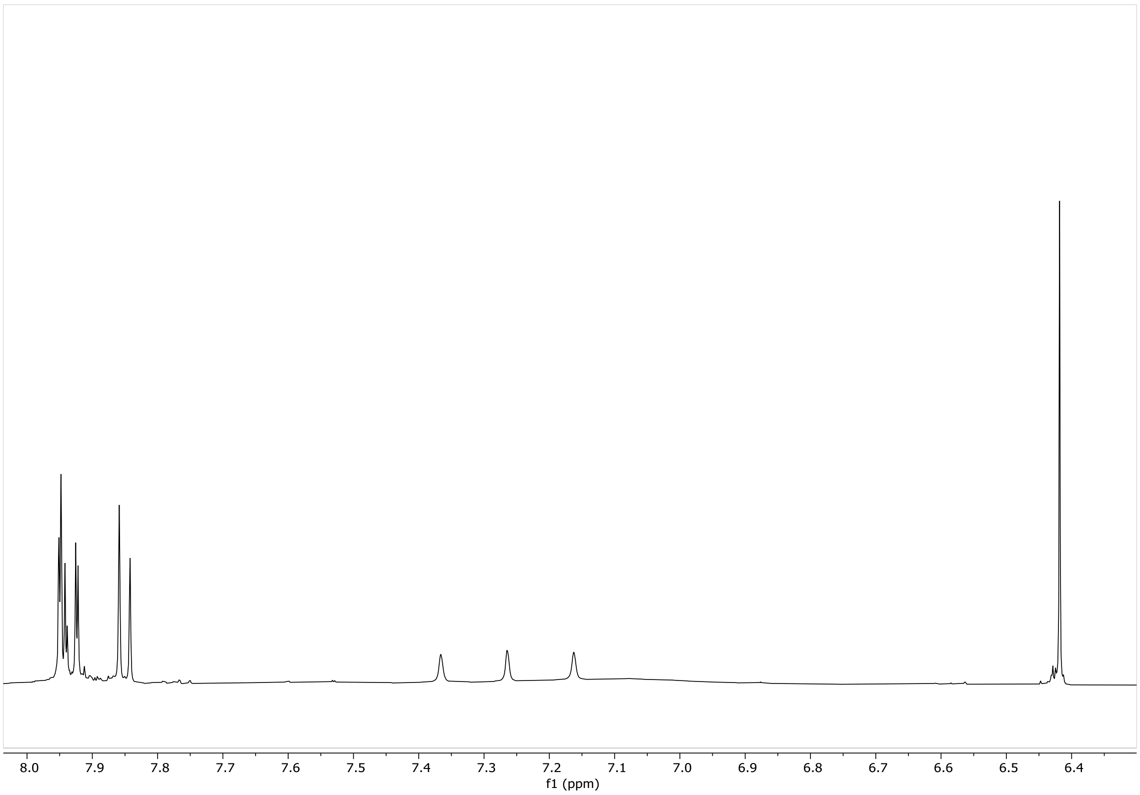

Figure S43. ^{1}H-NMR Spectrum of 2-amino-4-iodo-3-oxo-3H-phenoxazine-7-carboxilyc acid

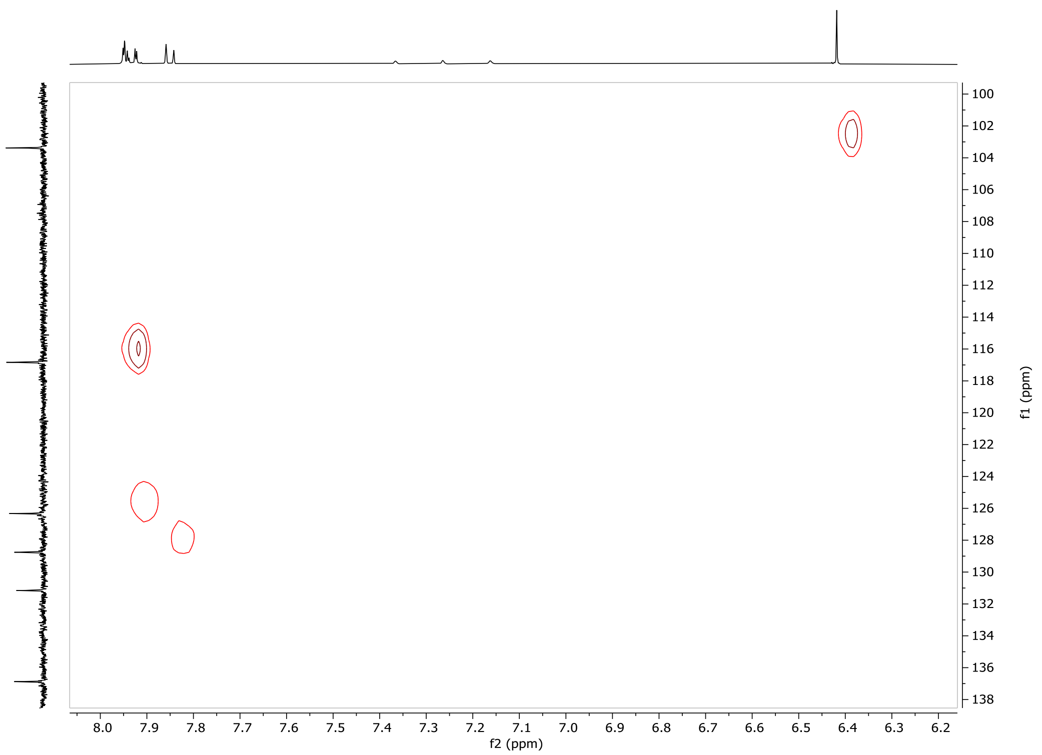

Figure S44. HSQC Spectrum of 2-amino-4-iodo-3-oxo-3H-phenoxazine-7-carboxilyc acid

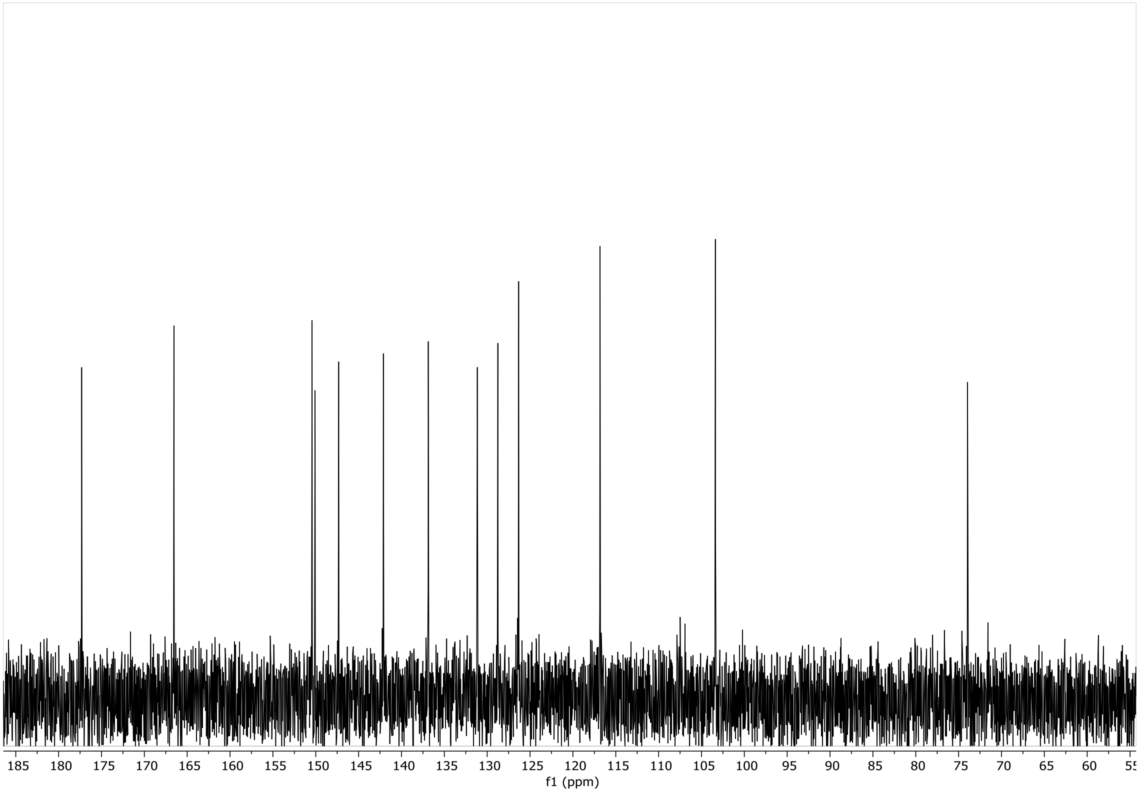

Figure S45. ^{13}C-NMR Spectrum of 2-amino-4-iodo-3-oxo-3H-phenoxazine-7-carboxilyc acid

Structural characterization of 2-amino-8-fluoro-4-iodo-3*H*-phenoxazin-3-one (10)

2-Amino-8-fluoro-4-iodo-3*H*-phenoxazin-3-one (**10**), dark yellow powder, ^{1}H-NMR (DMSO-d_6, 500 MHz) δ (ppm) 7.63 (1H, dd, *J*= 9.0; 4.3 Hz, H-6), 7.58 (1H, dd, *J*= 9.3; 2.7 Hz, H-9), 7.37 (1H, ddd, *J*= 8.5; 2.7 Hz, H-7), 7.1 (2H, bs, NH_2), 6.37 (1H, s, H-1); ^{13}C-NMR (DMSO-d_6, 125 MHz) δ (ppm) 176.1 (C, C-3), 158.9 (C, J_{C-F}= 242.0 Hz, C-8), 150.0 (C, C-2), 148.2 (C, C-4a), 146.3 (C, C-10a), 138.9 (C, C-5a), 134.8 (C, J_{C-F}= 13.0 Hz, C-9a), 117.5 (CH, J_{C-F}= 9.7 Hz, C-6), 116.1 (CH, J_{C-F}= 24.7 Hz, C-7), 112.8 (CH, J_{C-F}= 23.5 Hz, C-9), 98.0 (CH, C-1), 80.8 (C, C-4). HRESIMS *m/z* 354.9371 $[M-H]^-$ (calcd for $C_{12}H_5F_1I_1N_2O_2$, 354.9380).

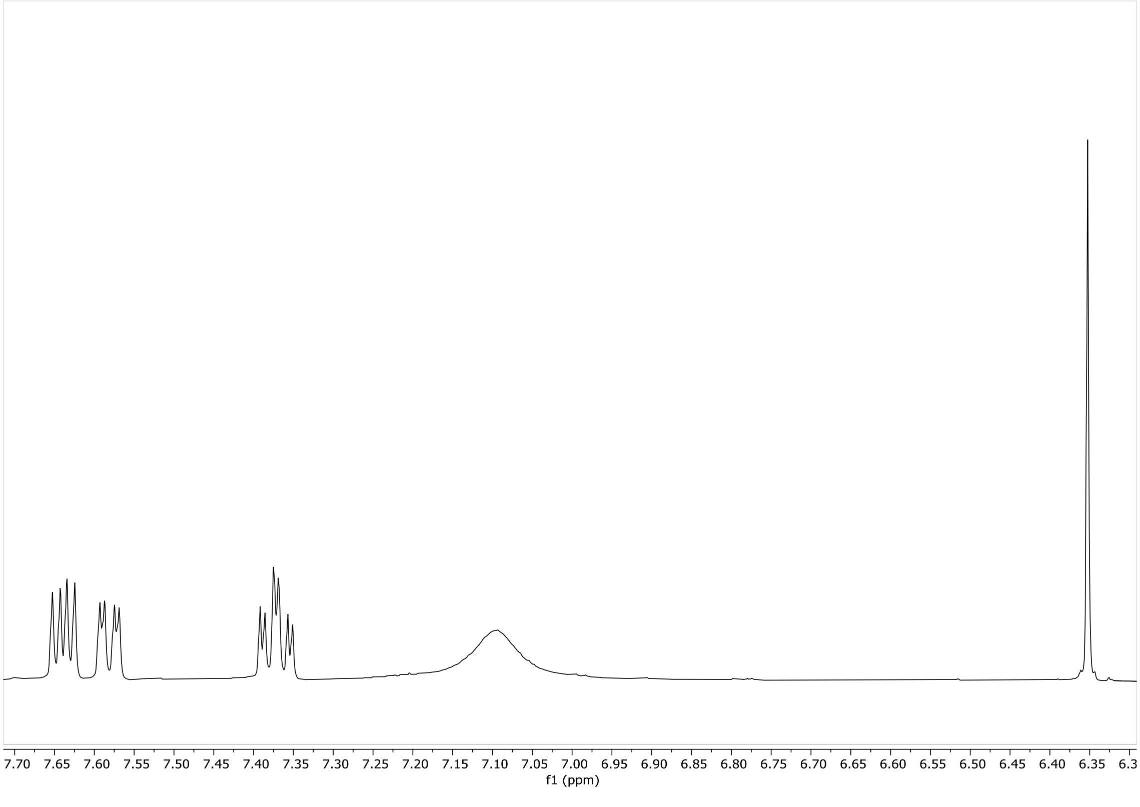

Figure S46. ^{1}H-NMR Spectrum of 2-amino-8-fluoro-4-iodo-3H-phenoxazin-3-one

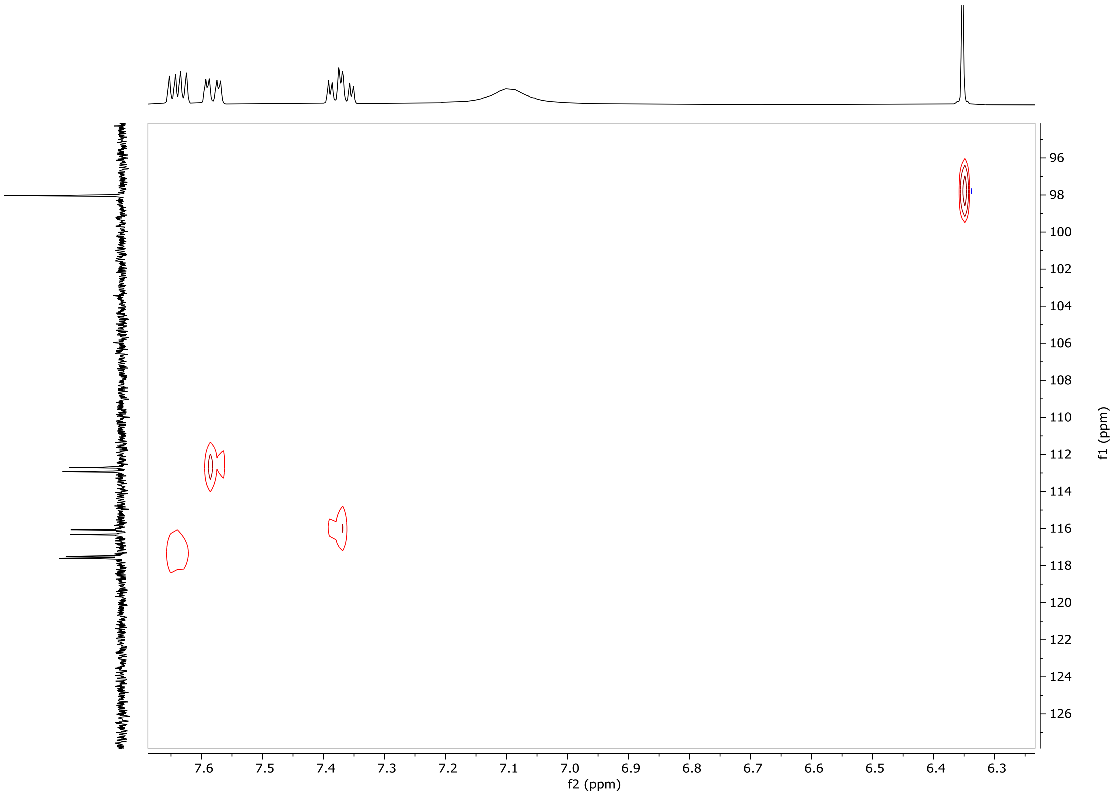

Figure S47. HSQC Spectrum of 2-amino-8-fluoro-4-iodo-3H-phenoxazin-3-one

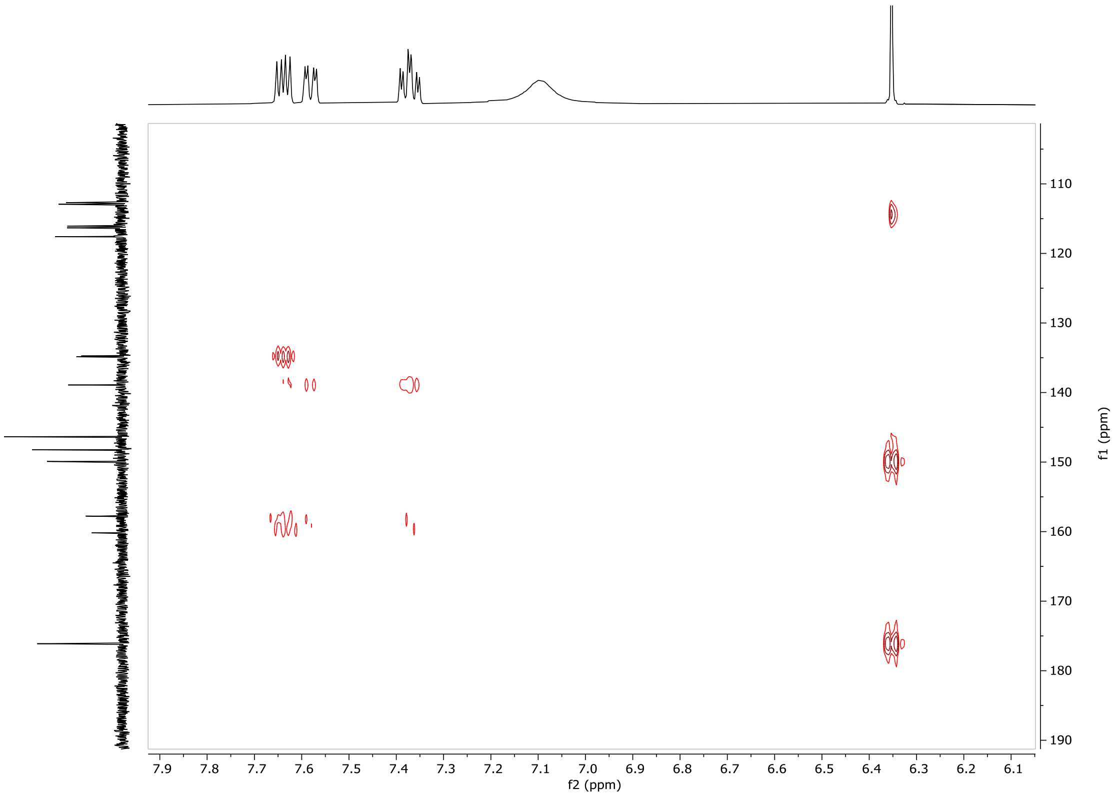

Figure S48. HMBC Spectrum of 2-amino-8-fluoro-4-iodo-3H-phenoxazin-3-one

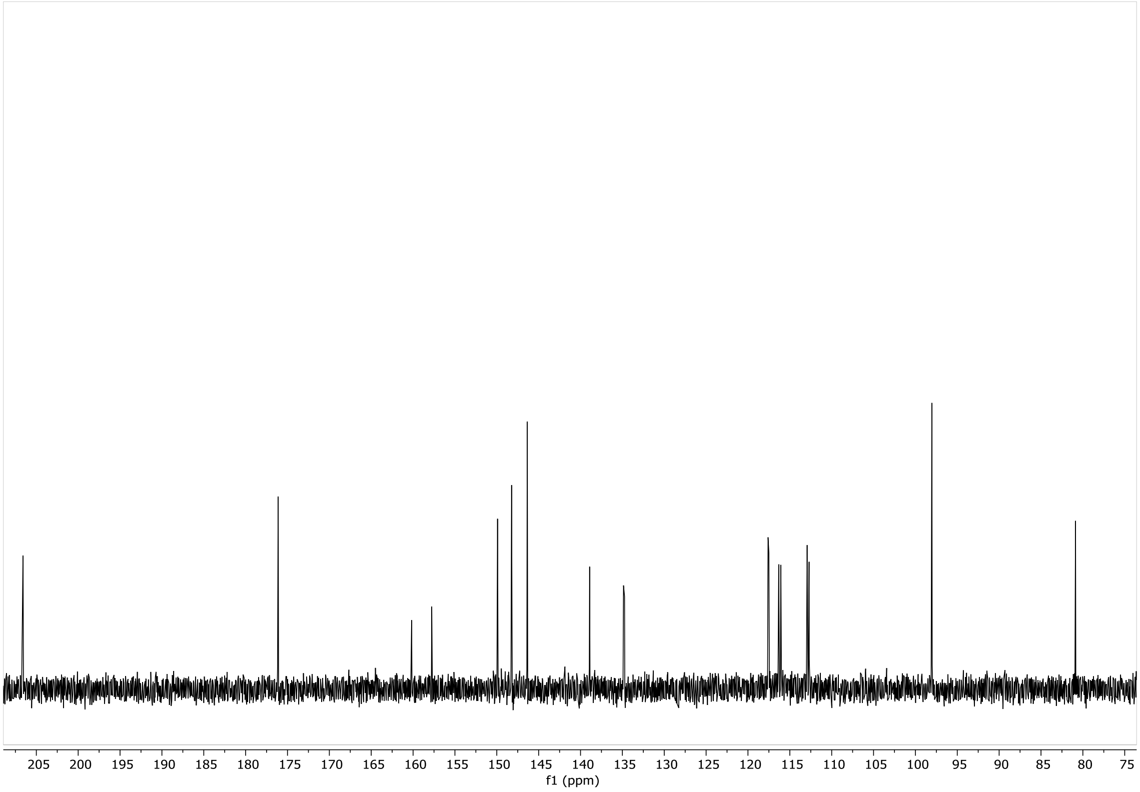

Figure S49. ^{13}C-NMR spectrum of 2-amino-8-fluoro-4-iodo-3H-phenoxazin-3-one

Structural characterization of 2-amino-3*H*-dipyrido[3,2-b:2′,3′-e][1,4]oxazin-3-one (**11**)

2-Amino-3*H*-dipyrido[3,2-*b*:2′,3′-*e*][1,4]oxazin-3-one (**11**), dark yellow powder, ^{1}H-NMR (DMSO-d_6, 400 MHz) δ (ppm) 8.54 (1H, d, J=8.8 Hz, H-8), 8.28 (2H, bs, NH2), 7.95 (1H, d, J=8.2 Hz, H-6), 7.45 (1H, dd, J=8.8; 3.8 Hz, H-7), 6.56 (1H, s, H-4); ^{13}C-NMR (DMSO-d_6, 100 MHz) δ (ppm) 176.3 (C, C-3), 158.1 (C, C-2), 153.0 (C, C-10a), 151.0 (C, C-4a), 146.8 (C-H, C-8), 145.8 (C, C-9a), 138.6 (C, C-5a), 124.1 (C-H, C-6), 123.5 (CH, C-7), 105.3 (C-H, C-4). HRESIMS m/z 215.0571 $[M+H]^+$ (calcd for $C_{10}H_6N_4O_2$, 215.0569).

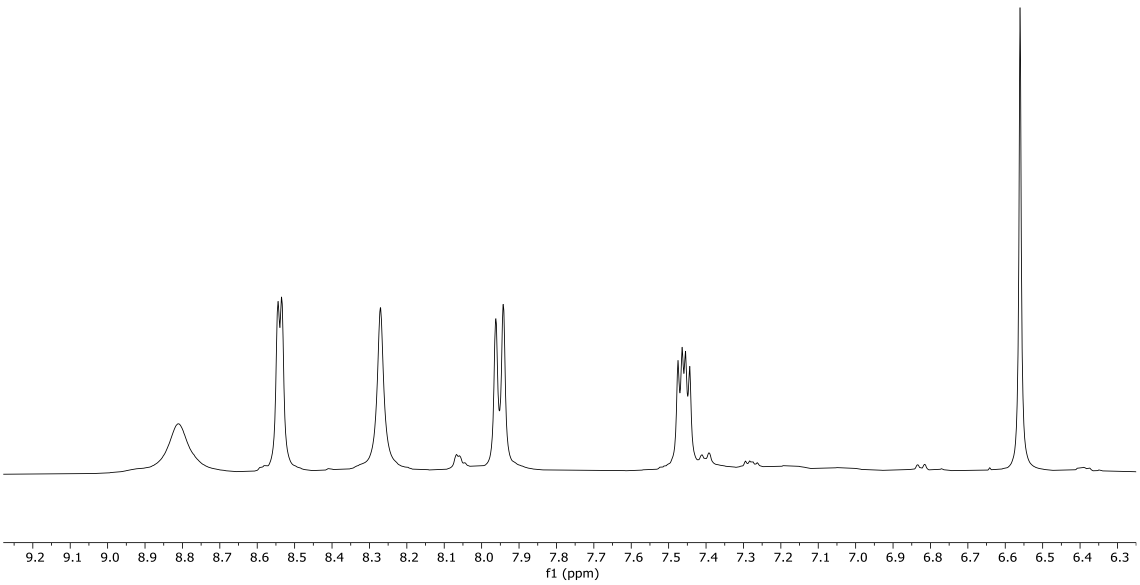

Figure S50. ^{1}H-NMR Spectrum of 2-amino-3H-dipyrido[3,2-b:2’,3’-e][1,4]oxazin-3-one

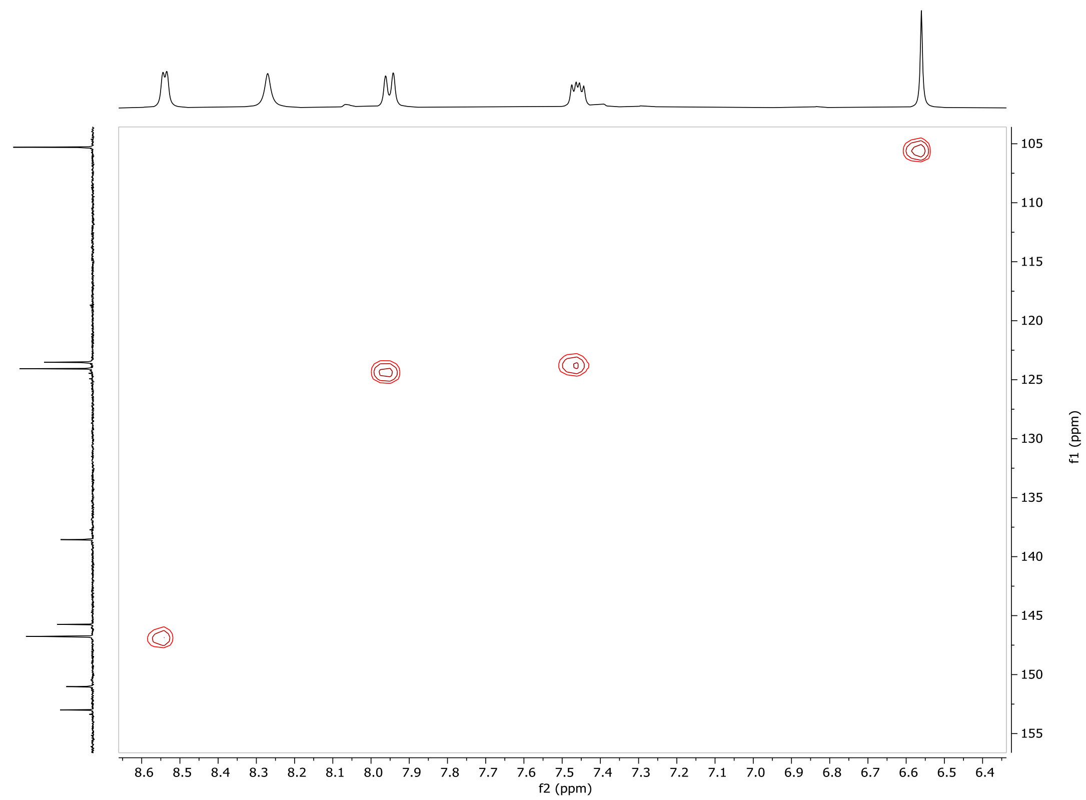

Figure S51. HSQC Spectrum of 2-amino-3H-dipyrido[3,2-b:2′,3′-e][1,4]oxazin-3-one

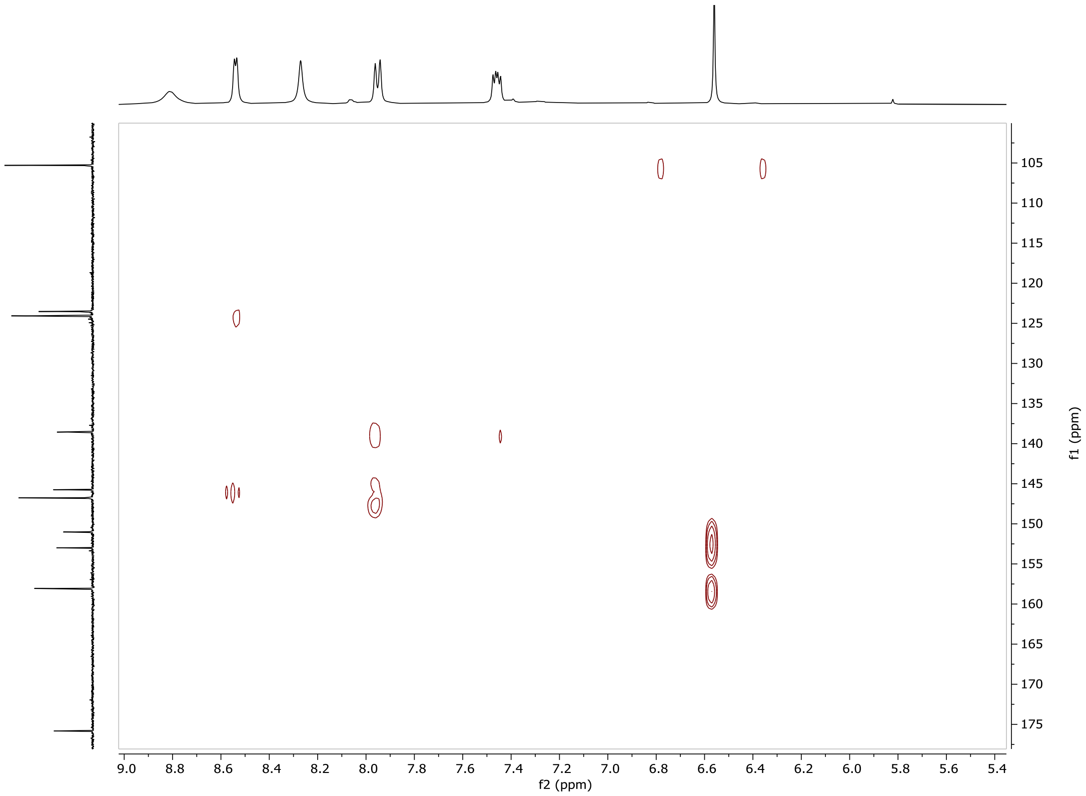

Figure S52. HMBC Spectrum of 2-amino-3H-dipyrido[3,2-b:2′,3′-e][1,4]oxazin-3-one

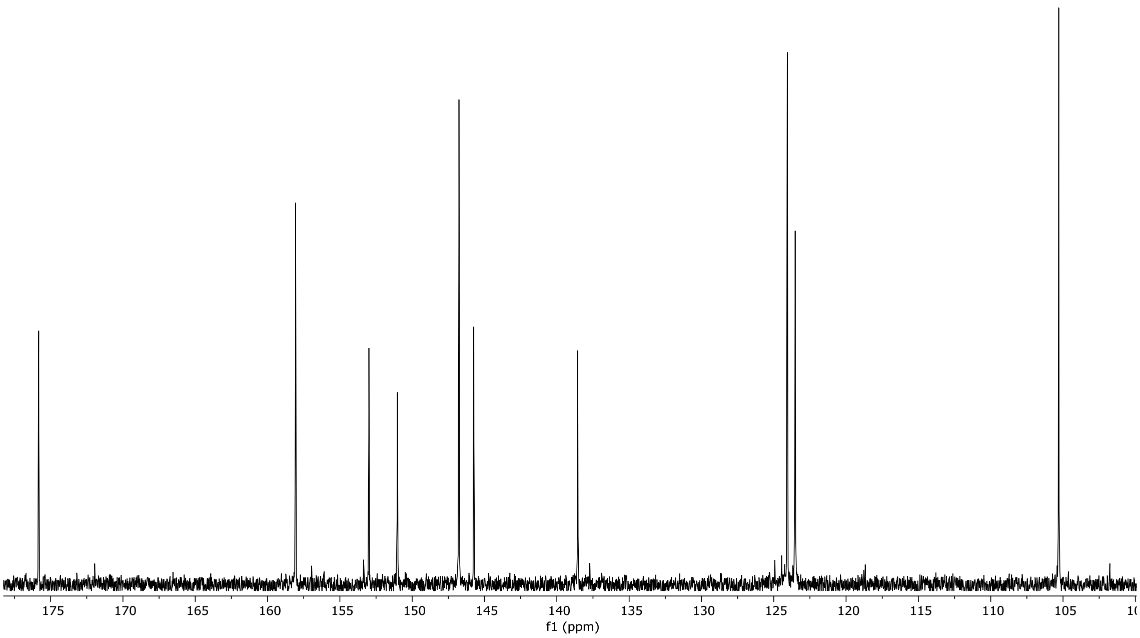

Figure S53. ^{13}C-NMR Spectrum of 2-amino-3H-dipyrido[3,2-b:2’,3’-e][1,4]oxazin-3-one

Structural characterization of 2-amino-8-bromo-3*H*-benzo[b]pyrido[2,3-e][1,4]oxazin-3-one (**13**)

2-Amino-8-bromo-3*H*-benzo[b]pyrido[2,3-e][1,4]oxazin-3-one (**13**), ^{1}H-NMR (DMSO-d_6, 600 MHz) δ (ppm) 7.83 (1H, d, *J*= 2.5 Hz, H-9), 7.60 (1H, dd, *J*= 9.0; 2.5 Hz, H-7), 7.94 (1H, d, *J*= 9 Hz, H-6), 6.53 (1H, s, H-4); ^{13}C-NMR (DMSO-d_6, 150 MHz) δ (ppm) 176.0 (C, C-3), 158.4 (C, C-2), 151.6 (C, C-4a), 151.3 (C, C-10a), 141.7 (C, C-5a), 135.9 (C,C-9a), 131.3 (CH,C-7), 130.4 (CH, C-9), 118.5 (CH,C-6), 117.3 (C,C-8), 105.7 (CH,C-4). HRESIMS *m/z* 289.9568 $[M+H]^-$ (calcd for $C_{11}H_6BrN_3O_2$, 289.9565).

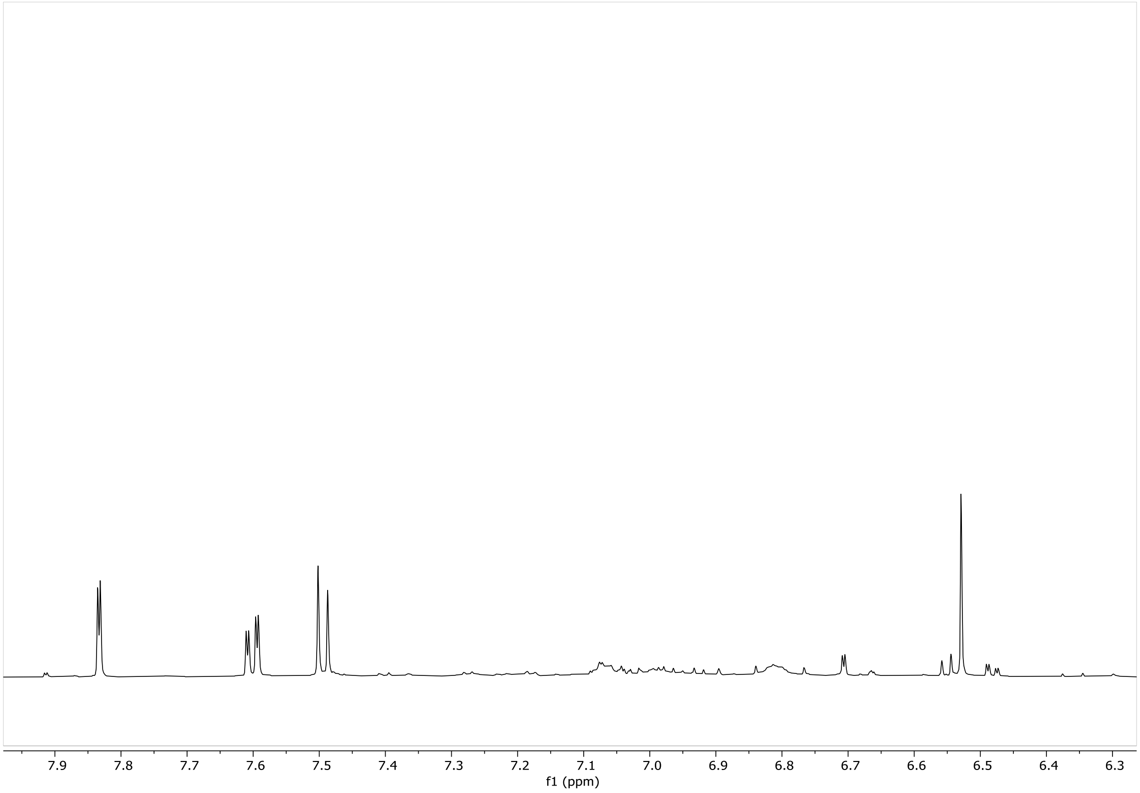

Figure S54. ^{1}H-NMR Spectrum of 2-amino-8-bromo-3H-benzo[b]pyrido[2,3-e][1,4]oxazin-3-one

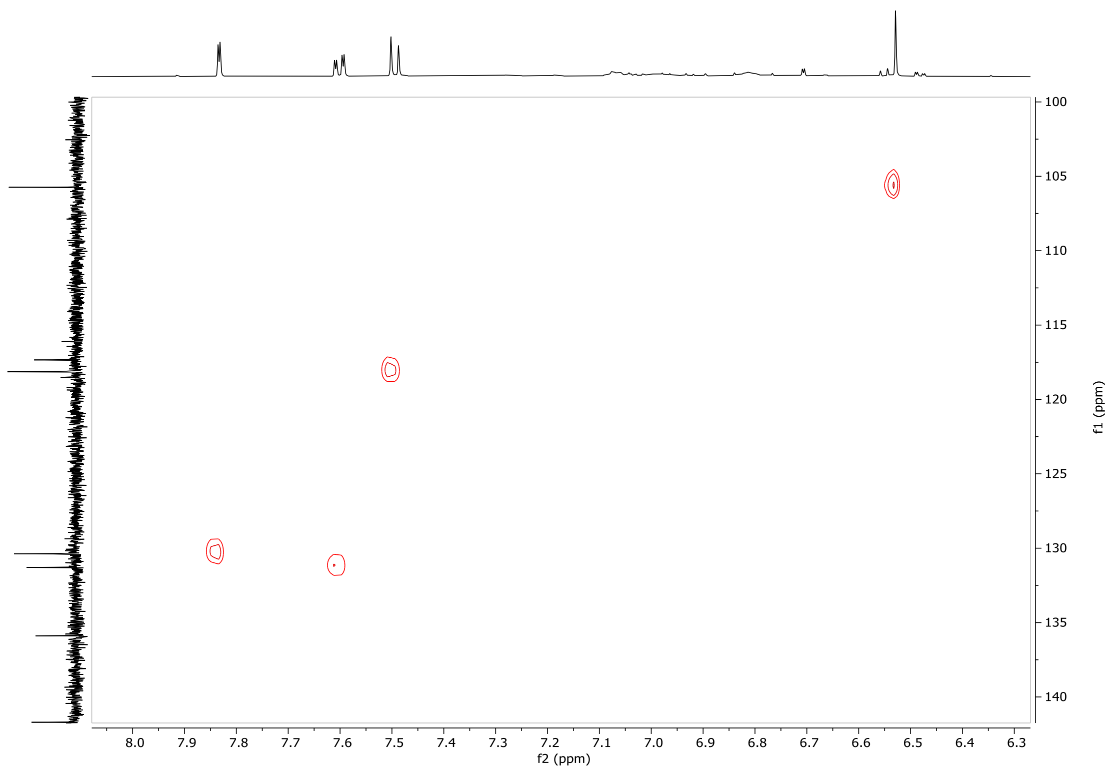

Figure S55. HSQC Spectrum of 2-amino-8-bromo-3H-benzo[b]pyrido[2,3-e][1,4]oxazin-3-one

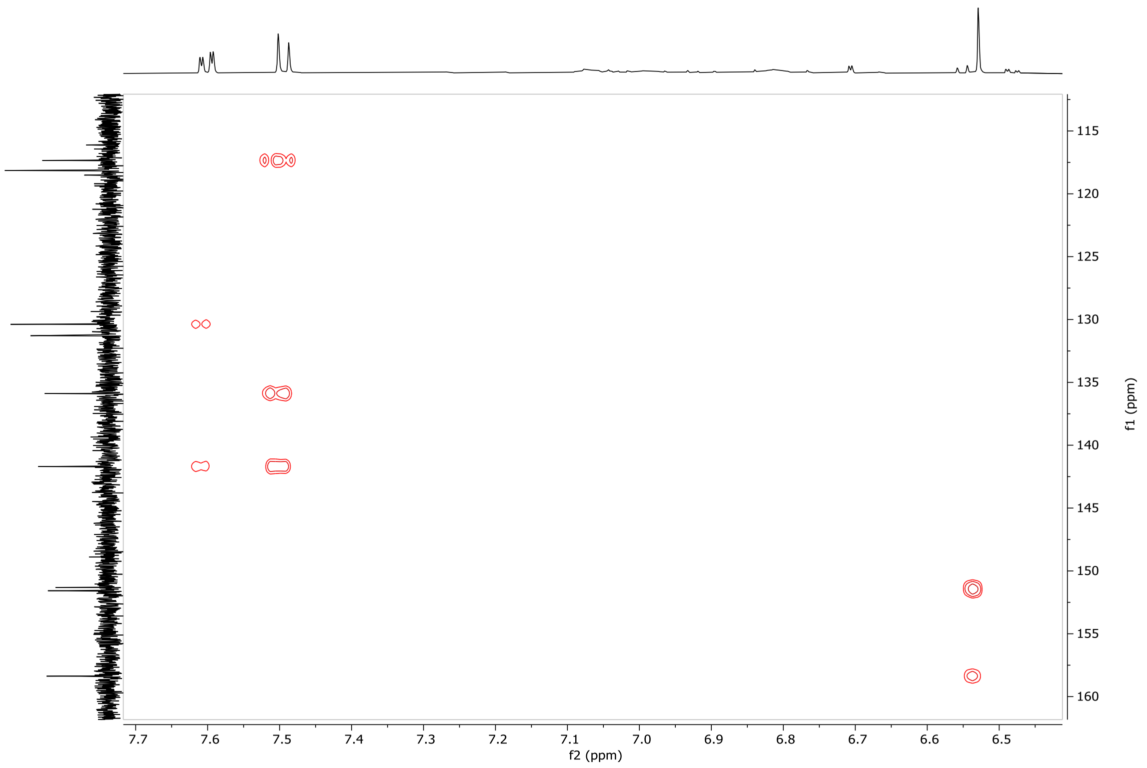

Figure S56. HMBC Spectrum of 2-amino-8-bromo-3H-benzo[b]pyrido[2,3-e][1,4]oxazin-3-one

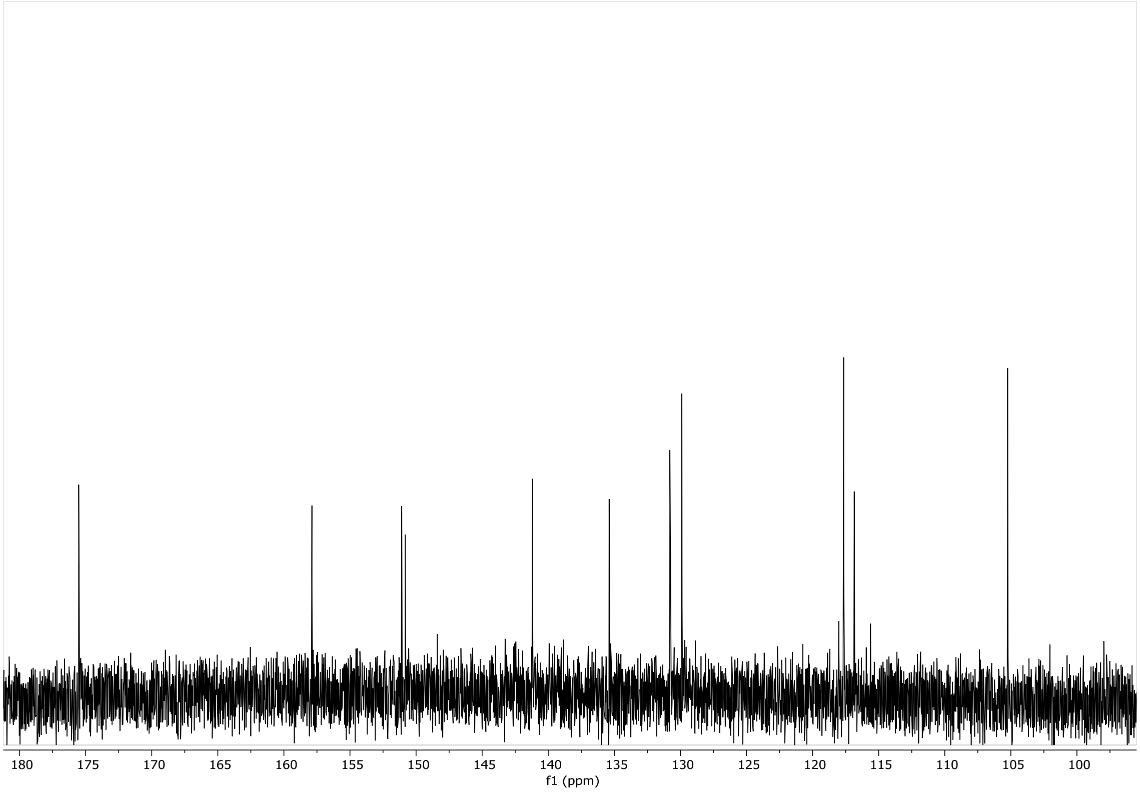

Figure S57. ^{13}C-NMR Spectrum of 2-amino-8-bromo-3H-benzo[b]pyrido[2,3-e][1,4]oxazin-3-one

Structural characterization of 2-amino-8-chloro-3*H*-phenoxazin-3-one (**15**)

2-Amino-8-chloro-3*H*-phenoxazin-3-one (**15**), ^{1}H-NMR (DMSO-d_6, 500 MHz) δ (ppm) 7.75 (1H, d, J=2.6 Hz, H-9), 7.55 (1H, d, J=8.6 Hz, H-6), 7.48 (1H, dd, J=2.6; 9.6 Hz, H-7), 6.99 (2H, bs, NH_2), 6.39 (1H, s, H-4), 6.35 (1H, s, H-1). ^{13}C-NMR (DMSO-d_6, 125 MHz) δ (ppm) 180.3 (C, C-3), 149.1 (C, C-2), 148.8 (C, C-4a), 147.9 (C, C-10a), 140.8 (C, C-5a), 134.7(C, C-9a), 128.6 (C, C-8), 128.0 (CH, C-7), 126.7 (CH, C-9), 117.6 (CH, C-6), 103.7 (CH, C-4), 98.1 (CH, C-1). HRESIMS m/z 247.0289 $[M+H]^+$ (calcd. for $C_{12}H_8N_2O_2Cl$, 247.0274).

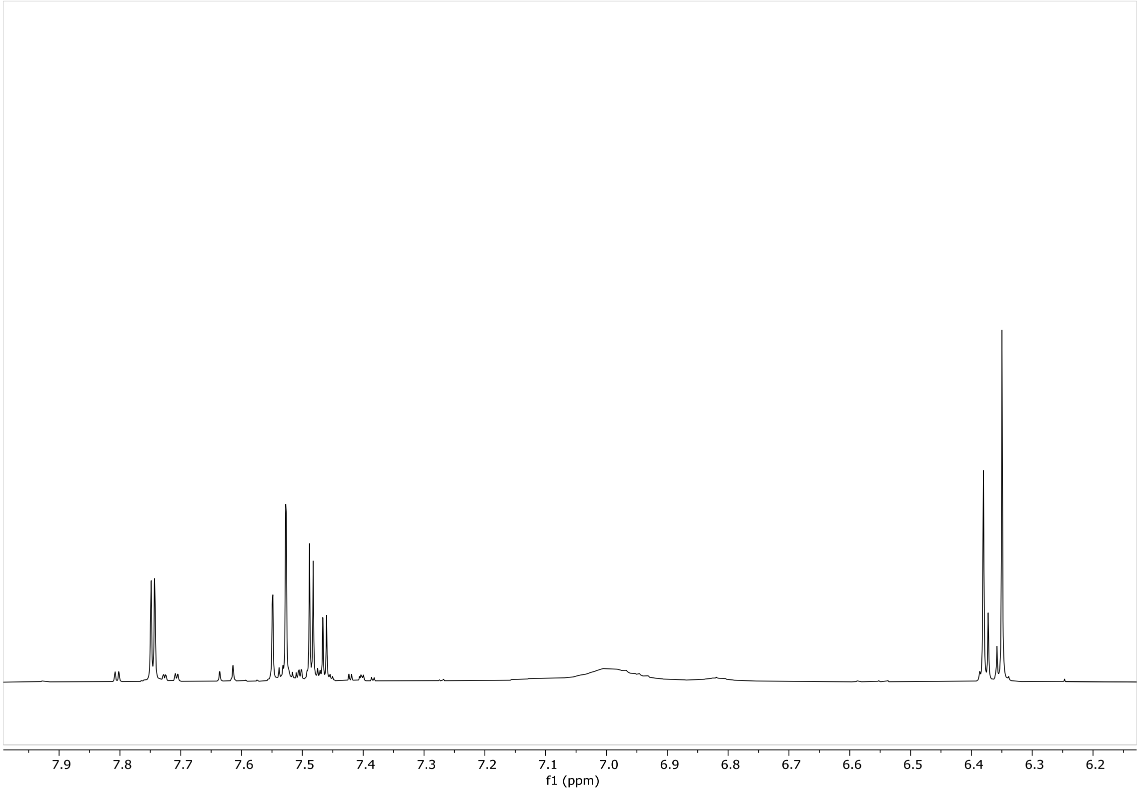

Figure S58. ^{1}H-NMR Spectrum of 2-amino-8-chloro-3H-phenoxazin-3-one

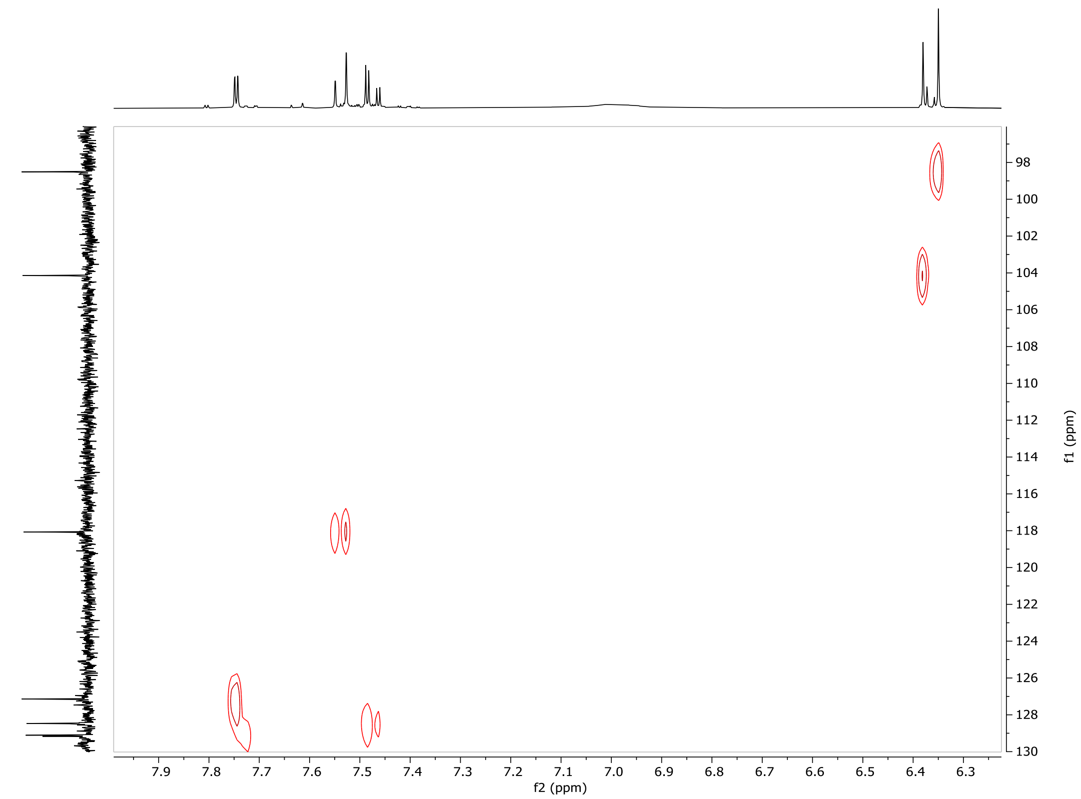

Figure S59. HSQC Spectrum of 2-amino-8-chloro-3H-phenoxazin-3-one

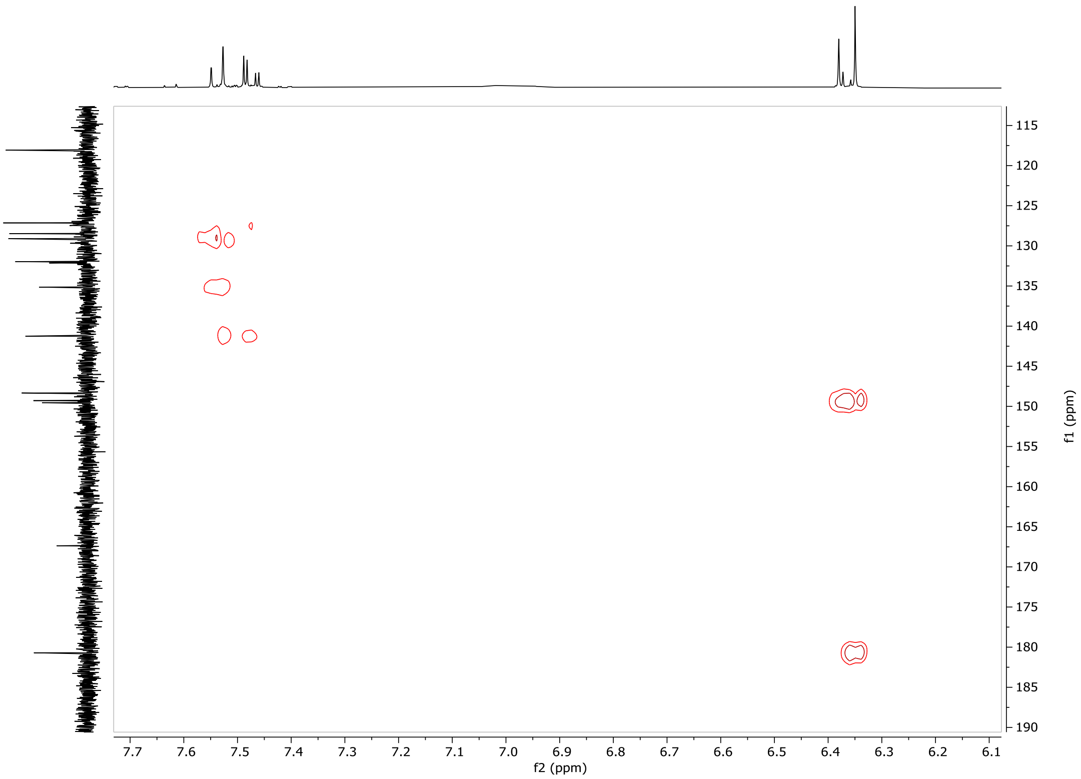

Figure S60. HMBC Spectrum of 2-amino-8-chloro-3H-phenoxazin-3-one

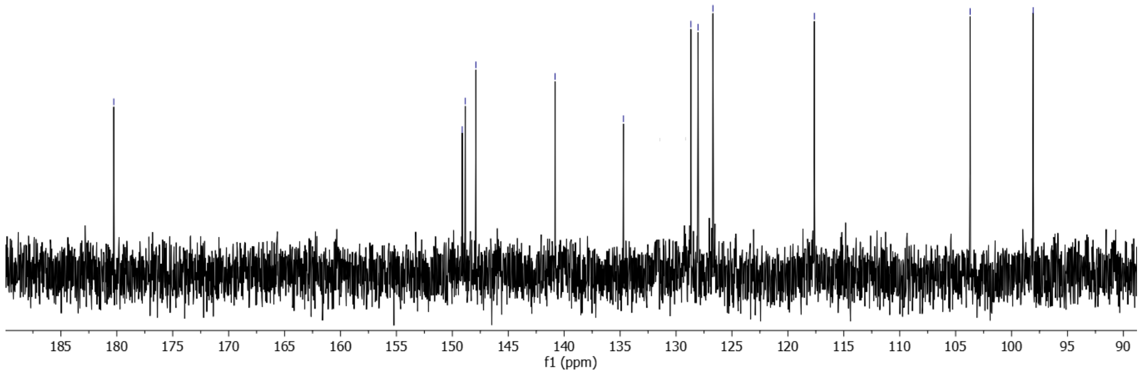

Figure S61. ^{13}C-NMR Spectrum of 2-amino-8-chloro-3H-phenoxazin-3-one

Structural characterization of 2-amino-7-bromo-*3H*-dipyrido[3,2-b:2',3'-e][1,4]oxazin-3-one (12)

2-Amino-7-bromo-*3H*-dipyrido[3,2-b:2',3'-e][1,4]oxazin-3-one (**12**), dark powder, ^{1}H-NMR (DMSO-d_6, 500 MHz) δ (ppm) 8.60 (1H, d, J=2.1 Hz, H-6), 8.31 (1H, d, J=2.1 Hz, H-8), 6.67 (1H, s, H-4); ^{13}C-NMR (DMSO-d_6, 125 MHz) δ (ppm) 175.8 (C, C-3), 156.6 (C, C-2), 148.7 (C, C-10a), 147.7 (C, C-4a), 146.9 (C-H, C-6), 142.8 (C, C-9a), 138.9 (C, C-5a), 126.6 (C-H, C-8), 118.0 (C, C-7), 108.5 (C-H, C-4). HRESIMS *m/z* 290.9250 $[M-H]^-$ (calcd for $C_{10}H_6N_4O_2Br$, 290.9518).

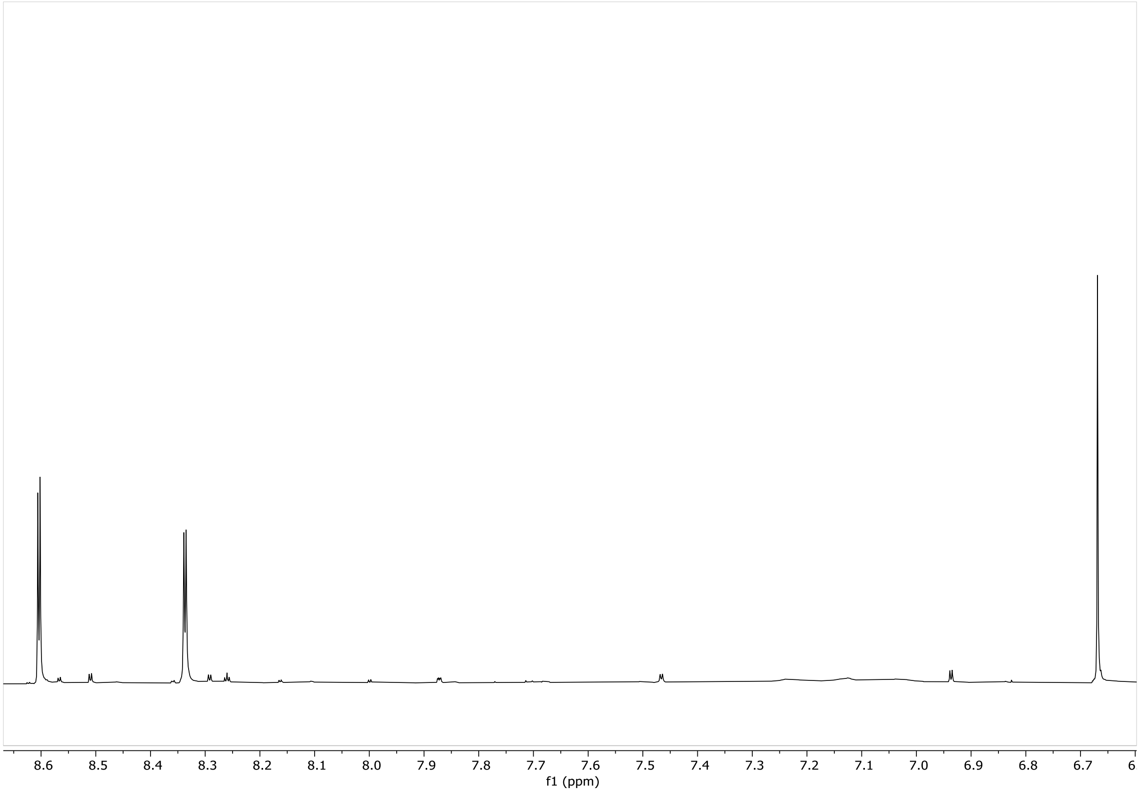

Figure S62. ^{1}H-NMR Spectrum of 2-amino-7-bromo-3H-dipyrido[3,2-b:2',3'-e][1,4]oxazin-3-one.

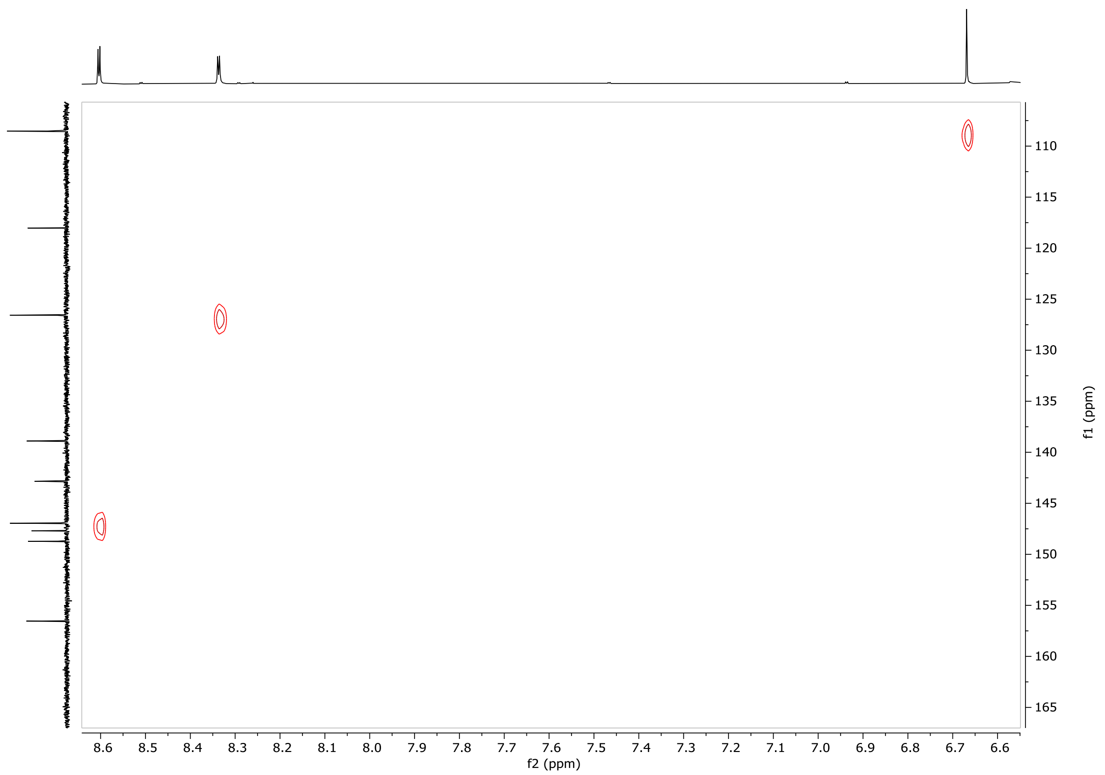

Figure S63. HSQC Spectrum of 2-amino-7-bromo-3H-dipyrido[3,2-b:2',3'-e][1,4]oxazin-3-one.

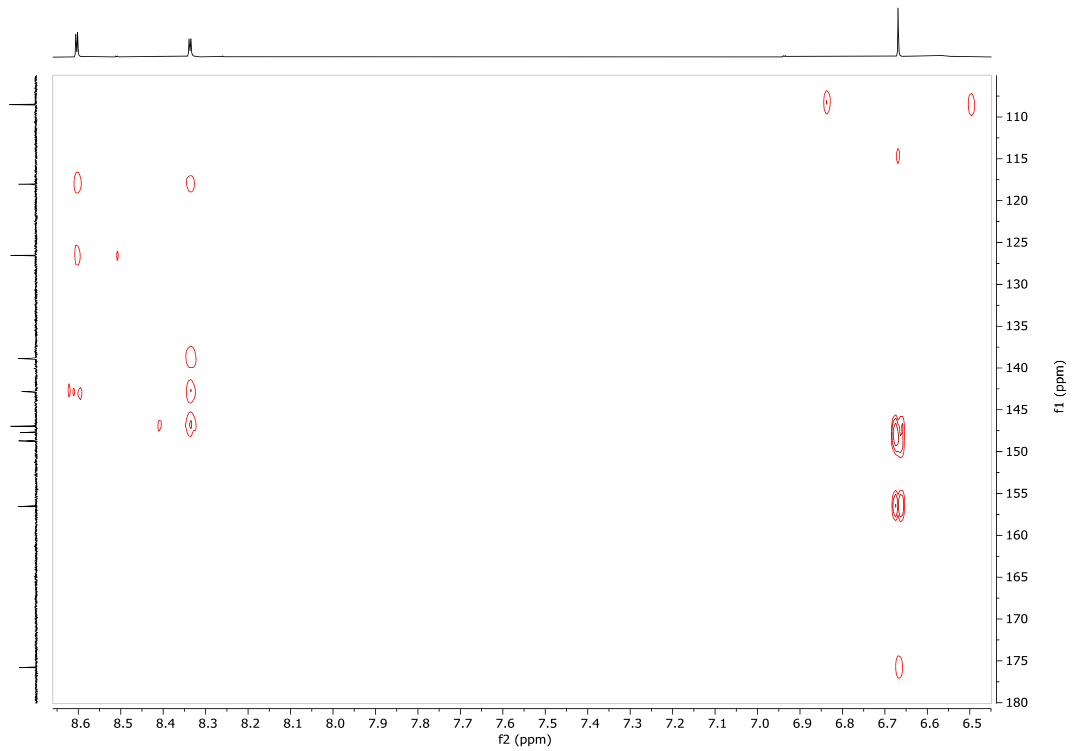

Figure S64. HMBC Spectrum of 2-amino-7-bromo-3H-dipyrido[3,2-b:2',3'-e][1,4]oxazin-3-one.

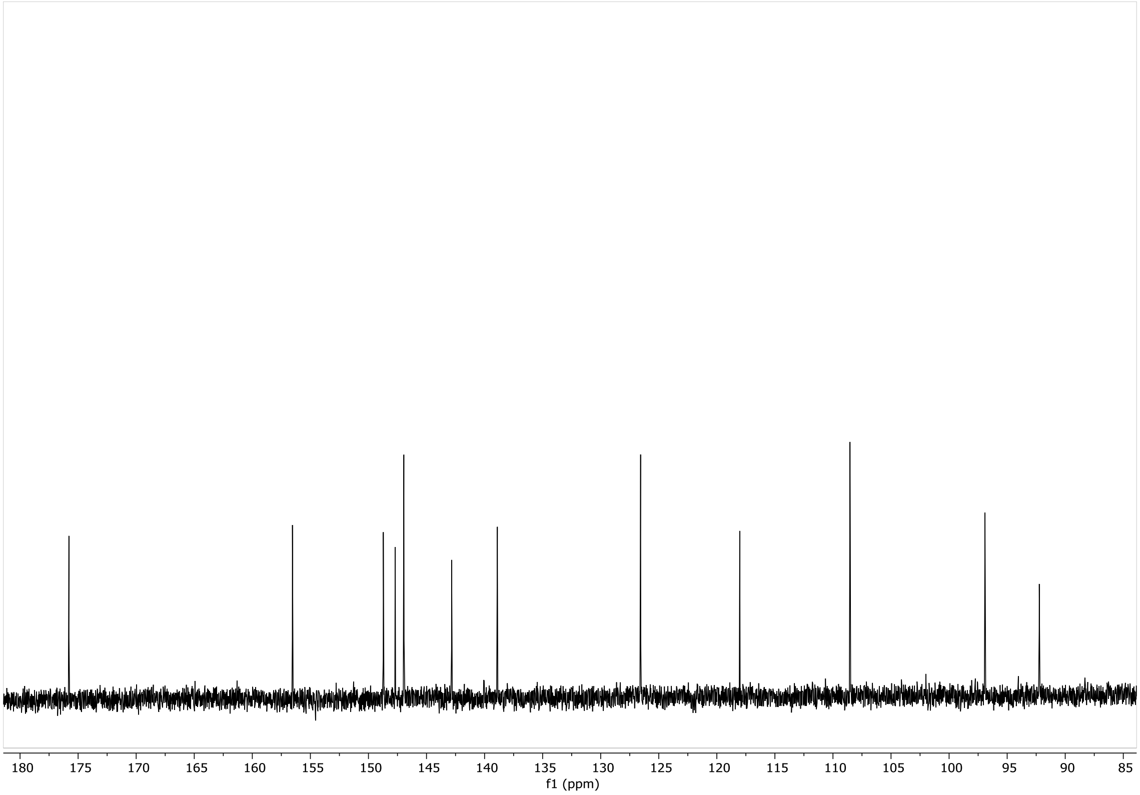

Figure S65. ^{13}C-NMR Spectrum of 2-amino-7-bromo-3H-dipyrido[3,2-b:2',3'-e][1,4]oxazin-3-one
